# Sabertooth carcass consumption behavior and the dynamics of Pleistocene large carnivoran guilds

**DOI:** 10.1038/s41598-022-09480-7

**Published:** 2022-05-02

**Authors:** Manuel Domínguez-Rodrigo, Charles P. Egeland, Lucía Cobo-Sánchez, Enrique Baquedano, Richard C. Hulbert

**Affiliations:** 1 Department of Anthropology, Rice University, Houston, Texas USA; 2 Institute of Evolution in Africa (I.D.E.A.), University of Alcalá, Covarrubias 36, 28010, Madrid, Spain; 3 Department of Anthropology, University of North Carolina at Greensboro, Greensboro, NC USA; 4 Computational Archaeology (CoDArchLab), Institute of Archaeology, University of Cologne, Albertus-Magnus-Platz, D-50923 Cologne, Germany; 5 Archaeology Museum of Alcalá de Henares, Madrid, Spain; 6 Division of Vertebrate Paleontology, Florida Museum of Natural History, Gainesville, FL 32611-7800 USA

**Keywords:** Anthropology, Palaeontology, Ecology

## Abstract

Apex predators play an important role in the top-down regulation of ecological communities. Their hunting and feeding behaviors influence, respectively, prey demography and the availability of resources to other consumers. Among the most iconic—and enigmatic—terrestrial predators of the late Cenozoic are the Machairodontinae, a diverse group of big cats whose hypertrophied upper canines have earned them the moniker “sabertooths.” Many aspects of these animals’ paleobiology, especially their prey preferences and carcass consumption behavior, remain unsettled. While skeletal anatomy, dental morphology and wear, and isotopic profiles provide important insights, the most direct way to resolve these issues is through the fossil remains of sabertooth prey. Here, we report on a taphonomic analysis of an early Pleistocene faunal assemblage from Haile 21A (Florida, USA) that preserves feeding damage from the lion-sized sabertooth *Xenosmilus hodsonae*. Patterns of tooth-marking and bone damage indicate that *Xenosmilus* fully defleshed the carcasses of their prey and even engaged in some minor bone consumption. This has important implications for Pleistocene carnivoran guild dynamics, including the carcass foraging behavior of the first stone-tool-using hominins.

## Introduction

Sabertooth cats were a common sight on the landscapes of Africa, Eurasia, and the Americas from the Miocene to the late Pleistocene^1–4^. [The term “sabertooth” encompasses a diverse group of extinct vertebrates that includes, but is not limited to, the Felidae. Many of these animals share a suite of features—elongated, curved, and laterally flattened upper canines, robust incisors arranged in an arch, reduced number and size of cheek teeth, reduced coronoid processes, and reinforced, vertically oriented mandibular symphyses, among others—that appear independently in several lineages^3^. With that in mind, and for the sake of brevity, we use the term “sabertooth” from this point on to refer to the subfamily Machairodontinae, which includes all felid members of this ancient and very diverse morphotype.] Indeed, if fossil representation is any indication of past population sizes, sabertooths were probably even more numerous than their non-sabertooth cousins. Given the critical role of apex predators in the top-down regulation of modern ecological communities^5–7^, the paleobiology of sabertooths—particularly their hunting and feeding behavior—must factor into any reconstruction of late Cenozoic terrestrial ecosystem evolution (e.g.,^8,9^). Much of our knowledge of the hunting and feeding behavior of sabertooths derives from skeletal anatomy^10–14^, dental morphology and wear^15,16^, and isotopic analyses^17–19^. While most, if not all, sabertooths probably relied on some form of cryptic stalking to pursue prey just as all extant large cats do^20^, these findings nevertheless reveal diversity in body plans, hunting techniques, and prey choice^3,21,22^. The relatively gracile, fleet skeleton of *Homotherium*, for instance, appears better suited to pursuit hunting in open habitats while the hyper-robust *Smilodon* likely relied on ambush tactics in more closed habitats. During a hunt, most sabertooths probably attempted to wrestle and pin their prey to the ground in order to deliver a killing bite to the throat. Their robust incisors meanwhile stabilized the lacerated area to reduce lateral stress on the canines.

One important aspect of sabertooth feeding behavior, namely the method of carcass consumption, requires further investigation. One perspective holds that sabertooths were unwilling, or unable, to thoroughly deflesh the carcasses of their prey. Palmqvist et al.^23^, for example, argue that *Megantereon*, with its cumbersome upper canines and gracile upper carnassial, avoided tooth-on-bone contact and therefore abandoned significant portions of flesh. This is supported by the work of Van Valkenburgh and Ruff^24^, who find that the canines of several species of sabertooth were more susceptible than those of their extant, conical tooth counterparts to breakage from the oblique and medio-laterally directed forces that might accompany contact with bone surfaces. An alternative view is that some sabertooths were quite capable of fully, and sometimes intensively, processing carcasses. Patterns of dental microwear among *Smilodon fatalis* at Rancho La Brea (USA) in fact suggest levels of durophagy comparable to modern lions (*Panthera leo*) and far above those seen in modern cheetahs (*Acinonyx jubatus*)^16^. Clarifying this issue can help gauge how sabertooths influenced the menu of resources available to other consumers, including, at least from the early Pleistocene on, stone-tool-using hominins.

Taphonomic analyses of bone assemblages that are partly or wholly the result of sabertooth feeding offer complementary information that is elusive in other contexts or with other types of data. For example, predictions of sabertooth prey size based on predator–prey body size correlations among modern carnivorans^25–27^ can be tested with tooth mark data that provide direct evidence for consumption (if not actual predation) (e.g.,^28^). Such secure taphonomic linkages are rare despite the co-occurrence of sabertooth fossils with the remains of potential prey species at sites in the Americas^29–33^ and across Afro-Eurasia^34–36^. Here, we offer an analysis of the large mammalian fauna from Haile 21A, an Irvingtonian (early Pleistocene, ca. 1.6–1.0 mega-annum [Ma]) paleo-sinkhole in north-central Florida (USA)^37^. The site features a well-preserved vertebrate fauna, including giant ground sloth, vampire bat, tortoise, tapir, and, most spectacularly, two partial skeletons (the holotype and paratype) of the sabertooth *Xenosmilus hodsonae* and the remains of dozens of flat-headed peccaries of the species *Platygonus vetus*. It is this last association that concerns us here, as it raises the intriguing possibility that the site preserves evidence of sabertooth feeding and, potentially, denning behavior. Indeed, in their original description of *Xenosmilus*, Martin et al.^38^^: 44^ suggest that because the partial skeletons “were found in a cave deposit along with the remains of many large-bodied wild pigs (the peccary *Platygonus*)…[t]his [Haile 21A] was probably a den site and the peccaries a preferred prey.” We test that hypothesis here and, in so doing, evaluate the carcass processing capabilities of *Xenosmilus* within the broader context of sabertooth paleoecology in the Pleistocene.

## Results

Four carnivorans are present in the Haile 21A assemblage: the canids *Canis edwardii* (MNI = 7) and *C. armbrusteri* (MNI = 1) and the sabertooth cats *Smilodon gracilis* (MNI = 2) and *Xenosmilus hodsonae* (MNI = 2). The non-carnivoran taxonomic profile is dominated by *Platygonus vetus* (MNI = 69) to the near exclusion of other large mammals. While *Xenosmilus* and *Platygonus* are represented by more-or-less complete skeletons, the other taxa appear in the assemblage as isolated skeletal elements. About 8% of all peccary bones preserve at least one tooth mark. Nearly all tooth-marked bones preserve single, isolated marks, a pattern that is typical of felids^39–41^ and unlike the intensive gnawing and overlapping tooth marks characteristic of durophagous carnivorans like canids^42,43^ and hyaenids^44,45^.

**Figure 1:**
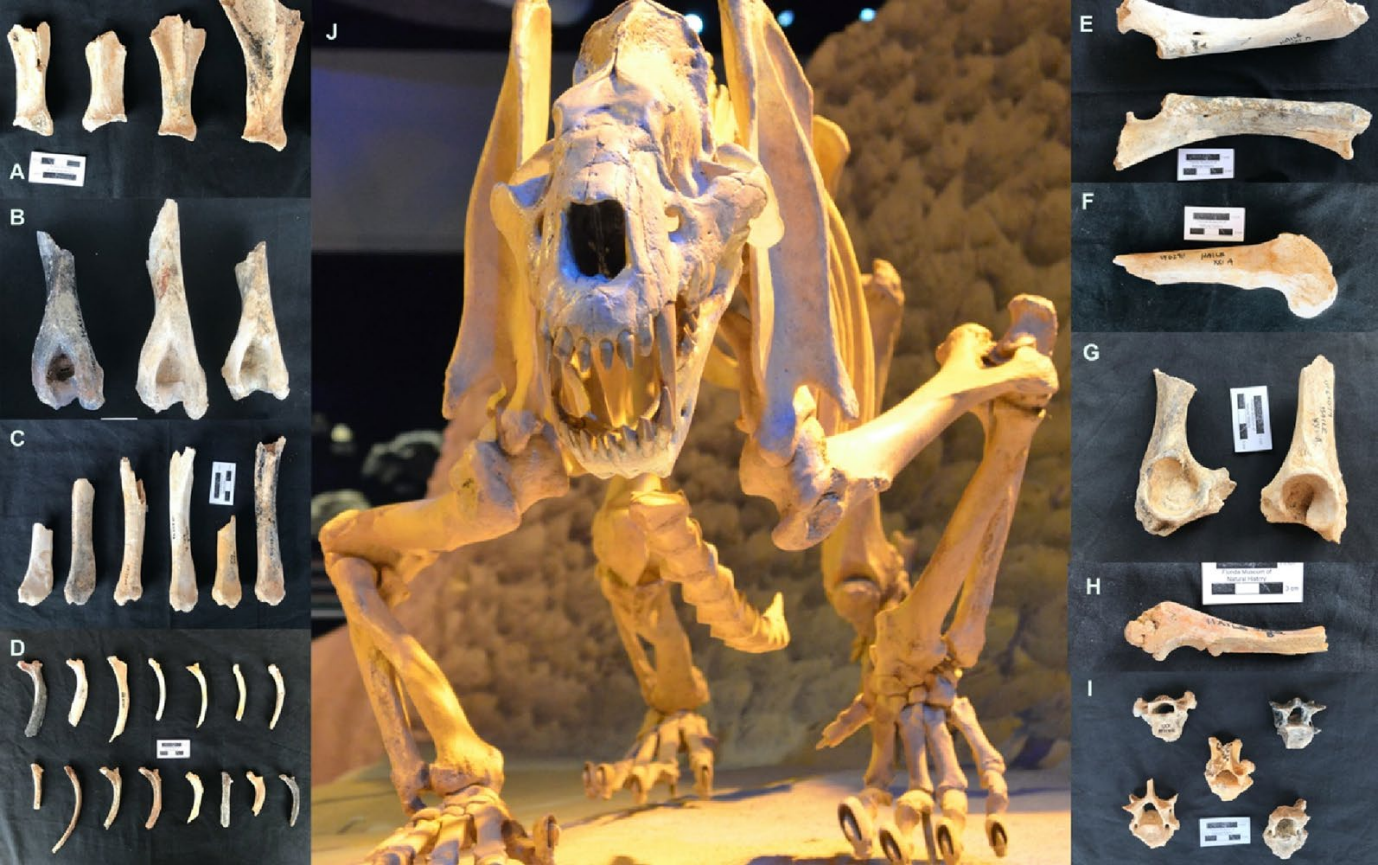
Examples of the carcass consumption capabilities of *Xenosmilus*. Complete defleshing and frequent contact with bone resulting in bone breakage and deletion has been documented on scapulae (**A**), humeri (**B**), tibiae (**C**), ribs (**D**), femora (**F**), innominates (**G**), ulnae (**E, H**), and vertebrae (**I**), with a many of the latter completely fragmented. In contrast, radii appear less affected (**E**), as in carcasses consumed by modern lions (Fig. 2). A complete skeleton of *Xenosmilus hodsonae* is shown in the center, resulting from the combination of the fairly complete holotype skeleton and the paratype skeleton found together at Haile 21A (**J**). This mounted skeleton is currently displayed at the Florida Museum of Natural History (Gainesville).

**Figure 2:**
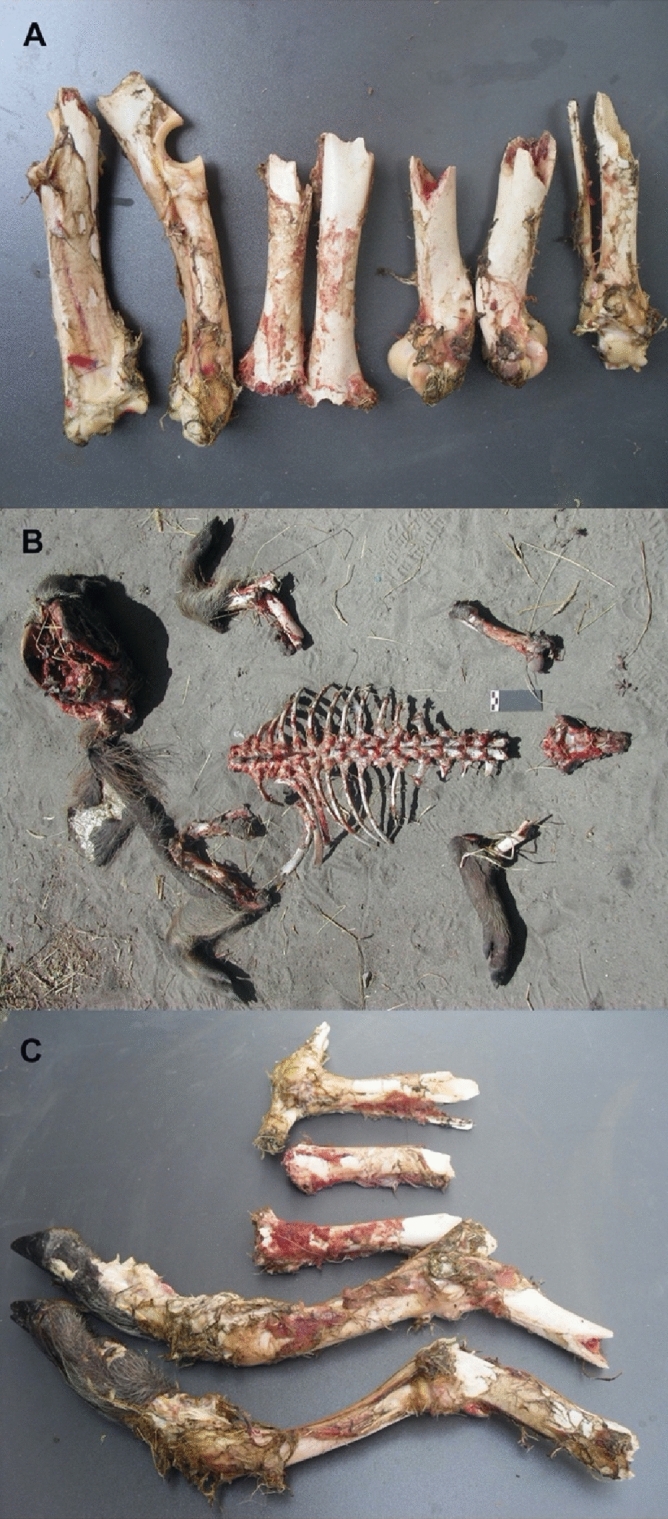
Examples of African warthog carcasses consumed by modern lions (**A–C**). Carcasses are completely defleshed by lions (**B**). Bone breakage is frequent on femora (both ends) and proximal humeri and tibiae (**A, C**). Radii are commonly more intact, with variable modification of the ulnar olecranon. Compare to the pattern seen on the *Platygonus* carcasses modified by *Xenosmilus* (Fig. 1).

Carnivoran damage, much of which also corresponds to what is known to result from the feeding of large felids^40,41,46–48^, appears across all anatomical parts of the Haile 21A peccaries (Fig. 1). Punctures on the neck, and destruction of the blade, of many scapulae indicate the removal of the rhomboid, subscapular, supraspinatus, and infraspinatus muscles. Humeri exhibit furrowing on and, at times, deletion of, the proximal ends, especially around the deltoid crest and tuberosities, which reflects the removal of the deltoid, triceps brachialis, brachialis, and infraspinatus muscles. The distal humeri display furrowing on the caudal aspects of the epicondyles and the medial aspect of the epiphyses, which are origin points for the pronator and the ulnar and carpal flexors. The radii, in contrast, are only lightly damaged. Most modifications occur on the distal ends and are associated with the consumption of the carpal extensor muscle. The olecranon processes of ulnae are consistently furrowed. Many femora are furrowed on both epiphyses and exhibit green breakage on the shafts. The proximal tibiae are frequently modified. Many of the tibial crests are furrowed, and about 50% of the proximal epiphyses have been deleted completely, which indicates the consumption of the biceps femoris, gracialis, sartorius, semitendinosus, and extensor digitorum longus muscles. Consumption of the gastrocnemius and digital flexor muscles is evidenced by furrowing on the calcaneal tuber, and tooth-marking on the lateral facet indicates removal of the cranial tibial muscle. Intensive gnawing of the ilia and ischia reflects consumption of the iliacus, erector spinae, gluteal, adductor, biceps femoris, and semitendinosus muscles. Intensive defleshing of the hindquarters is also evidenced by damage to some sacral vertebrae. Green breaks observed on the rib blades probably reflect the impact of teeth during evisceration and defleshing. Interestingly, most of the rib heads, which are commonly destroyed by scavengers as they consume the axial bones^49,50^, are unmodified. This suggests that the rib cage was articulated to the thoracic column throughout the consumption sequence and after deposition—a typically felid pattern of modification (Fig. 2).

**Figure 3:**
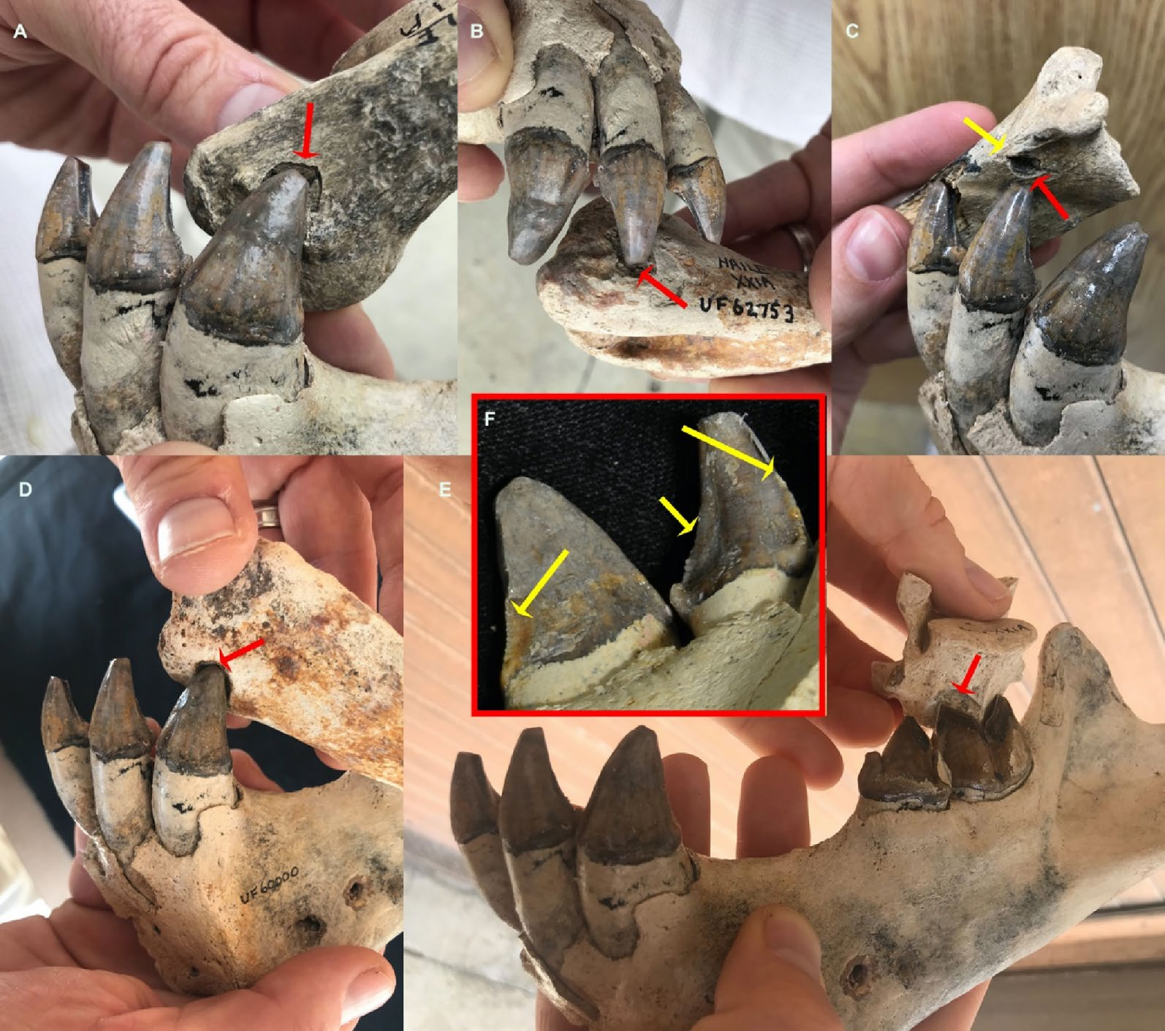
Examples of large tooth marks that fit the size of the *Xenosmilus* anterior dentition (incisors and canines) (**A–D**). These marks are found on the medial side of the distal epiphysis of humeri (**A, B**) and on the neck of scapulae (**C**). They are also documented on the proximal tibia (**D**). A mark that fits the size and shape of the carnassial crest has also been documented on a vertebra (**E**). *Xenosmilus* had very large incisors with serrated edges (**F**) (yellow arrows). They are positioned in a protruding curved arcade. The serrated edges on the carinated incisors and lower canines resulted in frequent bisectioning of the resulting tooth marks (see, for example, the tooth mark on the scapular neck in (yellow arrow in **C**).

The evidence for *Xenosmilus* in particular as the modifier, and possible accumulator, of the peccary remains lies in the size and shape of the preserved tooth marks. The incisors and lower canines of a *Xenosmilus* specimen from Haile 21A match and, in fact, fit snuggly within, tooth marks identified on several peccary bones (Fig. 3). The size and morphology of the fossil tooth marks match quite well with replica marks created with the *Xenosmilus* dentition and differ significantly from replica marks created with the dentitions of *C. edwardii* and *S. gracilis*. Four types of marks are produced by the *Xenosmilus* dentition. The first is triangular in plan view and results from the impact of the mesial cusp of the lower carnassial. Some of these marks appear with a distally oriented striation from the carnassial crest. The second is created by the cusp of the lower fourth premolar and is parallelogram-shaped in plan view. The third consists of half-moon-shaped marks produced by the mesial incisors. The fourth results from the cusps of the incisors and lower canines and appears as a circular or oval-shaped mark. Tooth marks that correspond to all four of these morphologies occur on the peccary skeletons, although the last two are most common. Statistical analysis of the tooth marks and a full description of the bone damage appear in the Supplementary Material.

## Discussion

The arrival of the peccary carcasses into the Haile 21A fissure is difficult to reconstruct. The nearly monospecific nature of the Haile 21A macroherbivore assemblage, dominated as it is by *Platygonus* to the near exclusion of any other taxon, is inconsistent with a natural trap scenario. Natural traps are expected to sample taxa proportional to their representation on the landscape, and it seems unlikely that *Platygonus* made up ~ 90% of the local macroherbivore population. Nor is it likely that this species was uniquely susceptible to entrapment within the Haile 21A fissure. While *Platygonus* is thought to have voluntarily entered caves as a means of shelter^51^, if the walls of the Haile 21A sinkhole were as steep in antiquity as they were upon the site’s discovery, entry into the fissure involved a descent that was likely beyond this ungulate’s scansorial abilities. Save leopards (*Panthera pardus*) in some ecological contexts^35^^: 84–89,^^52^, the repeated transport of prey carcasses to specific locations and, thus, the creation of dense bone accumulations, is rare among modern felids^53–55^. Such behavior is not unknown among sabertooths, however (see below). What is more, the degree and extent of carnivoran damage to the *Platygonus* remains suggests their close spatial relationship with the *Xenosmilus* skeletons is not coincidental. Given the lack of juvenile felid remains, Haile 21A probably did not function as a maternity den. We suggest, however, that *Xenosmilus* utilized the fissure, or the nearby vegetation it probably supported, as a refuge of sorts to which it transported, and where it ultimately consumed, over 60 peccaries (see the Supplementary Material for a full discussion). Regardless of how the peccaries were accumulated, there is little doubt that *Xenosmilus* was responsible for the feeding damage on the carcasses. It is also clear that *Xenosmilus* efficiently and completely consumed nearly all large muscle masses and in the process engaged in some limited durophagy but no bone-cracking. The most common tooth mark morphologies on the *Platygonus* bones correspond closely to those created by the *Xenosmilus* incisors and canines, which indicates that this big cat predominantly used its anterior dentition for defleshing. A reasonable body size analog to *P. vetus* is the modern African warthog, which weighs in at between 50 and 150 kg and falls within Size Class 2 of Brain’s^56^ mammal body size classification scheme (Table 1). Modern lions and tigers (*Panthera tigris*), to which *Xenosmilus* compares favorably in terms of body size, inflict broadly similar levels of damage to carcasses of this size^40,47^ (Fig. 2).

**Table 1:** Mammal body size classes ^56,168^.

Size class	Live body weight (lbs./kg.)	Example (common name)	Example (Linnean binomen)
1	< 50/ < 23	Black muntjac	*Muntiacus crinifrons*
		Red brocket deer	*Mazama americana*
		Thompson’s gazelle	*Eudorcas thompsoni*
2	50–250/23–110	White-tailed deer	*Odocoileus virginianus*
		Common warthog	*Phacochoerus africanus*
		Saiga antelope	*Saiga tatarica*
3	250–750/110–340	Common wildebeest	*Connochaetes taurinus*
		Plains zebra	*Equus quagga*
		Red deer	*Cervus elaphus*
4	750–2000/340–900	American bison	*Bison bison*
		African (cape) buffalo	*Syncerus caffer*
		Moose (elk)	*Alces alces*
5	2000–6000/900–2700	Black rhinoceros	*Diceros bicornis*
		Hippopotamus	*Hippopotamus amphibius*
		Giraffe	*Giraffa camelopardalis*
6	> 6000/> 2700	African elephant	*Loxodonta africana*
		Indian elephant	*Elephas maximus*

Taphonomic data in addition to that from Haile 21A support the contention that Pleistocene homotherines, like their extinct and extant conical tooth cousins, were effective carcass consumers and, unlike most modern big cats, at least occasionally accumulated bones. At Friesenhahn Cave, a late Pleistocene dissolution cavity in southern Texas, twenty-one percent of the large mammal bone fragments retain at least one tooth mark. For the most common large mammal in the assemblage, juvenile Colombian mammoth (*Mammuthus columbi*), those frequencies rise to over 50%^28^. The sabertooth *Homotherium serum*—a lion-sized relative of *Xenosmilus*^38,57^—is the most frequently represented carnivoran in the cave, and the presence of at least 13 juvenile individuals and several old adults strongly suggests a den context^58,59^. At least one series of tooth marks on a mammoth bone matches closely the size and arrangement of the *H. serum* upper incisor row^28^^: 529^. Most tooth marks on the mammoth bones cluster on limb bones and, because carnivoran damage on incompletely consumed elephant carcasses in modern contexts is limited largely to metapodials, long bone epiphyses, and iliac blades^60,61^, the presence of transversely oriented tooth scores on the unbroken shaft sections likely signals intensive defleshing. Most of the mammoth bones are complete or nearly so, and furrowing damage is light. Marean and Ehrhardt^28^^: 537, 544^ thus conclude that this big cat “defleshed carcasses sufficiently completely to result in frequent tooth to bone contact” but that “it was not capable of accessing within-bone nutrients.” This is consistent with data from dental microwear texture analysis, which show that the *H. serum* individuals from Friesenhahn Cave did not use their carnassials to consume or crack bone^19^.

Like other Pleistocene homotherines, *X. hodsonae* and *H. serum* possess large, robust, and labio-lingually thickened incisors, serrated incisors and canines and, at least in the former taxon, a heavily inclined nuchal crest, all of which reflect the importance of the anterior dentition for feeding^28,38,57,62^. These features helped both to stabilize the lacerated area during a killing bite and slice, rake, and pull flesh from bone surfaces. That many tooth marks from Haile 21A and Friesenhahn Cave were created by the anterior dentition provides direct evidence for this behavior. If, as Van Valkenburgh^63^^: 300^ suggests, most tooth fractures among extant carnivorans result from tooth-on-bone contact during feeding (see^16,64^ for an alternative view), the high incisor breakage rates among the Friesenhahn Cave *Homotherium* dentitions, when coupled with the furrowing damage on the Haile 21A peccaries, indicate these big cats did not necessarily or in all cases shy away from bone. It is likely, based on phylogenetic proximity^65^ and shared functional anatomy^66^, that such feeding behavior characterized Pleistocene homotherine taxa (e.g., *Homotherium latidens*) in Afro-Eurasia as well.

This has implications for the dynamics of Pleistocene ecosystems, especially as it pertains to the diet and subsistence behavior of hominins, who, by 2.6 million years ago with the invention of flaked stone tools—and perhaps much earlier through the use of percussive technology—began to exploit the carcasses of animals larger than themselves^67,68^. The broadening of the diet to include such resources thrust hominins, at least occasionally, into diverse guilds of large carnivorans^69–76^. While sabertooths were no doubt important guild members, the impact of these large cats on how hominins foraged for carcasses in the Pleistocene remains unsettled. While there is general agreement that the dental morphology of Pleistocene sabertooths is an adaptation for rapid and efficient flesh consumption, some nonetheless go on to argue that the relatively fragile cheek and canine teeth of sabertooths forced them to abandon significant amounts of flesh on kills that could subsequently be exploited by passive scavengers, including hominins^23,36,71,77–79^. The evidence from Haile 21A and Friesenhahn Cave indicates that this is not the case, at least when it involves the consumption by homotherines of carcasses the size of *P. vetus* or a juvenile mammoth.

It has also been argued that sabertooths ostensibly reluctant to impact bone surfaces with their canines and carnassials may have relinquished intact marrow cavities to potential scavengers with the dental armament or technological wherewithal to breach them^4^^: 375,^^77,80,81^. The data from Haile 21A provides partial support for this scenario: while *Xenosmilus* did not fragment peccary long bones, it was able to expose the marrow cavities of the upper limb bones and tibiae, but not the radii, of these Size Class 2 carcasses. Peccaries do not have the robust, elongated metapodials characteristic of bovids, equids, and other taxa consumed by large felids, but we suspect that *Xenosmilus* and other homotherines left such thick-walled, flesh- and marrow-poor bones largely intact. The completeness of the juvenile mammoth long bones from Friesenhahn Cave shows that within-bone nutrients from *Homotherium* kills of Size Class 4 or 5 animals were probably readily available to scavengers. This is all consistent with what is known for the comparably-sized lion, which regularly, inconsistently, and rarely if ever accesses, respectively, the marrow cavities of Size Class 1 and 2, Size Class 3, and Size Class 4 and 5 carcasses^40,80^^: 386,^^82–84^.

A passive scavenger, then, be it hominin or otherwise, would encounter almost no flesh and only a smattering of untapped marrow cavities among the Size Class 2 peccary carcasses left behind by *Xenosmilus* at Haile 21A and limited flesh and most of the within-bone nutrients from the Size Class 4 juvenile mammoth bones transported by *Homotherium* to Friesenhahn Cave. While further taphonomic data are needed, it stands to reason that homotherines abandoned Size Class 3 carcasses with very little flesh and intermediate to large amounts of marrow, grease, and other within-bone nutrients. A more ample meal would require either kleptoparasitism—a formidable task in the case of *Homotherium* if it did indeed feed in groups^12^—or hunting. For those species able to appropriate carcasses and/or crack larger bones, homotherine kills of Size Class 3 and especially Size Class 4 and 5 animals probably furnished some flesh and a good deal of within-bone nutrients on a routine basis.

In the Pleistocene of Afro-Eurasia, several species of hyena, especially the giant bone-cracking *Pachycrocuta brevirostris*, were probably well suited to take advantage of this resource^85–87^. Stone-tool-wielding hominins too may have benefited under these circumstances, especially if, as some hypothesize, the initial shift to large mammal consumption in the late Pliocene developed within the context of percussion-based scavenging of within-bone nutrients^67^. In the earliest Pleistocene, evidence for intermittent consumption by hominins of flesh and marrow from various mammals coincides with the invention of sharp-edged, flaked stone tools^88,89^. Soon thereafter, some groups of hominins began to consistently access carcasses up to and including Size Class 3. However, the amount of flesh butchered from those carcasses—as indicated by the frequency and location of stone tool cut marks—would be difficult to obtain by passively scavenging the kills of homotherines (or any other big cat, for that matter)^90,91^. This reveals a hominin forager capable if not of hunting (evidence for which remains indirect^92^) then at least of confrontational scavenging^93^. The same pattern of carcass access and utilization persists into later stages of the early Pleistocene^94^ and, at some sites, even expands to include Size Class 4 and 5 animals^95,96^.

Hominins arrived in the Americas as apex predators to join a carnivoran guild that, at least after the extinction by the late Pliocene of the borophagine canids, lacked a bone-crusher analogous to the Old World hyaenids. In North America, this role may have been filled in part by canids like gray wolves (*Canis* lupus) and dire wolves (*Canis dirus*) and/or the hyaenid *Chasmaporthetes*. Experiments with wild wolves show that they comminute the long bones of Size Class 2 and, less frequently, Size Class 3 and 4 animals^42,97–100^. However, all three taxa were (and, in the case of gray wolves, still are) hypercarnivorous pursuit hunters whose dentitions reflect an emphasis on shearing and slicing rather than intensive bone-breaking^64,87,101,102^. Even the enormous tremarctine bear *Actodus simus*, while no doubt an intimidating presence, was an omnivore that likely did not engage in substantial amounts of flesh or bone consumption^103^. Relative to those on Eurasian landscapes, then, the marrow from abandoned homotherine kills, especially of larger prey, probably persisted longer and/or was consumed less fully by scavengers, much like what is seen in modern ecosystems where dedicated bone-cracking species are rare or absent (for an example, albeit in an ecosystem heavily modified by humans, see^104^).

Functional analyses of sabertooth cranio-dental morphology reveal a diversity of potential feeding behaviors and killing strategies^62,105,106^, so the above discussion cannot necessarily be generalized to non-homotherine taxa. For example, *Megantereon*, especially the highly derived *M. whitei* with its combination of extraordinarily long upper canines, reduced or even vestigial premolars (a trait that nevertheless characterizes some homotherines), and reduced or absent dental serrations, may not have been capable of processing carcasses to the same extent as *Xenosmilus* and *Homotherium*^23,79^. On the other hand, dental microwear texture analyses of M_1_s and morphological studies of premolars indicate that durophagy among, respectively, *Smilodon* and several south African sabertooths, including *Megantereon*, was on par with that of modern lions^16,107^. Tooth breakage rates among *Smilodon* fossils at Rancho La Brea also suggest frequent tooth-on-bone contact and intensive carcass processing^15,108^. This highlights the need for additional taphonomic analyses of faunal assemblages securely linked to the feeding behavior of sabertooths.

## Conclusion

As large, apex predators, sabertooths likely played an important role in the top-down regulation of Pleistocene ecosystems. More specifically, their hunting and feeding behaviors influenced, respectively, the demography of prey species and the menu of items available to other consumers. Studies of skeletal anatomy, dental morphology and wear, and isotopic profiles provide important insight into the range of sabertooth behavioral capabilities. Taphonomic analyses of the fossil remains of sabertooth prey reveal how these capabilities were actualized at specific times and places. The evidence from Haile 21A and Friesenhahn Cave indicates that homotherine sabertooths probably hunted and most certainly consumed effectively the carcasses of animals ranging in size from peccaries to juvenile mammoths. Most passive scavengers on Pleistocene landscapes, then, would find the kills of these felids to be insignificant and irregular sources of flesh. To those able to crack bones, the abandoned kills of Size Class 3 and larger animals probably offered meaningful amounts of marrow and other within-bone nutrients. Scavengers hoping for a more complete menu of foods would need to drive these big cats from their kills early in the feeding sequence. Early Pleistocene stone-tool-using hominins likely could have gained quick access to the kills of homotherines and other sabertooths via confrontational scavenging, although evidence is mounting that they were successful hunters in their own right well before their incremental ascent to apex predator status in the middle Pleistocene^109–112^. Local ecological circumstances no doubt dictated how, and how often, these kills were encountered and utilized by hominins and other consumers. However, given the structures of, and the timing and nature of hominin encroachment on, their respective Pleistocene carnivoran guilds, the character of these interactions probably differed significantly among the Americas, Eurasia, and Africa. More taphonomic data from relevant faunal assemblages are required to characterize the full range of sabertooth carcass consumption capabilities. We nevertheless predict that additional studies will show that most sabertooths, at least among the derived Pleistocene forms, were fully capable of defleshing the carcasses of their prey (cf.^72^^: 113^), and those comparable in size to lions could engage in at least lion-like levels of durophagy.

## Background and methods

### Geological setting

Much of modern Florida lies atop a massive carbonate platform that initially formed in the warm, shallow seas of the Late Jurassic and Early Cretaceous. Carbonate sedimentation continued periodically as sea levels fluctuated throughout much of the Paleocene, Eocene, and Oligocene^113^. Over the last 30 million years or so, falling sea levels and subsequent dissolution enlarged the joints, cracks, and bedding planes within the Florida Platform’s limestones and dolomites to create a complex, eogenetic karstic landscape riddled with fissures, sinkholes, and caves^113–118^, many of which later became receptacles for terrestrial materials^118–122^. The resulting fossiliferous deposits preserve important sedimentological, faunal, and botanical records of environmental change from the late Oligocene and into the Holocene^121,122–127^. Indeed, the prevalence of these geological features is largely responsible for Florida’s incredibly rich record of Plio-Pleistocene terrestrial vertebrates, which is among the most complete in eastern North America^33,127–131^.

### History of collection and research at Haile 21A

In the 1950s, large-scale commercial mining began to target the Eocene-aged limestones of the Ocala Formation near the now-defunct town of Haile, Alachua County, Florida. The fossiliferous dissolution features revealed by these operations, referred to collectively as the Haile Quarries or Haile Complex and individually by alpha-numeric Quarry and Locality designations (e.g., Haile 7A), contain rich collections of Miocene, Pliocene, and Pleistocene vertebrate fossils^124,131–151^. At one of these sites—later named Haile 21A—mining operations exposed a clay plug some 2.5 to 4 m in width. Initial exploration of the clays by commercial fossil collectors in early 1983 uncovered the remnants of an ancient sinkhole with near-vertical walls. Their subsequent excavations unearthed two partial skeletons of the sabertooth *Xenosmilus hodsonsae* and numerous remains of the extinct flat-headed peccary *Platygonus vetus*. This work ceased once it was no longer possible to readily climb into or out of the sinkhole without risking a major collapse of the now-unsupported limestone walls. Some of the fossils from this first phase of excavation were later donated to the Florida Museum of Natural History, which in October and November of 1983 and February and March of 1984 undertook controlled excavations at the site. After the limestone that once surrounded the upper portion of the excavated deposit was removed with assistance from the local mining company, it became apparent that the lower recesses of the sinkhole subdivided into four narrower fissures. All macrofaunal specimens identifiable to skeletal element (including long bone fragments without epiphyses) were recovered from the fissures’ clay and limestone rubble matrix. While smaller fragments and microfauna were not systematically collected, water screening was carried out on a sample of the sediments. Occasional visits after March of 1984 to the Haile 21A spoil piles turned up additional fossils, but no further in situ excavations were conducted. By the late 1990s, the site and any remaining fossiliferous deposits had been destroyed fully by mining operations.

In the first published description of the Haile 21A locality, Morgan et al.^148^ named a new species of vampire bat and noted the overwhelming dominance of the vertebrate fauna by *Platygonus*. Morgan^152^ later reported a large number of fossils of the cave-dwelling bat *Myotis austroriparius* at the site, although this sample remains undescribed. Morgan and Hulbert^37^^: 77^, in their summary of late Blancan and Irvingtonian vertebrate faunas from Florida, listed the large machairodont recovered from Haile 21A as “an apparently undescribed species of the sabercat *Homotherium*.” Several other papers in Hulbert et al.^153^ referred briefly to fossils from Haile 21A, including Berta^154^, Hulbert^155^, and Wright^156^. The Haile 21A machairodont was formally named as a new genus and species, *Xenosmilus hodsonae*, by Martin et al.^38^, who followed up this preliminary report with a more detailed description of the taxon^157^. No detailed morphometric analysis or anatomical description of the site’s *Platygonus* remains has been carried out.

### Faunal identification and quantification

The Haile 21A *P. vetus* remains are curated by skeletal element in the Division of Vertebrate Paleontology at the Florida Museum of Natural History (FLMNH). All element identifications were confirmed (and, in some cases, modified) based on comparisons with collared peccary (*Pecari tajacu*) skeletons housed in the Mammals Collection of the FLMNH’s Department of Natural History. All Size Class 2 artiodactyl rib specimens were assumed to belong to *P. vetus*, as no other similarly sized artiodactyl is present in the assemblage. Four measures of skeletal abundance were employed. The Number of Identified Specimens (NISP) is simply a raw count of specimens (that is, a whole skeletal element or fragment thereof) identified to a particular taxon and/or skeletal element. The Minimum Number of Elements (MNE) is an estimate of the lowest number of individual and originally intact skeletal elements required to account for the specimens identified to a particular skeletal element. We used a manual overlap approach to calculate MNEs whereby the left and right specimens (for paired elements; e.g., humeri) or all specimens (for unpaired elements; e.g., cervical vertebrae) of each skeletal element were laid out together on a table to identify refits (that is, specimens that must derive from the same skeletal element) and anatomical overlaps or specimens from individuals of different sizes and/or ontogenetic ages (that is, specimens that must derive from different skeletal elements). The Minimum Number of Individuals (MNI) is an estimate of the lowest number of originally intact skeletons required to account for the specimens identified to a particular skeletal element and taxon. We calculated MNIs for each skeletal element by again laying out left and right specimens (for paired elements) or all specimens (for unpaired elements) together on a table to determine, on the basis of size, ontogenetic age, and other morphological details, if specimens could derive from the same individual. The highest MNI was taken as an estimate of the total number of individuals from each taxon represented in the Haile 21A assemblage. The last measure of abundance we employed is the minimum animal unit (MAU). The MAU was calculated by dividing the MNE for a particular skeletal element by the number of times that skeletal element occurs in a complete peccary skeleton. The MNE was divided by two for paired elements, by 28 for ribs, by one for atlas vertebrae, and so on. These MAU values were then standardized (%MAU) to the highest MAU in the assemblage.

### Reconstruction of the taphonomic history of the Haile 21A assemblage

We conceive the taphonomic history of the Haile 21A faunal assemblage as a series of non-mutually exclusive phases that involve unique combinations of taphonomic actors and their resultant traces^158,159^^: 559^. Our focus is the biostratinomic phase between the death of an animal and its final burial and, more specifically, the nutritive component of that phase, which is the narrow window of time when bones retain edible tissue. To understand the role of *Xenosmilus*—or any other nutritive phase taphonomic actor—in the accumulation of the Haile 21A peccaries, two taphonomic traces are of concern: breakage and surface modifications. Felids and other carnivorans fracture bones in their attempts to remove flesh and/or gain access to within-bone nutrients such as marrow. Such nutritive phase breakage typically results in curved or V-shaped fracture outlines, obtuse or acute fracture angles, and smooth fracture surfaces, while non-nutritive breakage produces transverse fracture outlines, right-angle fractures, and ragged or stepped fracture surfaces^160^. These features are most visible on the diaphyses and metaphyses of long bones and thus are difficult to apply to skeletal elements or portions that lack thick cortical bone (e.g., long bone epiphyses, vertebrae). What is more, these distinctions are not absolute, and any single specimen may preserve features of both types of breakage. Non-nutritive breakage from excavation and/or curation often results in fracture surfaces that differ in color from those of ancient (nutritive or non-nutritive) fracture surfaces.

Nutritive phase trauma to bone cortices, what we call here bone surface modifications (BSMs), provides additional information on the taphonomic history of a faunal assemblage. All cortical surfaces in the Haile 21A assemblage were carefully observed for conspicuous and inconspicuous BSMs using × 15 to × 20 hand lenses under a strong, oblique light source. Of most concern are the four types of marks created by tooth-on-bone contact during carnivoran feeding^161^^: 44^: furrows, punctures, pits, and scores. Furrows appear as wide, deep, and long tooth marks on soft cancellous bone. Sustained gnawing, chipping away, and/or licking of exposed bone surfaces eventually result in crenulated, rounded, and/or polished edges and partial or full destruction of less dense skeletal elements or portions (e.g., vertebral centra and long bone epiphyses). Punctures occur when a single tooth or a set of teeth penetrates completely through a flat bone (e.g., scapular blade) or into the medullary cavity of a long bone. Shallow depressions, or tooth pits, and elongated channels, or tooth scores, are created when teeth contact a bone surface but fail to penetrate fully into the underlying cavity. The attribution of these types of marks to carnivoran agency is complicated by the fact that microbial bioerosion can create shallow depressions and channels that closely resemble the pits and scores created by carnivore teeth. Experimental work, however, does reveal that unlike microbial bioerosion, tooth scores are symmetrical along their longitudinal axes, occur perpendicularly or at oblique angles to the long axis of a bone, penetrate through several layers of cortical lamellae, and show crushing within and along their margins^162^. Given this potential equifinality, and especially because BSMs attributable to microbial activity are present on the Haile 21A bone surfaces, only those tooth mark identifications confirmed by three analysts (MDR, CPE, LCS) were included in the analysis.

When used in concert with actualistic studies of carnivore activity, it is possible to apply patterns of bone breakage, in addition to the frequency, anatomical patterning, dimensions, and shape of tooth marks, to reveal the type(s) of carnivoran(s) involved in the accumulation of a faunal assemblage. We first assigned each long bone specimen exhibiting nutritive phase breakage to a “taphotype,” which is a morphological category defined by the degree of epiphyseal destruction and the location of tooth-marking. Studies show that taphotype profiles vary with the degree of durophagy and, thus, by carnivoran taxon^163^. Tooth mark frequencies were calculated by first counting the number of specimens with at least one tooth puncture, pit, and/or score. These values were then divided by the NISP for each taxon (e.g., peccary), skeletal element (e.g., humerus) and, for long bones, skeletal element portion (e.g., proximal humerus). The Haile 21A %NISP tooth mark frequencies were then compared to those from actualistic samples of carcasses consumed by known carnivorans, which show that flesh specialists like felids tend to produce lower frequencies than do bone-crackers like hyaenids^44^. The length (maximum dimension) and width (maximum dimension perpendicular to length) of tooth pits and the width (maximum dimension) of tooth scores were measured with digital calipers to the nearest 0.01 mm. Because teeth can more easily penetrate thinner, less-dense bone than they can thicker, denser bone, tooth marks on cancellous bone or bone portions are typically much larger than those on cortical bone or bone portions. For that reason, we treated tooth mark dimensions on the diaphysis, metaphysis, and/or dense epiphyseal portions (e.g., distal humerus) of long bones, those on thin-walled portions of long bones with extensive trabeculae (e.g., the femoral trochanters, the humeral tuberosities), and those on axial elements as three distinct analytical samples.

We also generated a comparative sample of tooth marks created by the dentitions of three large carnivorans present in the Haile 21A fauna: *Xenosmilus hodsonae*, *Smilodon gracilis*, and *Canis edwardii*. To do so, we impressed each of the upper and lower teeth from each species onto a soft clay plaque. All marks were produced by fossils from the Haile 21A assemblage except those from the P^4^ of *Smilodon*, which derive from the cast of a specimen from a nearby locality. The clay plaques were set inside an oven at 120°F for several hours and, once dry, all marks were measured with the same protocol outlined above for the fossil marks. The initially soft clay substrate most closely approximates the resistance of cancellous, rather than cortical, bone, so we limited our comparisons of the clay marks to those on trabecular portions (as defined above) of the Haile 21A peccary bones. To maximize comparability, great care was taken to ensure that the clay tooth marks were no deeper than those observed on the fossil remains. The Haile 21A tooth mark dimensions, both fossil and clay, were compared to actualistic data, which demonstrate that while tooth mark size is not diagnostic of specific carnivoran taxa, it can discriminate large animals (e.g., spotted hyenas, lions) from small animals (e.g., foxes, coyotes)^164^. Finally, we qualitatively compared digital photographs of the plan-view shapes of the clay tooth marks to those of the Haile 21A fossils. We also noted non-nutritive phase damage when present, including subaerial weathering^165^, abrasion/polish^166^, and manganese/carbonate encrustation^167^.

## Supplementary Information

Supplementary Information.
