# Supplementary material for: Sabertooth carcass consumption behavior and the dynamics of Pleistocene large carnivoran guilds
Source: Sci Rep. 2022 May 2;12:6045. doi: 10.1038/s41598-022-09480-7 (PMC9061710; doi:10.1038/s41598-022-09480-7)
Supplement: Supplementary file 1 — Supplementary Information. [file 41598_2022_9480_MOESM1_ESM.docx]

**Sabertooth carcass consumption behavior and the dynamics of Pleistocene large carnivoran guilds**

**Supplementary Information**

**Taxonomic and skeletal representation**

While a handful of other large mammals are represented at Haile 21A, the site’s faunal assemblage is dominated by the extinct flat-headed peccary *Platygonus vetus* (Table S1). The maximum estimate for the Minimum Number of Individuals (MNI) represented by the peccary remains is 69 and is provided by left radii. The skeletal part representation for *P. vetus* in terms of NISP, MNE, and MAU is presented in Tables S2 and S3. A total of 2,094 specimens were identified to skeletal element. All parts of the skeleton are represented, although small compact bones and axial bones are much rarer relative to the major long bones (humeri, radii, femora, and tibiae). Skulls (crania and mandibles), scapulae, innominates, and metapodials are represented in intermediate frequencies.

Table S1. NISP counts and MNI estimates for large mammals from Haile 21A.

| Taxon | NISP | MNI |
| --- | --- | --- |
| *Eremotherium eomigrans* | 1 | 1 |
| *Canis edwardii* | 131 | 7 |
| *Canis armbrusteri* | 1 | 1 |
| *Smilodon gracilis* | 16 | 2 |
| *Xenosmilus hodsonae* | 60* | 2 |
| *Platygonus vetus* | 2094 | 69 |
| *Palaeolama mirifica* | 14 | 1 |
| *Odocoileus virginianus* | 1 | 1 |
| *Tapirus haysii* | 2 | 2 |
| *Equus* sp. | 25 | 3 |
| *Cuvieronius* sp. | 9 | 1 |

*The paratype (UF 60000), which is curated at the Florida Museum of Natural History, consists of 20 specimens. The 40 specimens for the holotype (BIOPSI 101) are described and listed in Martin et al. [1]

Table S2. Skeletal element representation by NISP for *Platygonus vetus* from Haile 21A.

| **Element** | **NISP** |
| --- | --- |
|  |  |
| Cranium | 240 |
| Mandible | 132 |
| Rib | 191 |
| Sternal element | 8 |
| Vertebra | 320 |
| Atlas | 24 |
| Axis | 14 |
| Other cervical | 61 |
| Thoracic | 88 |
| Lumbar | 96 |
| Sacrum | 20 |
| Caudal | 17 |
| Innominate | 55 |
| Scapula | 57 |
| Humerus | 122 |
| Radio-ulna | 127 |
| Ulna* | 50 |
| Carpal | 75 |
| Metacarpal | 76 |
| Femur | 97 |
| Tibia | 120 |
| Fibula | 21 |
| Tarsal | 138 |
| Calcaneus | 55 |
| Astragalus | 43 |
| Other tarsal | 40 |
| Metatarsal | 59 |
| Metapodial | 40 |
| Phalanx | 140 |
| Patella | 18 |
| Sesamoid | 8 |
| Total | 2094 |

*Detached from the radius.

Table S3. Skeletal element representation by MNE and MAU for *Platygonus vetus* from Haile 21A.

| **Element** |  | **MNE** |  | **MAU** | **%MAU** |
| --- | --- | --- | --- | --- | --- |
|  | Left | Right | Total |  |  |
| Cranium |  |  | 27 | 27.0 | 47.0 |
| Mandible | 35 | 32 | 67 | 33.5 | 58.3 |
| Rib | 77 | 63 | 140 | 5.0 | 8.7 |
| Sternal element |  |  | 8 | 1.3 | 2.3 |
| Vertebra |  |  |  |  |  |
| Atlas |  |  | 21 | 21.0 | 36.5 |
| Axis |  |  | 14 | 14.0 | 24.3 |
| Other cervical |  |  | 61 | 12.2 | 21.2 |
| Thoracic |  |  | 84 | 6.0 | 10.4 |
| Lumbar |  |  | 96 | 16 | 27.8 |
| Sacrum |  |  | 13 | 13.0 | 22.6 |
| Caudal* |  |  | 17 |  |  |
| Innominate | 18 | 25 | 43 | 21.5 | 37.4 |
| Scapula | 30 | 23 | 53 | 26.5 | 46.1 |
| Humerus |  |  |  |  |  |
| Proximal epiphysis | 21 | 18 | 39 | 19.5 | 33.9 |
| Shaft | 56 | 53 | 109 | 54.5 | 94.8 |
| Distal epiphysis | 41 | 41 | 82 | 41.0 | 71.3 |
| Radius |  |  |  |  |  |
| Proximal epiphysis | 57 | 32 | 89 | 44.5 | 77.4 |
| Shaft | 69 | 46 | 115 | 57.5 | 100.0 |
| Distal epiphysis | 37 | 31 | 68 | 34.0 | 59.1 |
| Ulna** |  |  |  |  |  |
| Proximal epiphysis | 57 | 40 | 97 | 48.5 | 84.3 |
| Shaft | 67 | 43 | 110 | 55.0 | 95.7 |
| Distal epiphysis | 28 | 16 | 44 | 22.0 | 38.3 |
| Carpal | 43 | 32 | 75 | 4.7 | 8.2 |
| Metacarpal |  |  |  |  |  |
| Third metacarpal | 18 | 18 | 36 | 18.0 | 31.3 |
| Fourth metacarpal | 19 | 21 | 40 | 20.0 | 34.8 |
| Femur |  |  |  |  |  |
| Proximal epiphysis | 24 | 13 | 37 | 18.5 | 32.2 |
| Shaft | 40 | 42 | 82 | 41.0 | 71.3 |
| Distal epiphysis | 27 | 32 | 59 | 29.5 | 51.3 |
| Patella | 8 | 10 | 18 | 9.0 | 15.7 |
| Tibia |  |  |  |  |  |
| Proximal epiphysis | 36 | 16 | 52 | 26.0 | 45.2 |
| Shaft | 63 | 46 | 109 | 54.5 | 94.8 |
| Distal epiphysis | 45 | 37 | 82 | 41.0 | 71.3 |
| Fibula |  |  |  |  |  |
| Proximal epiphysis | 2 | 1 | 3 | 1.5 | 2.6 |
| Shaft | 11 | 6 | 18*** | 9.0 | 15.7 |
| Distal epiphysis | 11 | 6 | 17 | 8.5 | 14.8 |
| Tarsal |  |  |  |  |  |
| Calcaneus | 23 | 32 | 55 | 22.5 | 39.1 |
| Astragalus | 16 | 27 | 43 | 21.5 | 37.4 |
| Other tarsal | 21 | 19 | 40 | 5.0 | 8.7 |
| Metatarsal |  |  |  |  |  |
| Third metatarsal | 23 | 23 | 46 | 23.0 | 40.0 |
| Fourth metatarsal | 22 | 21 | 43 | 21.5 | 37.4 |
| Phalanx |  |  |  |  |  |
| First phalanx |  |  | 72 | 9.0 | 15.7 |
| Second phalanx |  |  | 50 | 6.3 | 11.0 |
| Third phalanx |  |  | 28 | 3.5 | 6.1 |

*MAU not calculated given variable representation of this element in complete skeletons.

**Includes specimens attached to and unattached from radii.

***Includes one unsided specimen.

The Haile 21A skeletal part profiles must be viewed with some caution given the site’s discovery in an active commercial quarry and its disjointed collection history—especially the initial, and poorly documented, exploration by commercial fossil collectors. While neither issue is unusual with paleontological work in Florida, either (or both) make it difficult to determine how well the curated assemblage accurately reflects what was originally deposited in antiquity. For several reasons, we think that the major long bones provide the most unbiased picture of skeletal part representation and, as such, indicate that more-or-less complete peccary carcasses were deposited in the Haile 21A sinkhole. These bones are first of all large enough to have been spotted and collected by fossil dealers at rates at least similar to those of the more meticulous museum excavators. Given that long bones typically do not fetch premium prices on the fossil market, they are also more likely to have been left behind and/or turned over to the museum. Indeed, of the 640 specimens in the curated Haile 21A peccary assemblage known to have been collected and donated by fossil dealers, over one third (235, or 36.7%) belong to one of the major long bones. By comparison, 178 (27.9%) of these are skull specimens, all of which are fragments and/or isolated teeth rather than complete, and more commercially valuable, specimens. Finally, due to their thick, durable cortical bone, the major long bones are less susceptible to the density-mediated attritional processes (e.g., sediment compaction, chemical attack, and carnivoran feeding) that often render other, less-dense elements like vertebrae analytically (i.e., made unidentifiable) or truly (i.e., destroyed fully) absent from an assemblage [2].

If we concentrate on the major long bones, single proportion permutation tests on MNE values reveal that only the frequencies of radii differ significantly from what would be expected under equal bilateral representation (Table S4). This pattern holds more generally for the other bilateral skeletal elements, with only the ulna deviating significantly from an equal left-right split. This indicates that the left and right sides of peccary carcasses were excavated at roughly equal rates from the Haile 21A sinkhole. Based on these considerations, we offer what is probably the most parsimonious explanation for the observed skeletal part profile: after the peccaries were deposited in or around the cavity as complete, or nearly complete, carcasses, they experienced some density-mediated destruction of less durable skeletal elements and/or skeletal element portions. After the site’s discovery, but prior to the museum’s excavations, fossil dealers selectively removed skeletal elements of high commercial value, including most of the well-preserved skulls. Controlled excavations then recovered a majority of the larger and/or skeletally identifiable remains, while a portion of the smaller and/or less identifiable remains were recovered through water screening of sediment samples. The resumption of mining operations after the museum ceased excavations in March of 1984 then destroyed all traces of the site and any of its remaining fossils.

Table S4. Results of permutation tests of equal bilateral representation for major long bones for *Platygonus vetus* from Haile 21A.

| Element | MNE Left | MNE Right | MNE Total | Proportion MNE Left | p-value* |
| --- | --- | --- | --- | --- | --- |
| Humerus | 56 | 53 | 109 | 0.51 | 0.70 |
| Radius | 69 | 46 | 115 | 0.60 | 0.03 |
| Femur | 40 | 42 | 82 | 0.49 | 0.90 |
| Tibia | 63 | 46 | 109 | 0.58 | 0.12 |
| Ulna | 67 | 43 | 110 | 0.61 | 0.03 |
| Innominate | 18 | 25 | 43 | 0.42 | 0.37 |
| Scapula | 30 | 23 | 53 | 0.57 | 0.41 |
| Carpal | 43 | 32 | 75 | 0.57 | 0.24 |
| Third metacarpal | 18 | 18 | 36 | 0.50 | 1.00 |
| Fourth metacarpal | 19 | 21 | 40 | 0.48 | 0.87 |
| Patella | 8 | 10 | 18 | 0.44 | 0.48 |
| Fibula | 11 | 6 | 17 | 0.65 | 0.35 |
| Tarsal | 60 | 78 | 138 | 0.44 | 0.15 |
| Third metatarsal | 23 | 23 | 46 | 0.50 | 1.00 |
| Fourth metatarsal | 22 | 21 | 43 | 0.51 | 0.76 |
| Total | 547 | 487 | 1034 | 0.52 | 0.22 |

*p-value indicates the two-tailed probability of observing the “Proportion MNE Left” in a bootstrapped (that is, randomly sampled with replacement) distribution of 5,000 proportions of size “MNE Total” when the proportional representation of left elements = 0.50.

**Bone damage**

*General patterns*

The Haile 21A faunal assemblage exhibits moderate levels of damage attributable to carnivores. About 8% of all non-skull bone specimens and about 16% of specimens from the major long bones (i.e., humerus, radio-ulna, femur, tibia) bear at least one tooth mark (= pit, score, and/or puncture) (Table S5). The vast majority of the long bones preserve no or very few individual tooth marks, and none preserve more than 10 individual tooth marks on their cortical surfaces (Table S6). None of the major long bones show crenulated edges that would indicate persistent gnawing. Only about 50% of the major long bones are complete (Table S7), although cylinders (i.e., long bones that preserve their complete diaphyseal circumference but lack both the proximal and distal epiphyses) are rare (n = 49; 7.6% of long bone NISP). For those elements where a subaerial weathering stage could be reliably assigned, a majority fall into Stage 0 (Table S8).

Table S5. Tooth mark frequencies by skeletal element for *Platygonus vetus* from Haile 21A.

| **Element** | **NISP** | **NISP TM** | **%NISP TM** |
| --- | --- | --- | --- |
| Rib | 191 | 8 | 4.2 |
| Vertebra | 320 | 23 | 7.2 |
| Innominate | 55 | 5 | 9.1 |
| Scapula | 57 | 5 | 8.8 |
| Humerus | 122 | 21 | 17.2 |
| Radio-ulna | 127 | 14 | 11.0 |
| Carpal | 75 | 1 | 1.3 |
| Metacarpal | 76 | 3 | 3.9 |
| Femur | 97 | 28 | 28.9 |
| Tibia | 120 | 14 | 11.7 |
| Fibula | 21 | 0 | 0.0 |
| Tarsal | 138 | 6 | 4.3 |
| Metatarsal | 59 | 2 | 3.4 |
| Phalanx | 140 | 2 | 1.4 |
| Metapodial | 40 | 1 | 2.5 |
| Patella | 18 | 0 | 0.0 |
| Sesamoid | 8 | 0 | 0.0 |
| Total | 1694 | 133 | 7.9 |

Table S6. Number of individual tooth marks by long bone type for *Platygonus vetus* from Haile 21A.

| **Element** | **NISP** | **No TM (%)** | **1 TM (%)** | **2-5 TM (%)** | **6-10 TM (%)** | **>10 TM (%)** |
| --- | --- | --- | --- | --- | --- | --- |
| Humerus | 122 | 100 (82.0%) | 15 (12.3%) | 6 (4.9%) | 0 (0.0%) | 0 (0.0%) |
| Radio-ulna | 127 | 113 (89.0%) | 11 (8.7%) | 3 (2.4%) | 0 (0.0%) | 0 (0.0%) |
| Metacarpal | 76 | 73 (96.1%) | 2 (2.6%) | 1 (1.3%) | 0 (0.0%) | 0 (0.0%) |
| Femur | 97 | 69 (71.1%) | 15 (15.5%) | 6 (6.2%) | 7 (7.2.%) | 0 (0.0%) |
| Tibia | 120 | 106 (88.3%) | 5 (4.2%) | 9 (7.5%) | 0 (0.0%) | 0 (0.0%) |
| Metatarsal | 59 | 57 (96.6%) | 1 (1.7%) | 1 (1.7%) | 0 (0.0%) | 0 (0.0%) |
| Total | 631 | 448 (71.0%) | 1 (1.7%) | 26 (4.1%) | 7 (1.1%) | 0 (0.0%) |

Table S7. Frequency of complete bones among major long bones for *Platygonus vetus* from Haile 21A.

| **Element** | **MNE** | **MNE complete*** | **%MNE complete** |
| --- | --- | --- | --- |
| Humerus | 109 | 31 | 28.4 |
| Radio-ulna | 115 | 44 | 38.3 |
| Femur | 82 | 34 | 41.5 |
| Tibia | 109 | 42 | 38.5 |
| Total | 415 | 151 | 36.4 |

**Defined as long bones with unbreached marrow cavities.

Table S8. Weathering stage frequencies by skeletal element for *Platygonus vetus* from Haile 21A.

| **Element** | **NISP** | **Stage 0 (%)** | **Stage 1 (%)** | **Stage 2 (%)** | **Not recorded (%)** |
| --- | --- | --- | --- | --- | --- |
| Scapula | 57 | 23 (40.4%) | 12 (21.1%) | 0 (0.0%) | 22 (38.6%) |
| Humerus | 122 | 24 (19.7%) | 14 (11.5%) | 1 (0.8%) | 83 (68.0%) |
| Radio-ulna | 127 | 34 (26.8%) | 27 (21.3%) | 2 (1.6%) | 64 (50.4%) |
| Innominate | 55 | 21 (38.2%) | 5 (9.1%) | 0 (0.0%) | 29 (52.7%) |
| Femur | 97 | 52 (53.6%) | 7 (7.2%) | 0 (0.0%) | 38 (39.2%) |
| Tibia | 120 | 36 (30.0%) | 11 (9.2%) | 0 (0.0%) | 73 (60.8%) |
| Total | 578 | 190 (32.9%) | 76 (13.1%) | 3 (0.5%) | 309 (53.5%) |

*Element-by-element descriptions of damage*

Scapulae

Not a single scapula is complete. The thin blades of scapulae often do not preserve features that permit the ready discernment of the timing of breakage. The nutritive phase breakage that can be confidently identified, however, frequently co-occurs with tooth marks and involves the removal of the proximal end of the blade (Figure S1). Two scapular necks preserve tooth pits (e.g., Figure S2) that echo the pattern documented among tiger-consumed carcasses. The size of these tooth pits compares favorably to that of the *Xenosmilus* incisors and canine—the I_3_ is the best candidate given its serrated distal carena (Figure S3). Another specimen shows a peculiar double-arched breakage outline on the distal blade that fits very well the size and shape of the *Xenosmilus* upper incisors (Figure S4). This indicates that feeding proceeded in some cases from the proximal to the distal end and that the anterior dentition was used to both strip flesh and break bone.


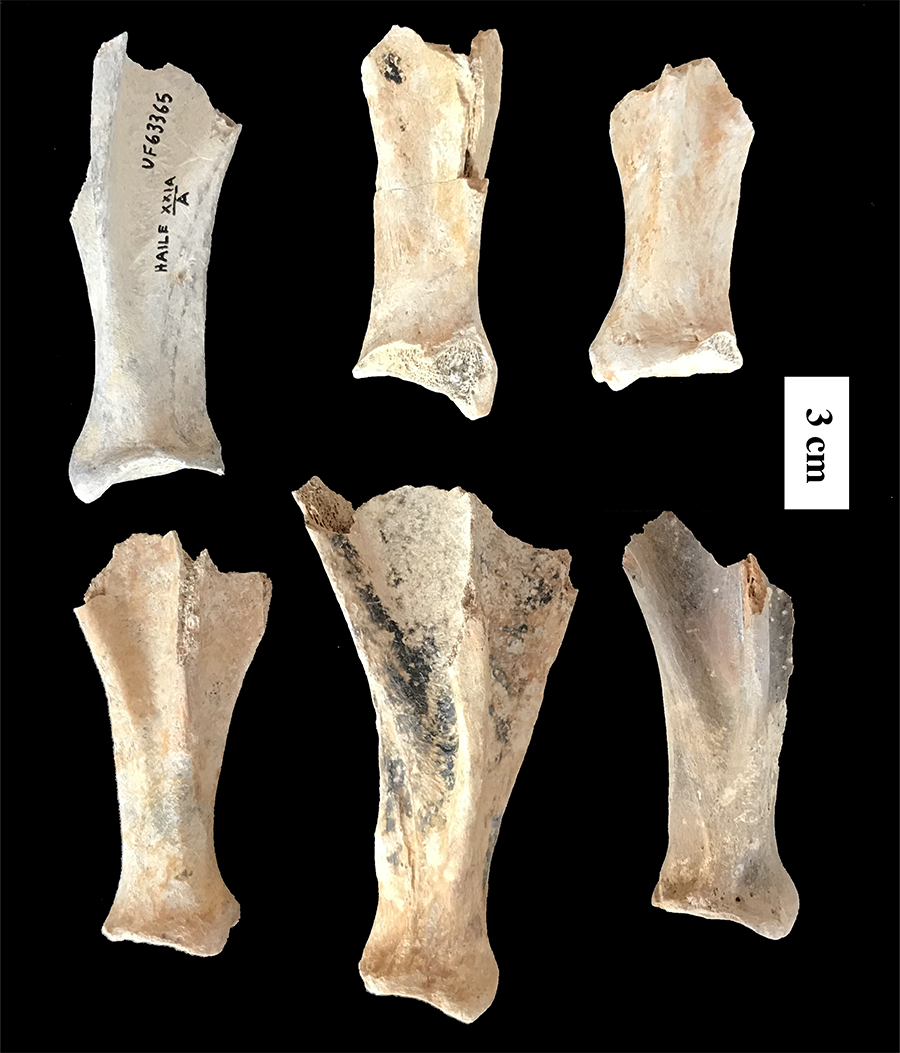


Fig S1. Lateral views of scapulae showing typical breakage pattern.


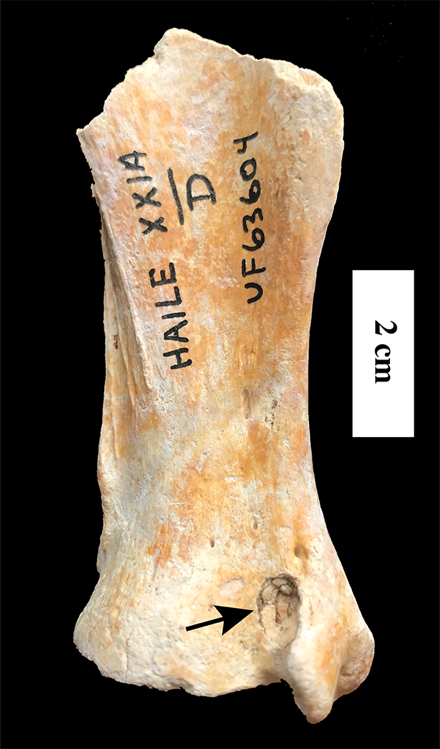


Fig S2. Medial view of a distal left scapula with a large tooth pit (black arrow) adjacent to the coronoid process. Compare the location of the tooth pit to that documented in Parkinson et al. [3: 132].


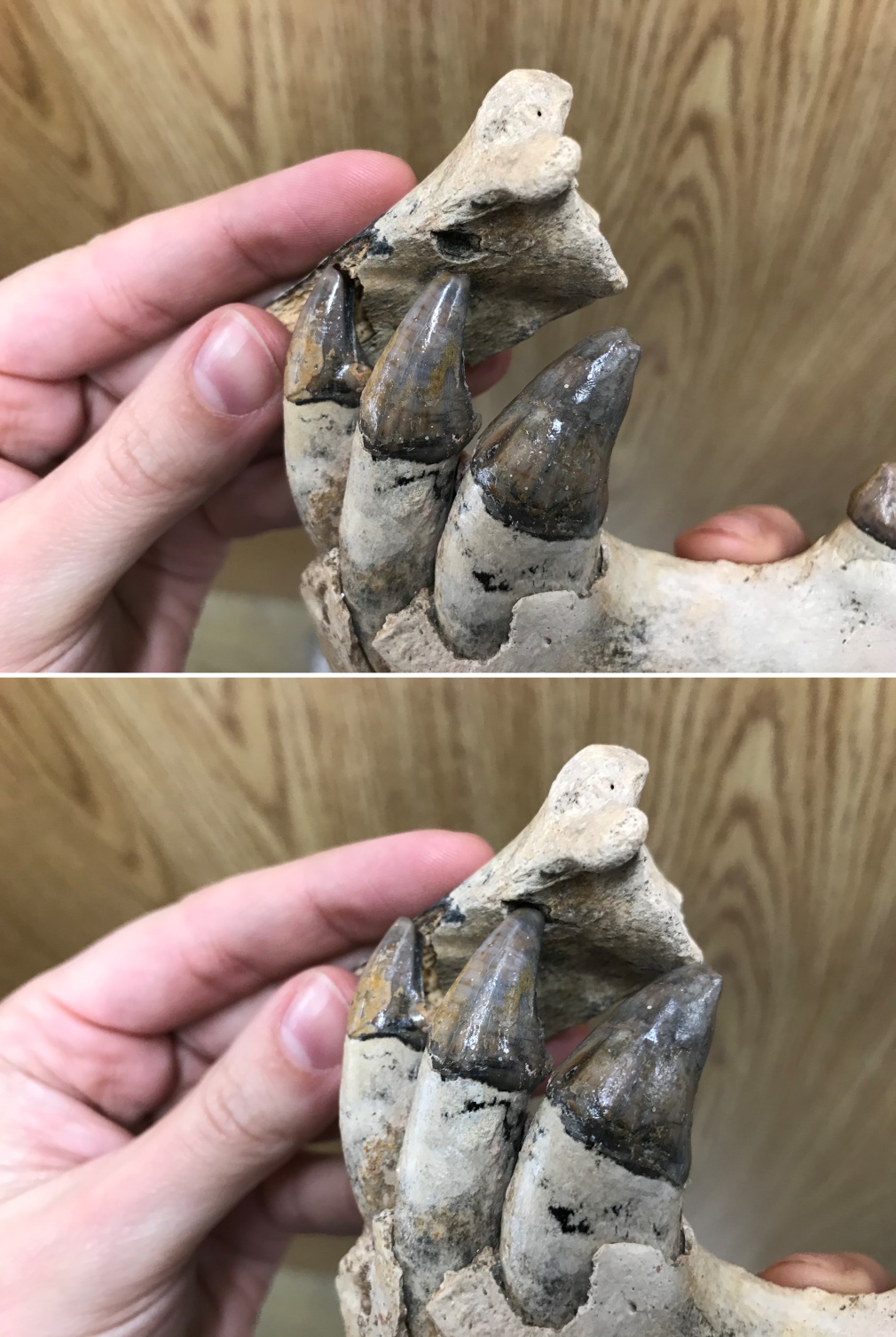


Fig S3. Tentative fitting of the lower incisor of Xenosmilus within a tooth pit on the medial aspect of a distal scapula.


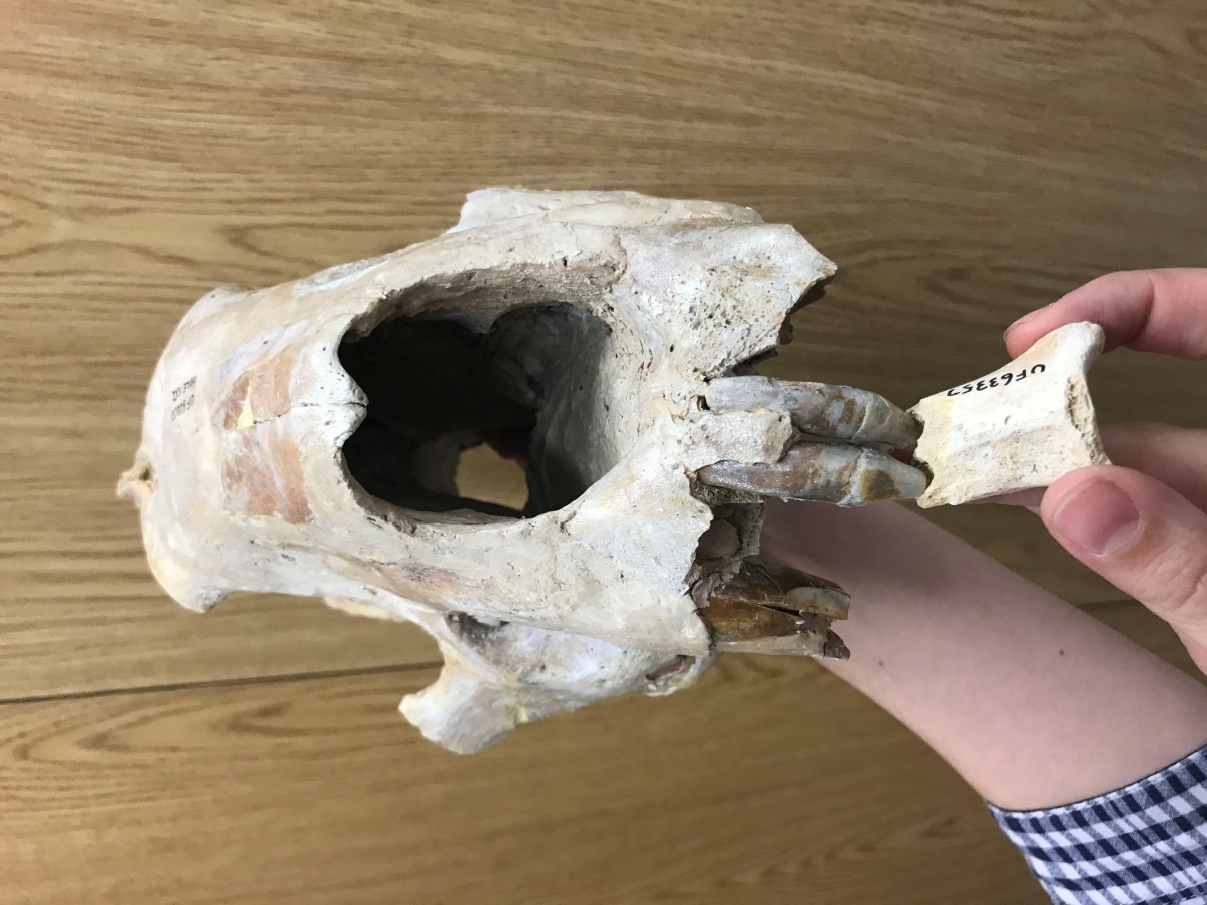


Fig S4. Cranium of Xenosmilus with the two central incisors oriented to the double-arched shape of the broken edge of a scapular blade.

Humeri

Nearly one-third (28%) of the Haile 21A humeri as estimated by MNE are complete or nearly so and about one fourth (24.7%) show clear evidence for nutritive phase breakage (Figure S5). A wide range of carnivoran feeding traces, from isolated tooth marks and minor furrowing to extensive epiphyseal and even diaphyseal destruction, are preserved on these skeletal elements (Figure S6). Most specimens bear only a single tooth mark, and the maximum number observed on a single specimen was three. This suggests that sustained gnawing, which typically produces many overlapping tooth marks, was rare. The preservation of humeral portions is clearly density-mediated: distal epiphyses (n = 82), again as measured by MNE, far outnumber proximal epiphyses (n = 39). Most of the carnivoran damage consists of light furrowing. On the proximal end, furrowing is located on the tubercles and just under the lateral side of the humeral head and is accompanied by furrowing and, albeit rarely, tooth-marking, on the deltoid crest and the tricipital line (Figures S7-S10). These latter features are near the insertions for the brachialis and triceps brachialis muscles. This repeated modification of the lateral aspect of the proximal humerus is documented among modern lions (Figure S11). Furrowing and tooth-marking on the distal epiphyses occur frequently on the caudal and medial aspects of the medial epicondyle and trochlea (Figure S12 and S13), which mirrors observations from lion-consumed carcasses (Figure S14). Hyenids and canids, on the other hand, regularly modify both the medial and, in the case of the former taxon, especially the lateral aspects of the distal humerus [4,5]. It appears that the tooth marks on the medial aspect of distal humeri were created by a caudal (where the margin of the tooth mark is rounded) to cranial (where the mark opens up) motion, which is again seen on carcasses consumed by modern lions (Figure S14). The size of many tooth marks fits well with that of the *Xenosmilus* lower canine (Figure S15).


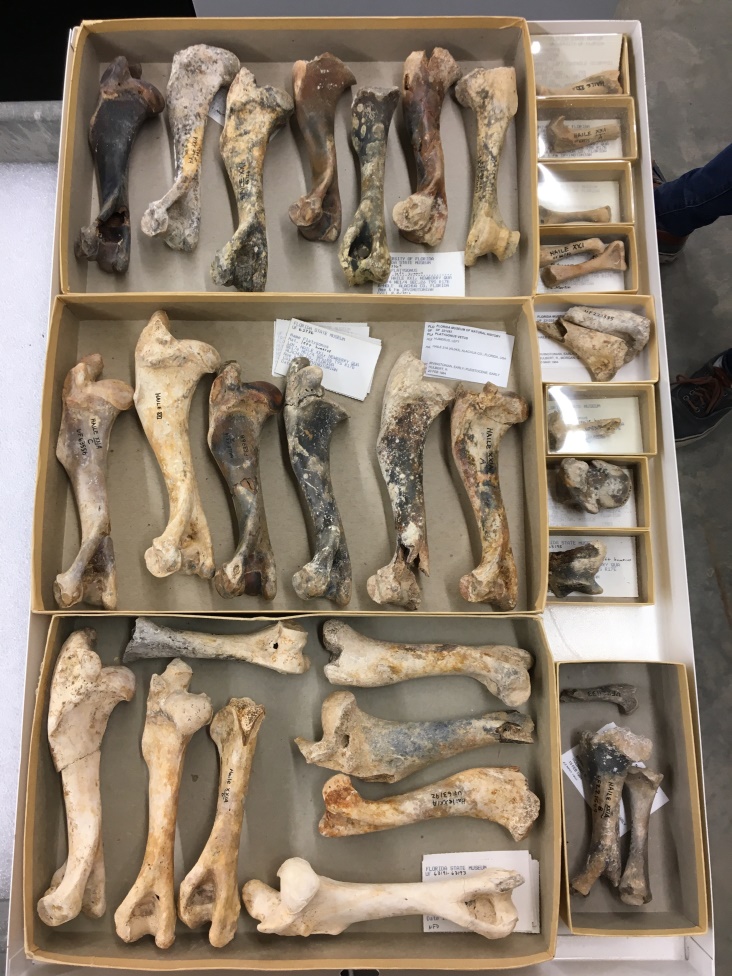


Fig S5. A sample of humerus specimens showing typical patterns of preservation.


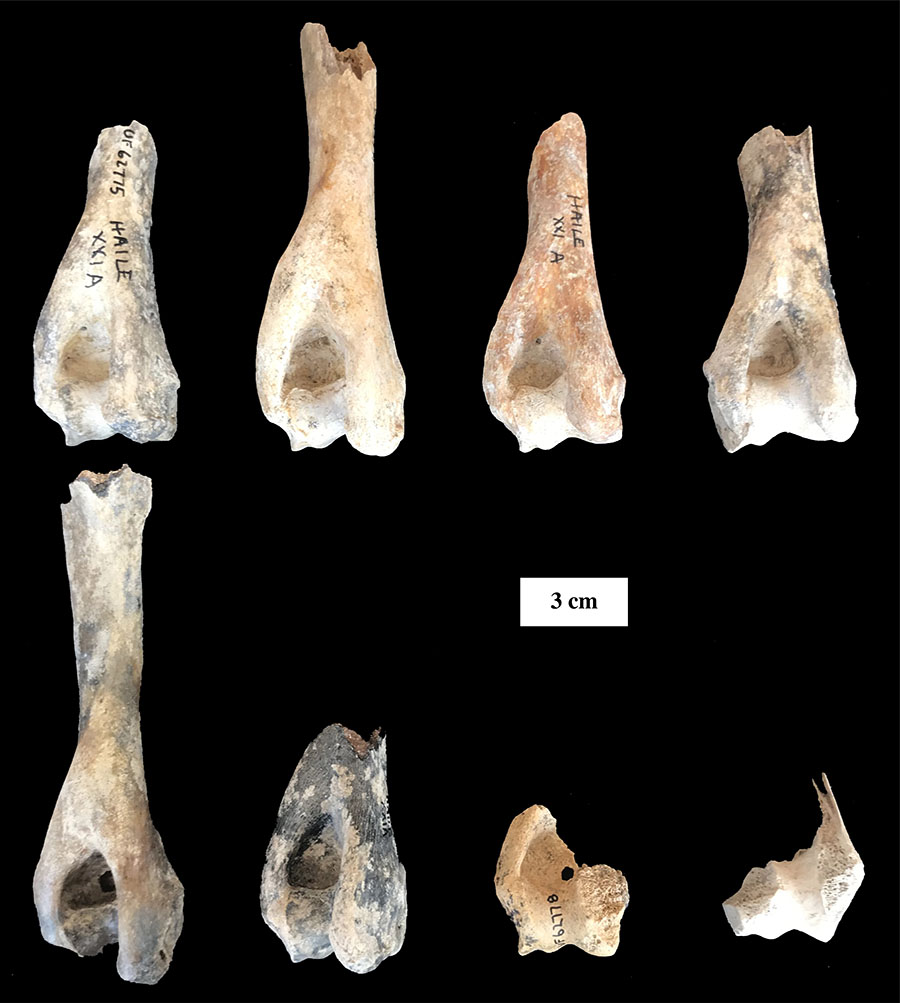


Fig S6. Caudal view of humerus specimens exhibiting various levels of destruction of the epiphysis and shaft.


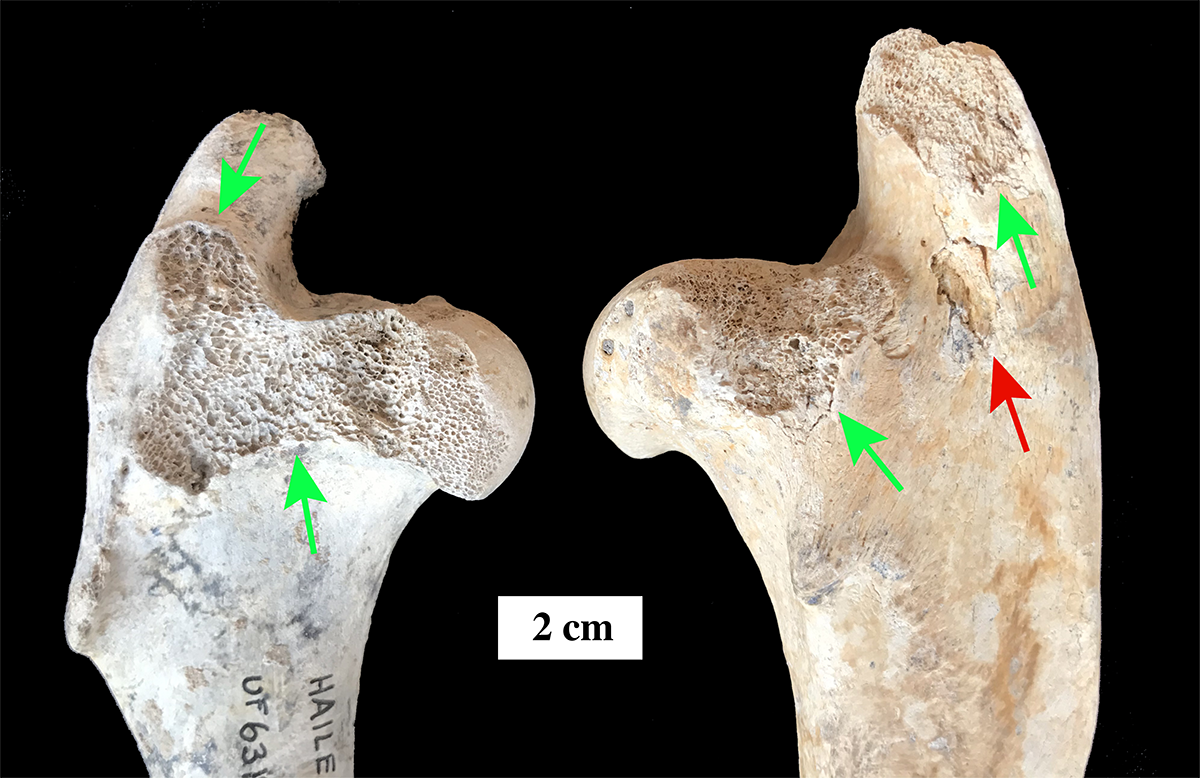


Fig S7. Lateral views of humeri showing furrowing (green arrows) and an elongated tooth pit (red arrow).


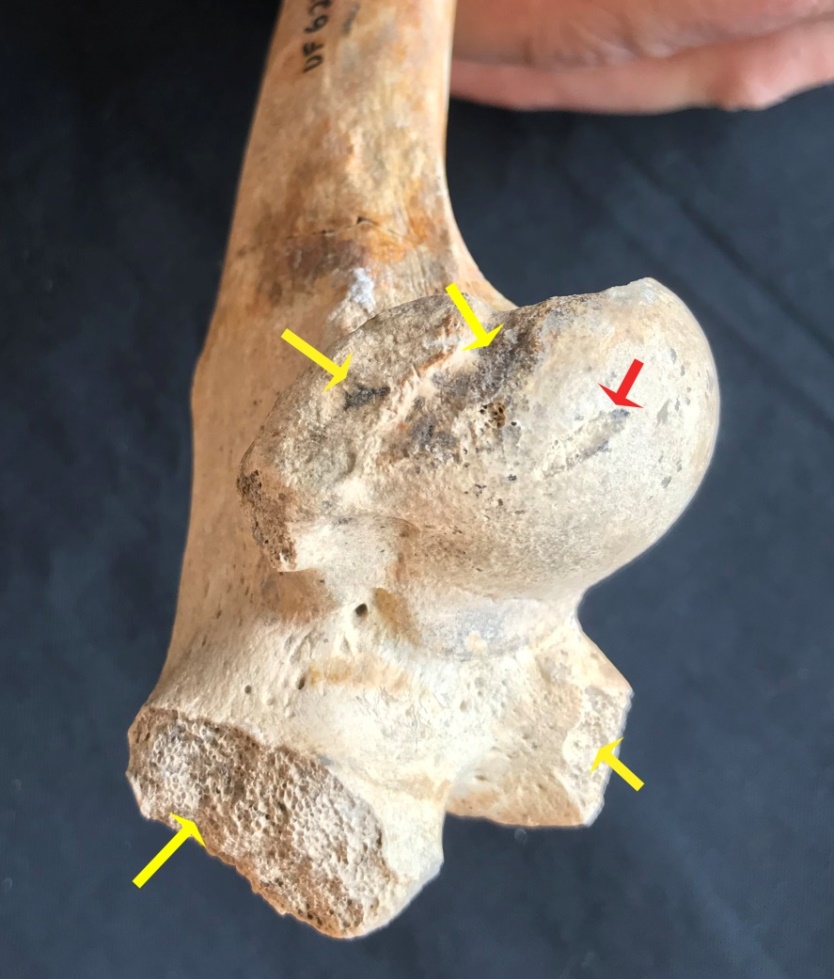


*Fig S8. Example of proximal humerus with mild furrowing on the tubercles (yellow arrows) and a tooth score (red arrow) on the articular surface.*

*
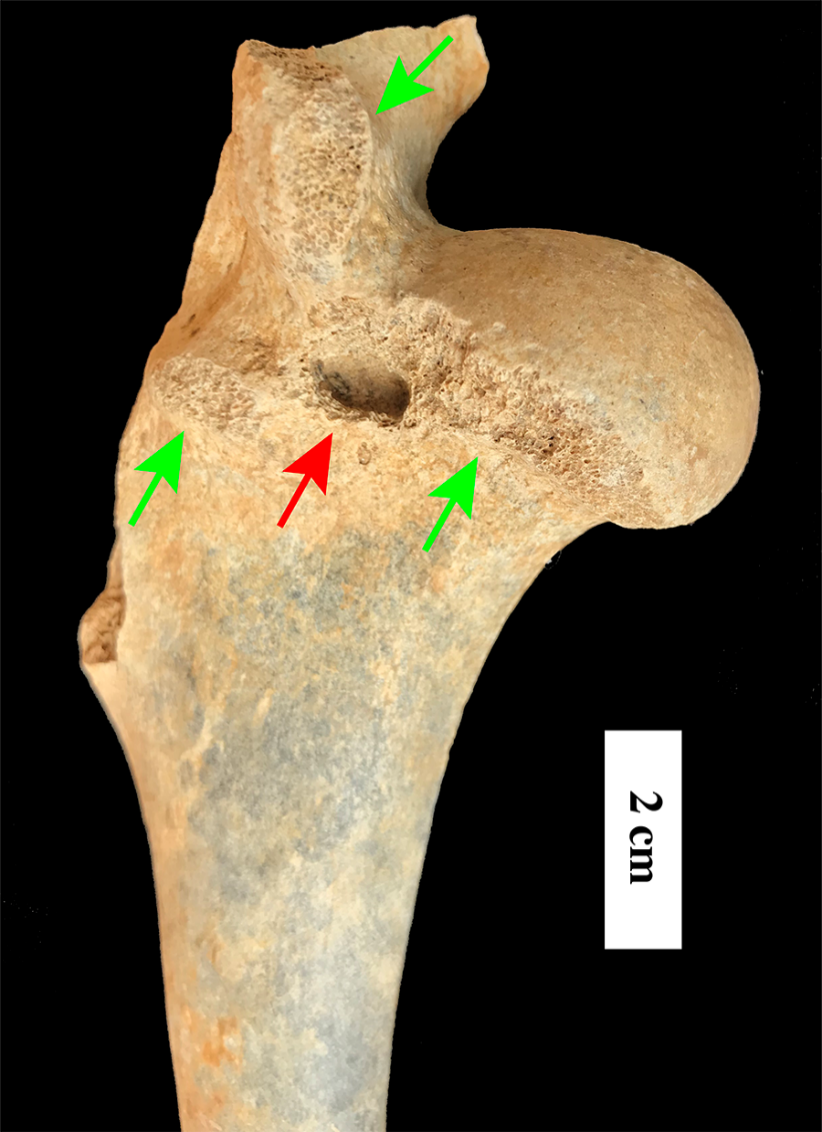
*

Fig S9. Lateral view of a humerus showing mild furrowing (green arrows) and tooth puncture (green arrow) near the tuberosity, deltoid crest, and head.


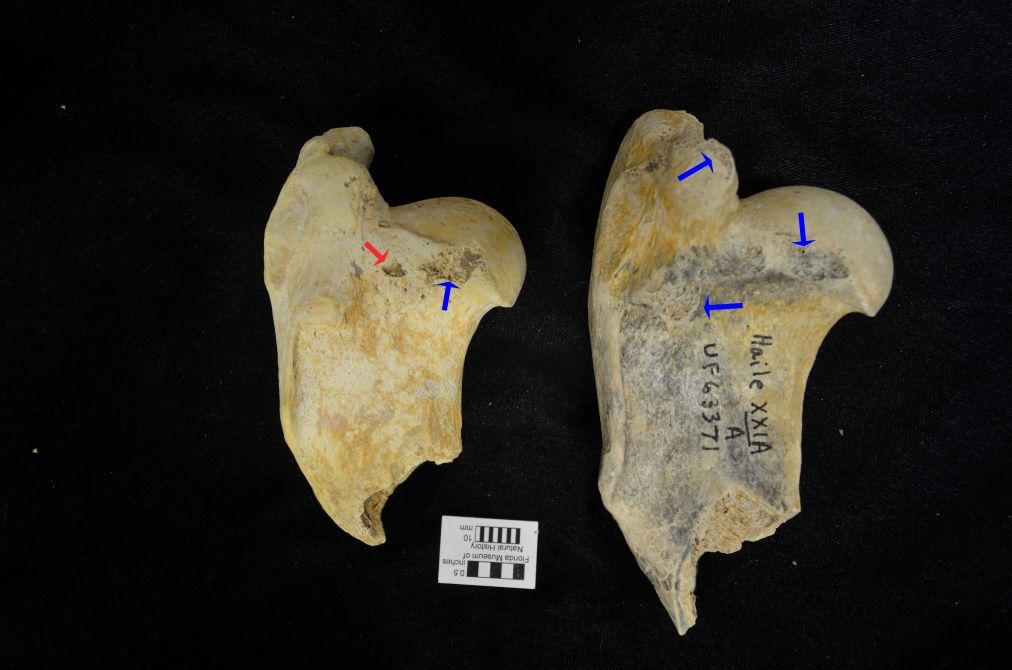


*Fig S10. Lateral views proximal humeri displaying nutritive phase breakage, furrowing (blue arrows), and tooth marking (red arrow). Note that most of the proximal epiphysis is otherwise unmodified.*


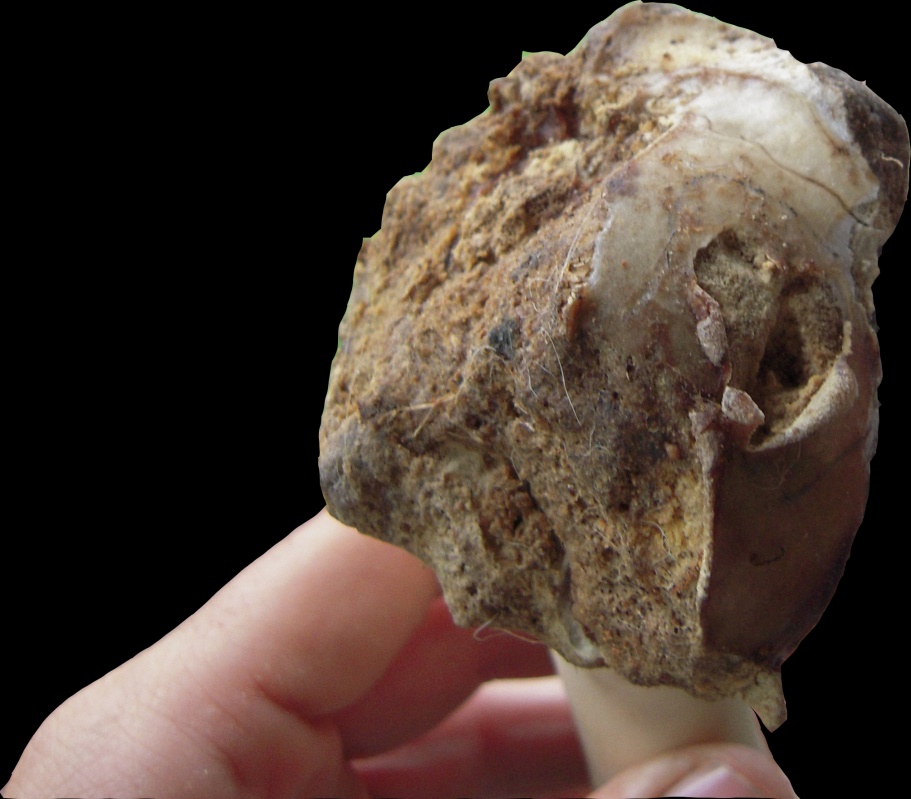


*Fig S11. Proximal humerus of a lion-consumed wildebeest (Gidna et al. 2014) with advanced modification of the lateral side.*


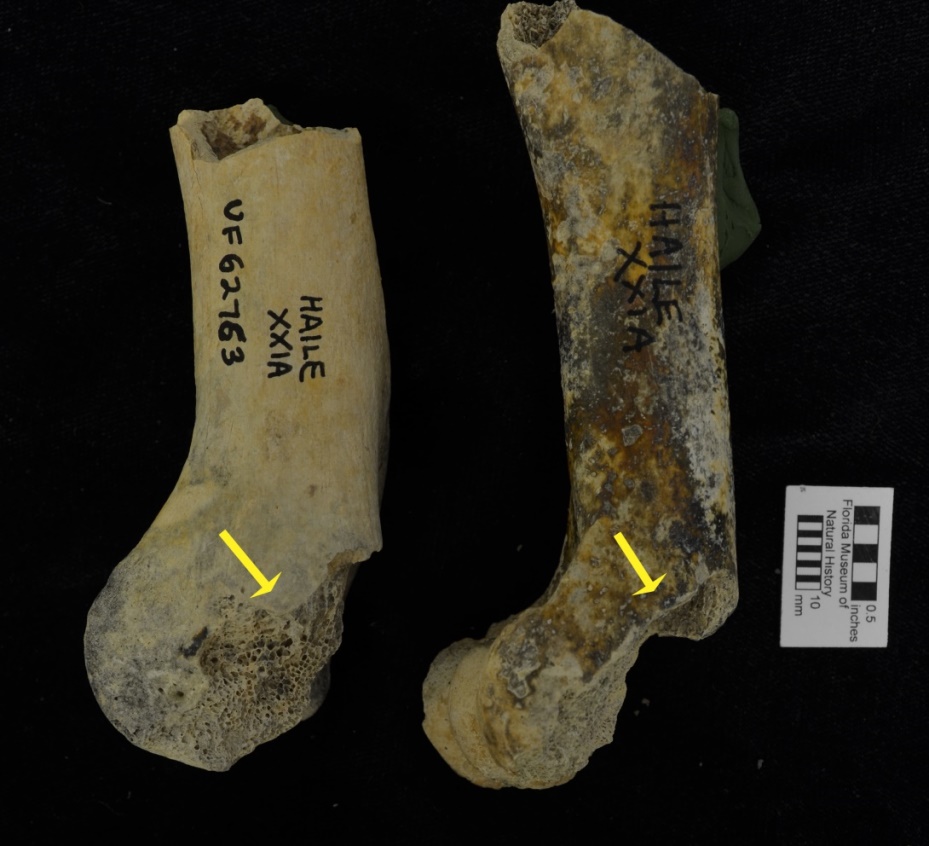


*Fig S12. Medial views of distal humeri showing furrowing on the postero-medial aspect of the epicondyle with a corresponding lack of modification on the lateral aspect. This pattern is more typical of large felids than it is of durophagus carnivorans.*


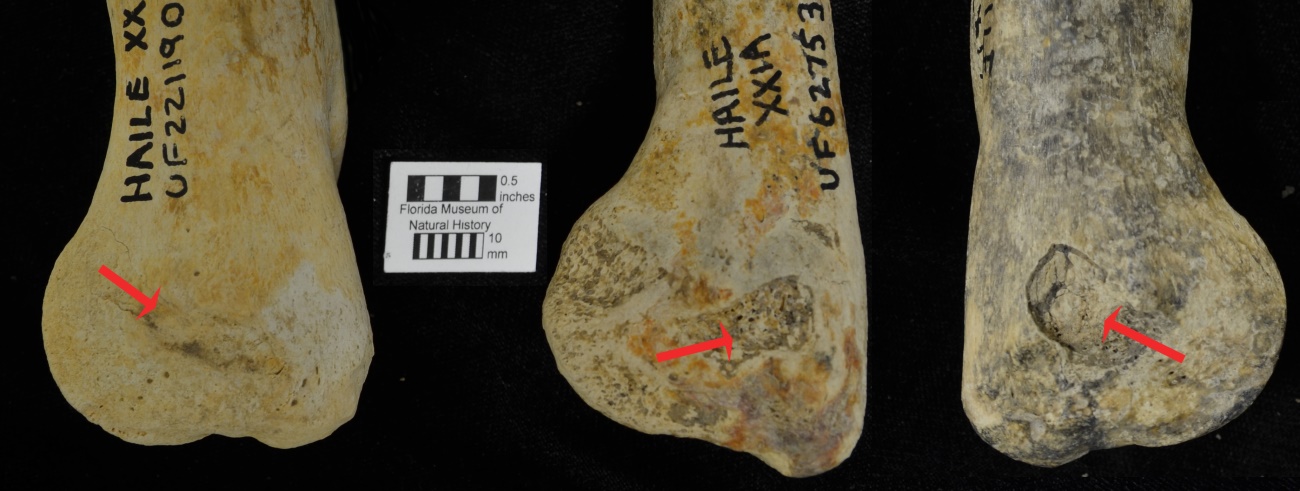


*Fig S13. Medial views of distal humeri showing tooth scores (red arrows, left and center) and a tooth pit (red arrow, right) on the trochlea and epicondyle in the absence of associated furrowing.*


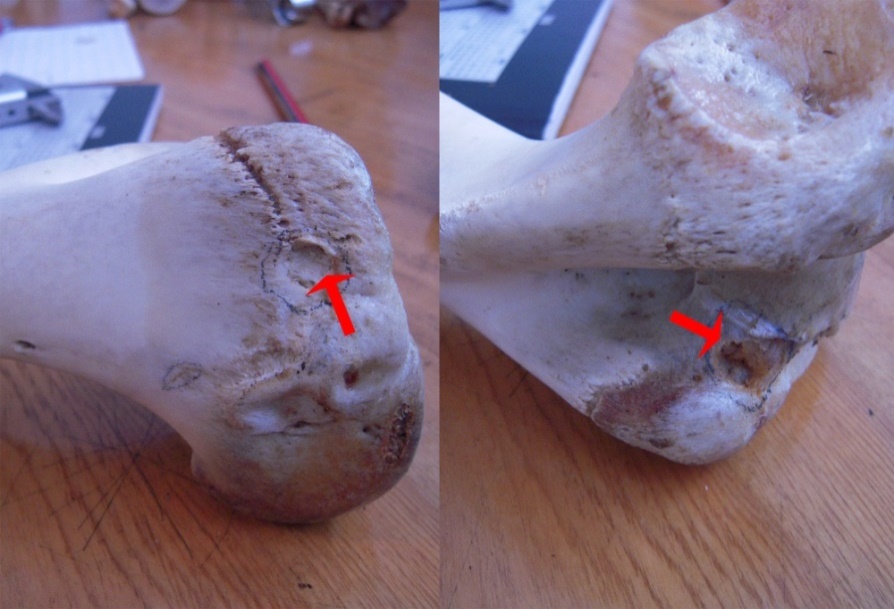


*Fig S14. Views of the distal humerus of a lion-consumed African buffalo (*Syncerus caffer*) (Gidna et al. 2014). Note the tooth-marking on the postero-medial (left) and postero-lateral (right) aspects of the medial epicondyle.*


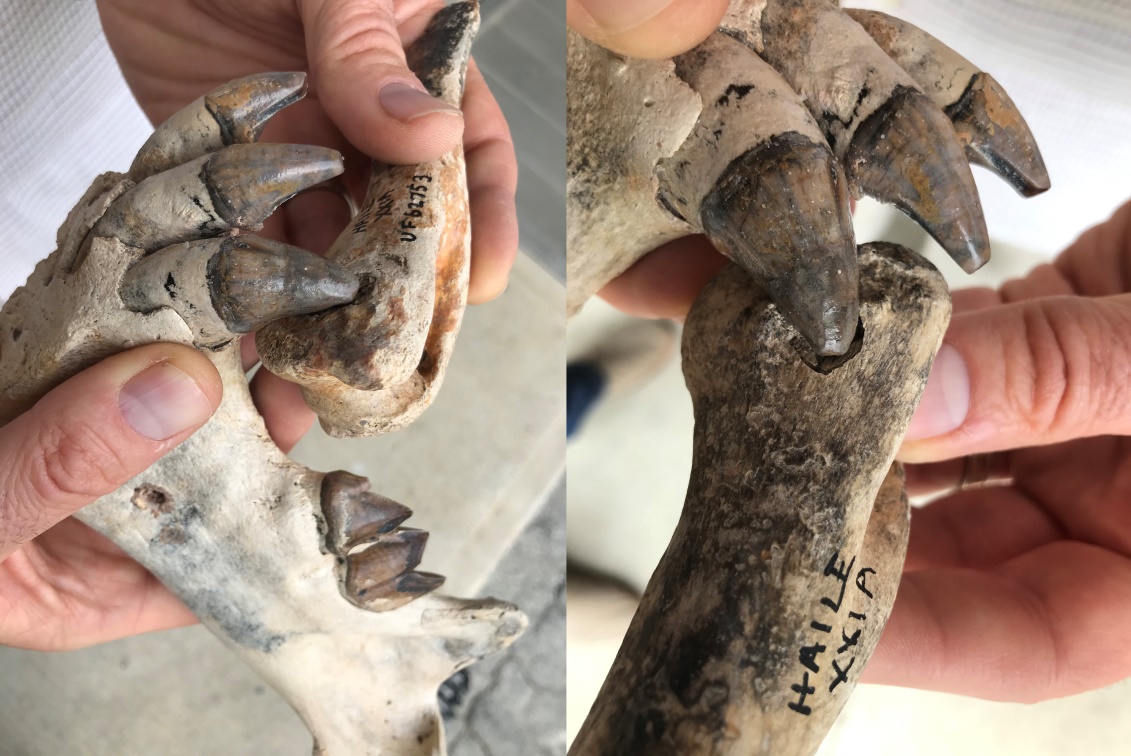


*Fig S15. Fitting of the* Xenosmilus *lower canine into tooth pits on the distal humerus pictured in the center of Figure S13 (left) and the distal humerus pictured on the right of Figure S13 (right).*

Radii and ulnae

In contrast to humeri, the Haile 21A radii exhibit very little damage from carnivoran feeding. As measured by MNE, only 20% of the radii show nutritive phase breakage, most of which occurs on the distal end. Otherwise, many elements are complete. We did note a recurrent pattern of breakage on the lateral aspect of several proximal radii (Figure S16). A handful of isolated ulna specimens refit onto these proximal breaks, however, and the breakage planes themselves show a different coloration than the adjacent cortical surfaces. It is therefore unclear whether this reflects diagenetic modification of ancient nutritive phase breaks or excavation/curation breakage. Given this ambiguity—and the fact that none of the specimens preserve tooth marks on or near these fractures—we do not consider these breaks to be the result of carnivoran feeding. Furrowing on radii is rare and occurs exclusively on the distal epiphysis and in association with a few tooth marks. The one measurable tooth pit, which occurs on a metaphysis, is consistent with a large carnivoran. The Haile 21A ulnae display regular furrowing on, and partial or full deletion of, the olecranon process (Figure S17). Specimens with tooth marks but little or no furrowing occur as well, however, which indicates that breaking through and consuming the cancellous bone of the proximal ulna was not always an objective of feeding (Figure S18). This is especially evident on juvenile ulnae, several of which are tooth-marked without deletion or intense furrowing of the olecranon (Figure S19). The high frequency of furrowed proximal ulnae and largely unmodified radii is typical of modern big cats [3,6,7].


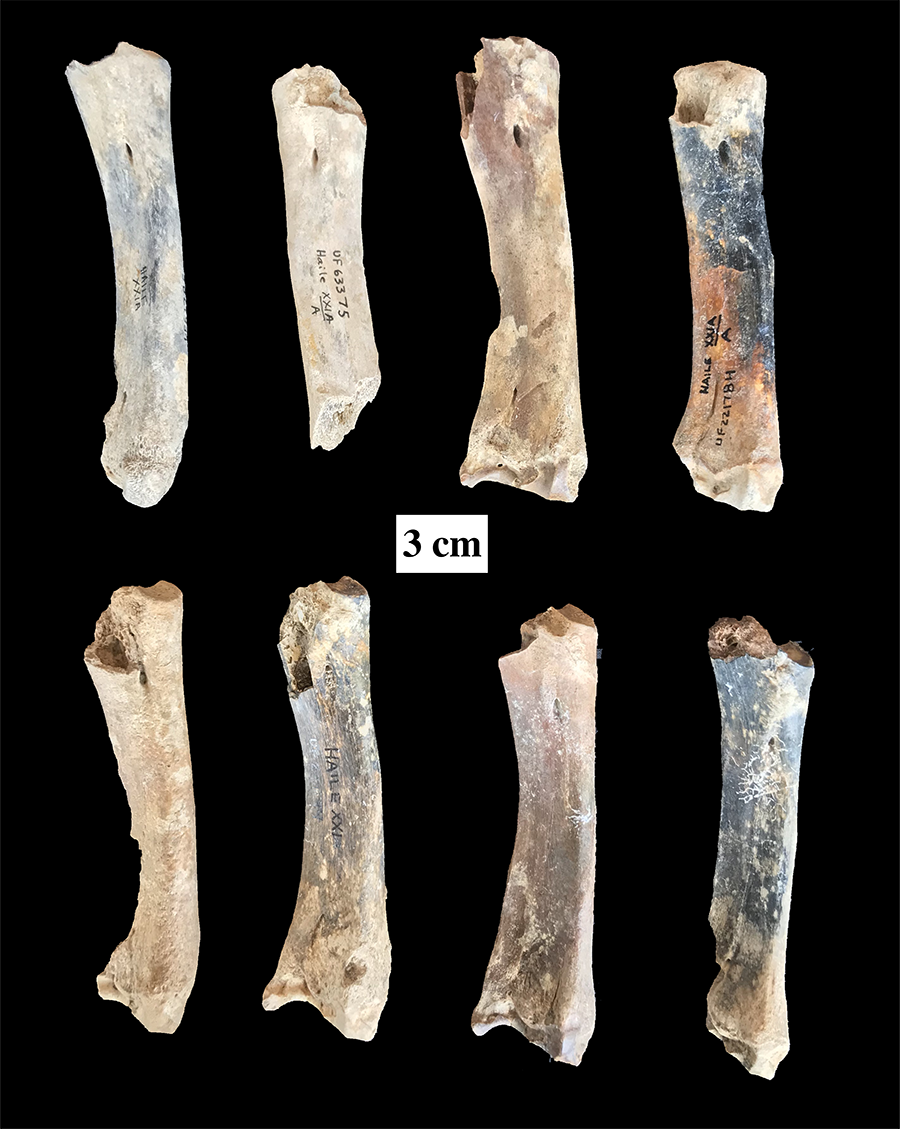


Fig S16. Postero-medial views of radio-ulnae showing patterned breakage near the proximal epiphysis


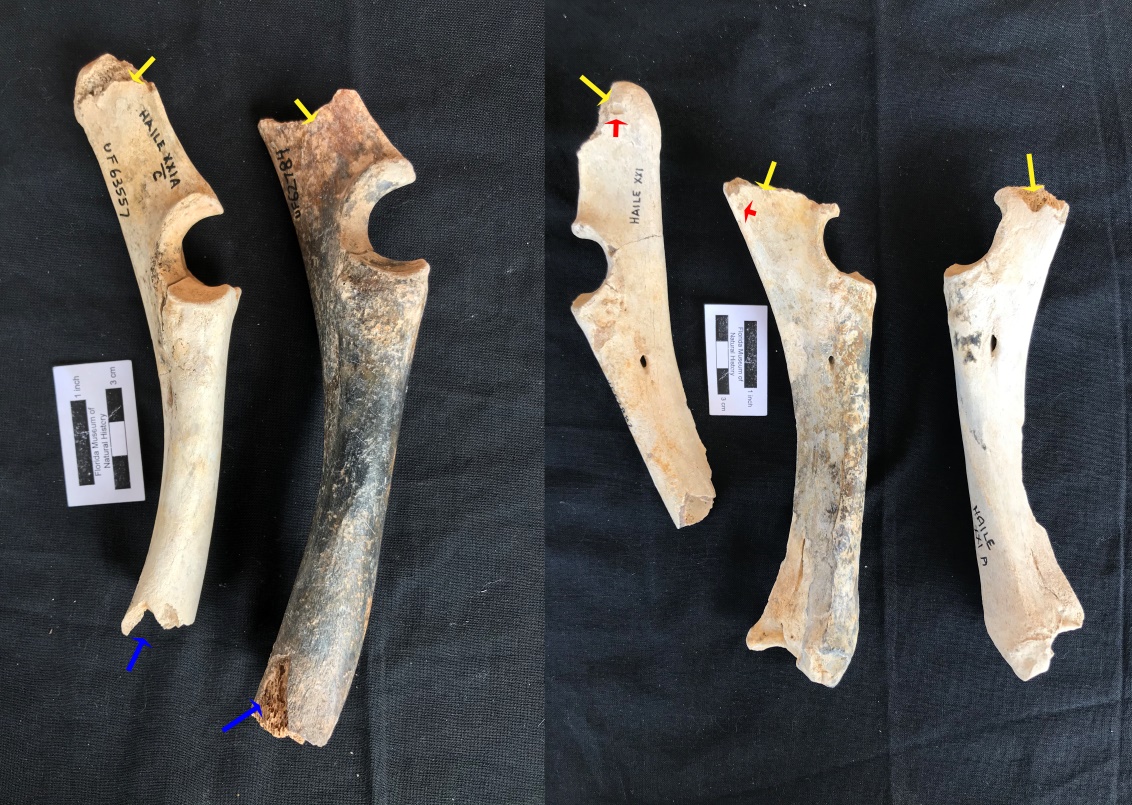


*Fig S17. Radio-ulnae showing various degrees of furrowing (yellow arrows), tooth-marking, and deletion of the distal end (blue arrows).*


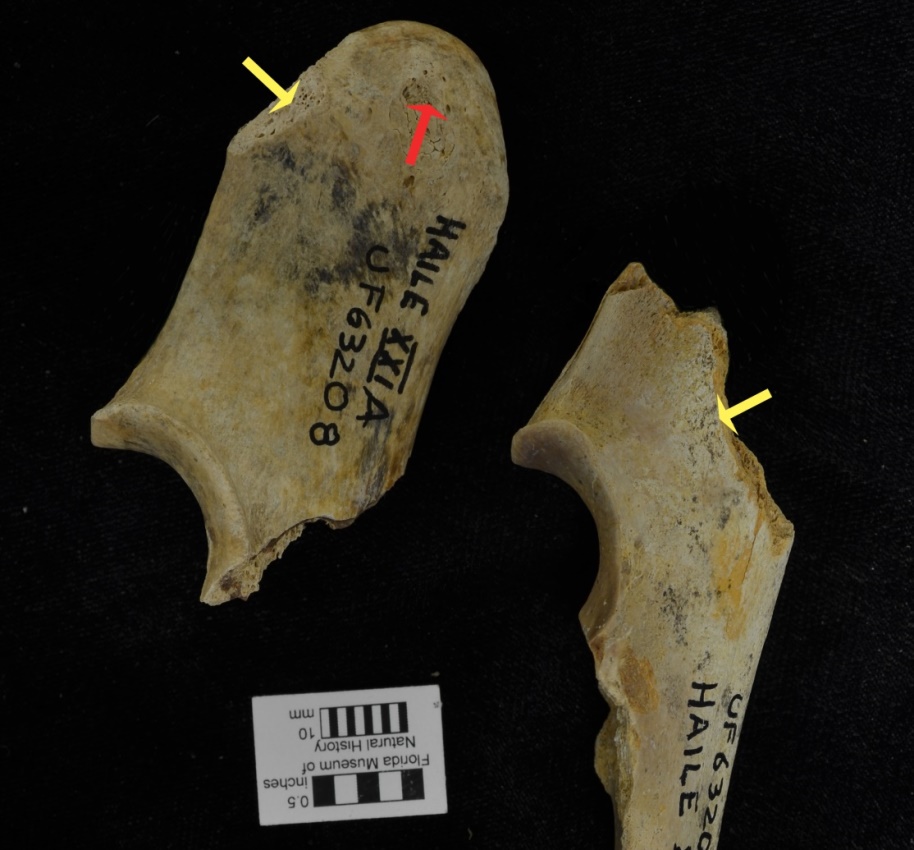


*Fig S18. Proximal ulnae showing intense (right) and mild (left) furrowing (yellow arrows) of the olecranon process. The presence of a large tooth pit (red arrow) associated with moderate crushing of the cortical layer into the cancellous bone indicates that the agent responsible is not a durophagous carnivoran.*


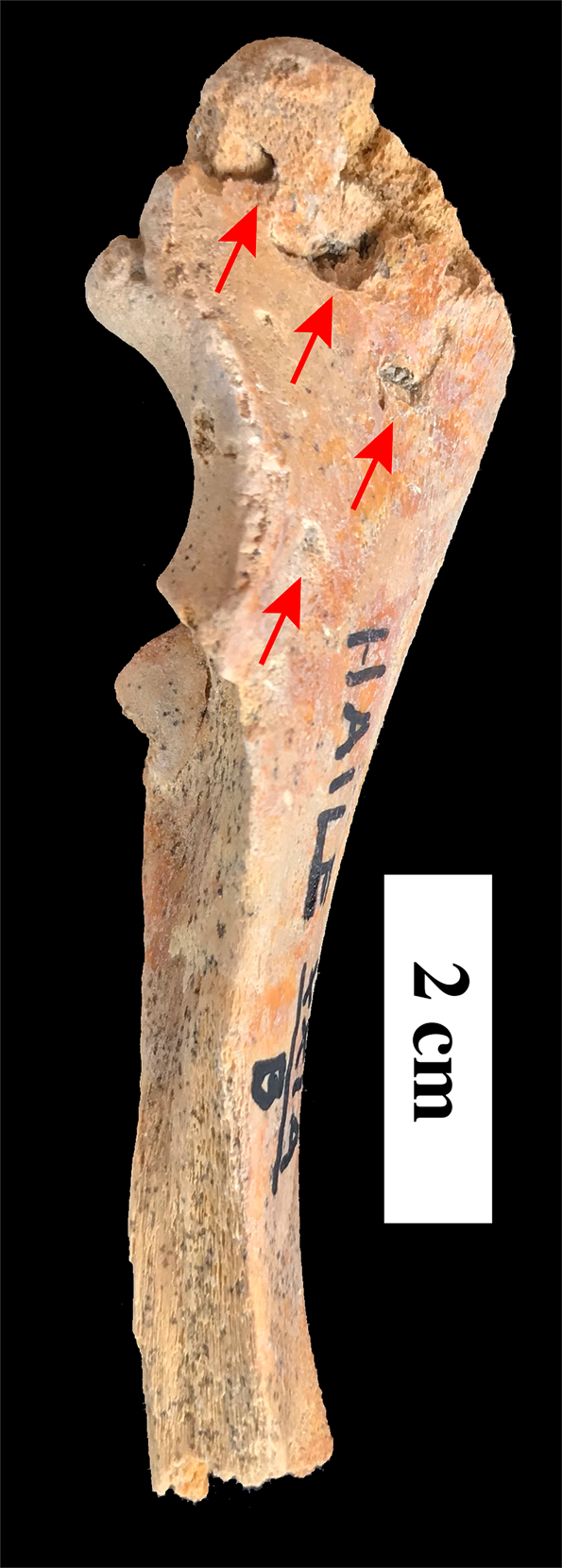


Fig S19. Medial view of a young juvenile ulna bearing several tooth marks (red arrows) with a virtual absence of furrowing.

Innominates

Several specimens show clear evidence of nutritive phase breakage in the form of moderate destruction of their iliac and ischial portions (Figure S20). On other specimens, the acetabulum is the only surviving portion, although discerning nutritive from non-nutritive phase breakage is difficult in these cases (Figure S21). Illiac blades in particular are largely absent, but one preserved portion does show a large tooth pit associated with light furrowing (Figure S22).


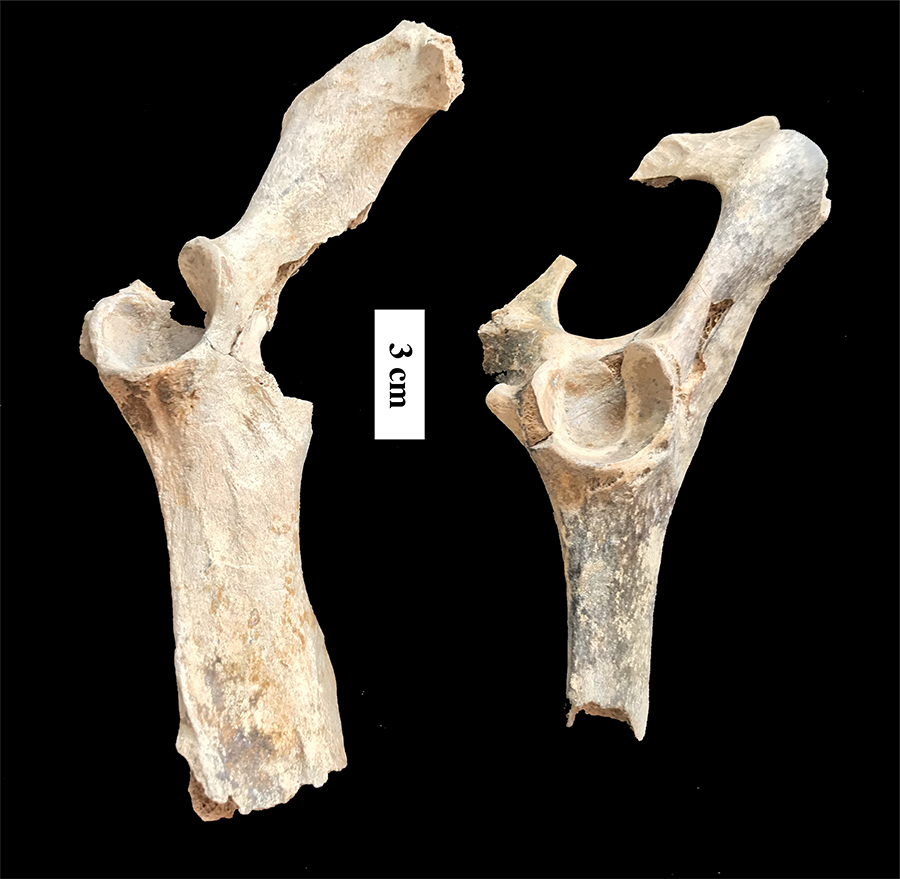


Fig S20. Examples of innominates showing partial destruction of the illium and ischium.


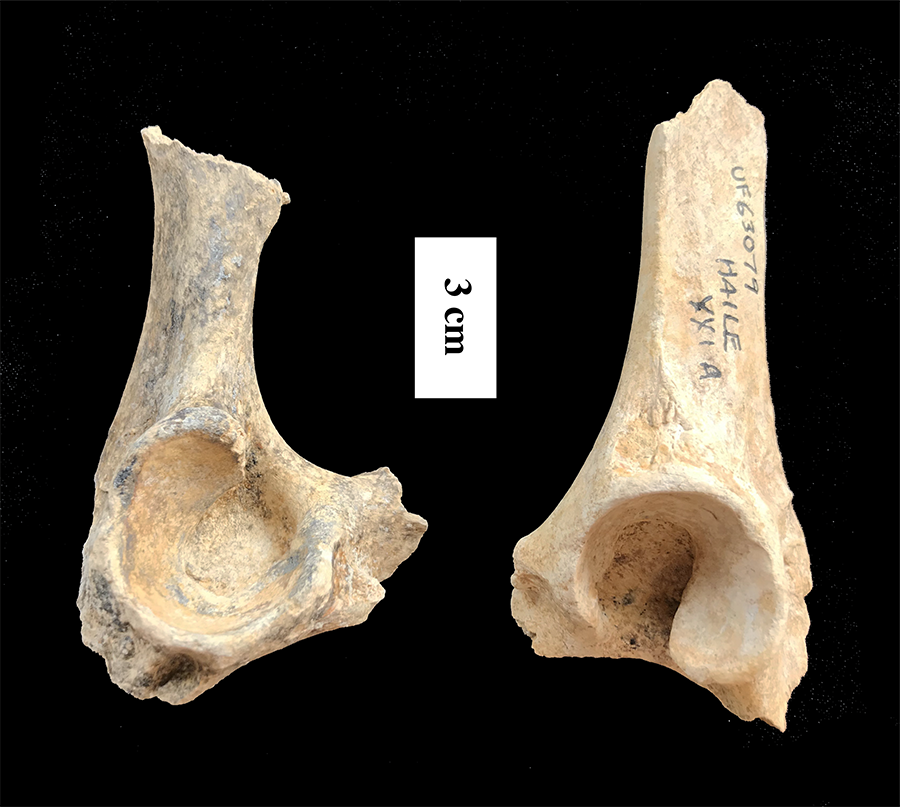


Fig S21. Examples of innominates showing more intense destruction of the illium, ischium, and pubis.


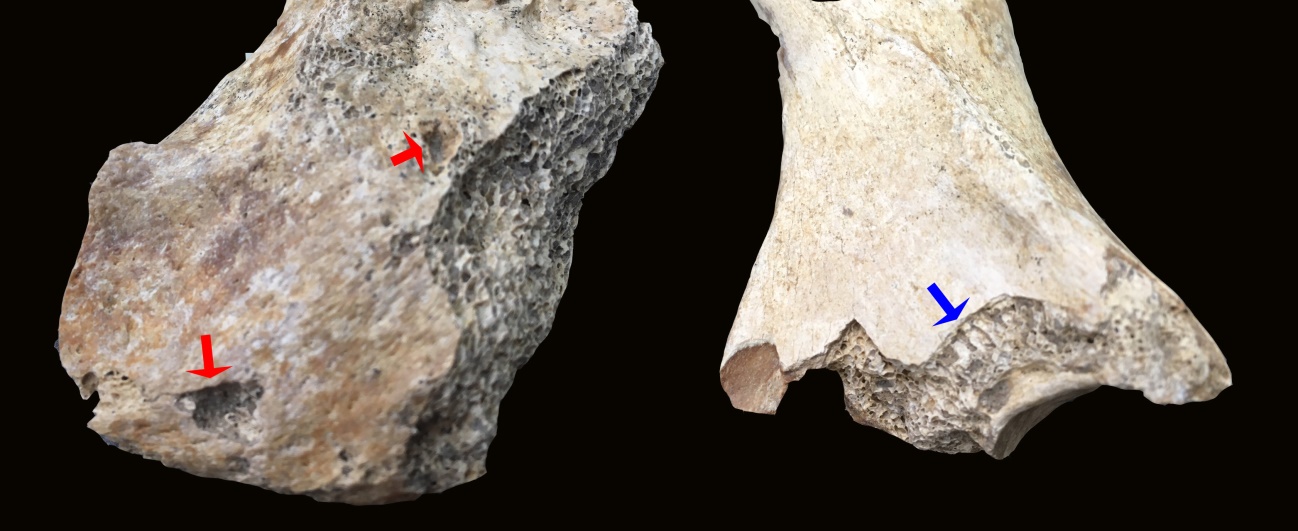


*Fig S22. Medial views of the iliac blade (left) and acetabulum (right) of an innominate showing mild furrowing of the iliac blade, two triangular-shaped tooth pits (red arrows), and furrowing around the acetabulum (blue arrow).*

Femora

Complete bones constitute over one third (41.5%) of the estimated femoral MNE, and a slightly lower frequency (30.4%) show nutritive phase breakage (Figure S23). Non-nutritive phase breakage, either post-depositional or recent, is very common among the Haile 21A femora. As measured by MNE, proximal epiphyses (n = 37) are rarer than distal epiphyses (n = 59), and most of the complete specimens show no traces of carnivoran damage. Isolated tooth marks are most common, and they occur both with and without associated furrowing (Figure S24)—a pattern also seen among modern large felids [3,6].


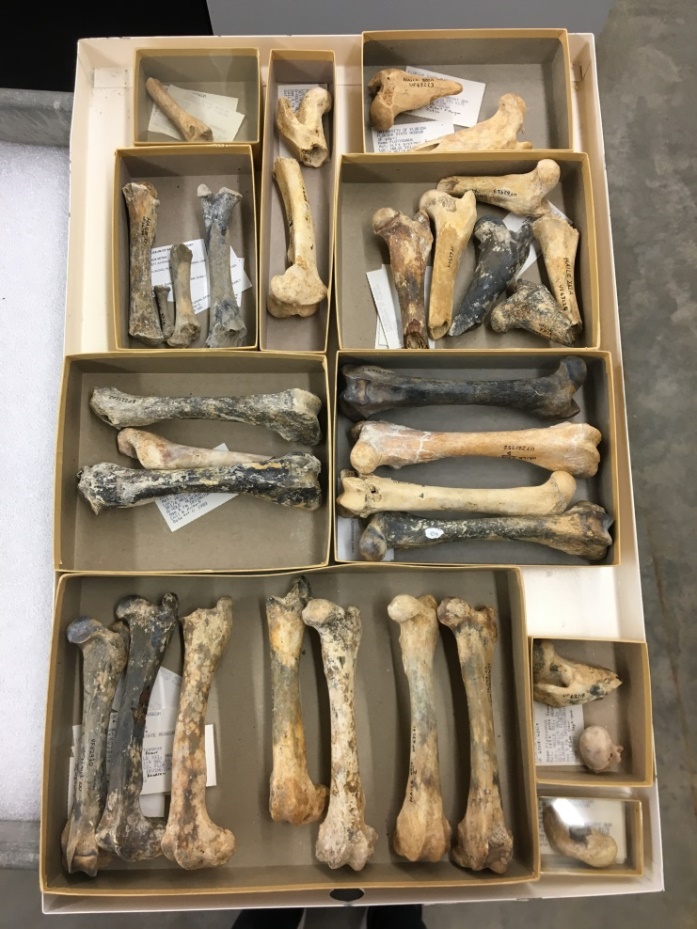


Fig S23. A sample of femur specimens showing typical patterns of preservation.


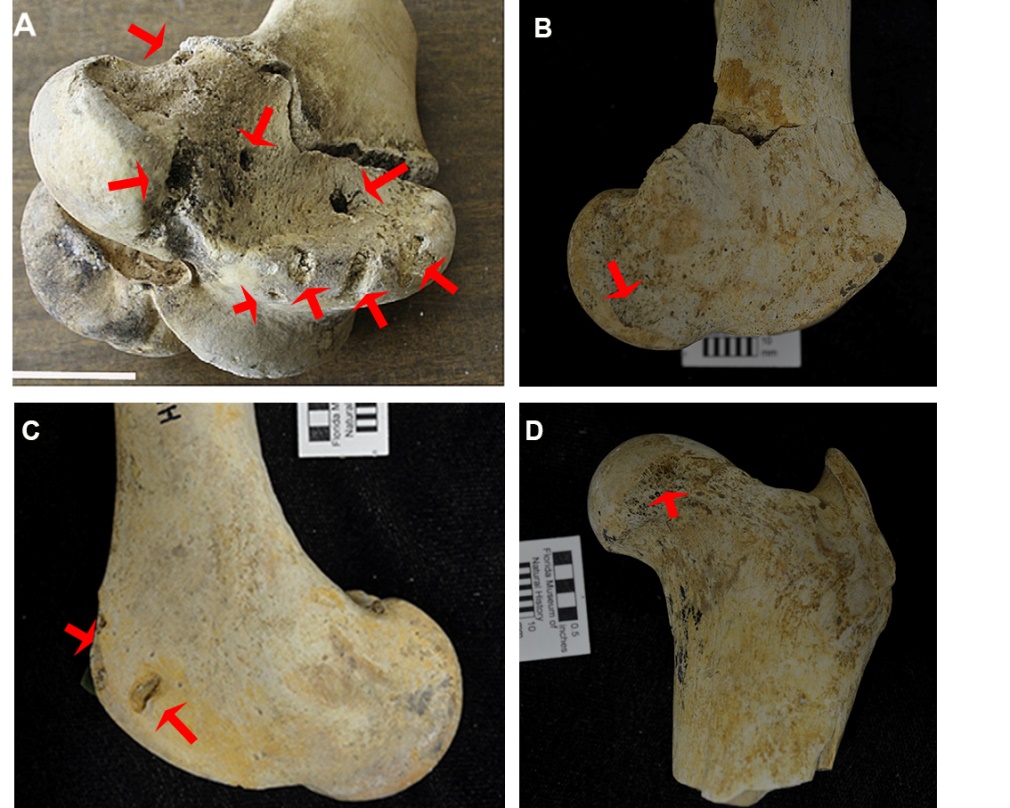


*Fig S24. A, distal femur from an equid consumed by captive lions in Cabárceno (Spain) (Gidna et al. 2013). Notice the presence of tooth pits and scores without associated furrowing. B & C, distal femora from Haile 21A showing tooth marking (red arrows) and a virtual lack of furrowing on the condyles. D, Proximal femur from Haile 21A with a tooth mark near the articular head (red arrow).*

Tibiae

Just over one third (38.5% of tibia MNE) of the Haile 21A tibiae are complete with intact proximal epiphyses and tibial crests. Overall, though, distal epiphyses (n = 82 as measured by MNE) are more common than proximal epiphyses (n = 52), which hints at some density-mediated biasing (Figure S25). Several specimens exhibit only light or moderate furrowing even when the shaft was broken during the nutritive phase (Figure S26). A significant number of tibiae show deletion of the proximal epiphysis in conjunction with various levels of modification to the tibial crest. Nearly all tooth marks appear on proximal portions. One specimen in particular preserves an extremely large, deep puncture on the medial aspect of the proximal end just under the articular surface (Figure S27). A large carnivoran must be responsible and, of the teeth from the four species represented in the Haile 21A assemblage, only the *Xenosmilus* lower canine matches closely the mark’s size and conical shape (Figure S28).


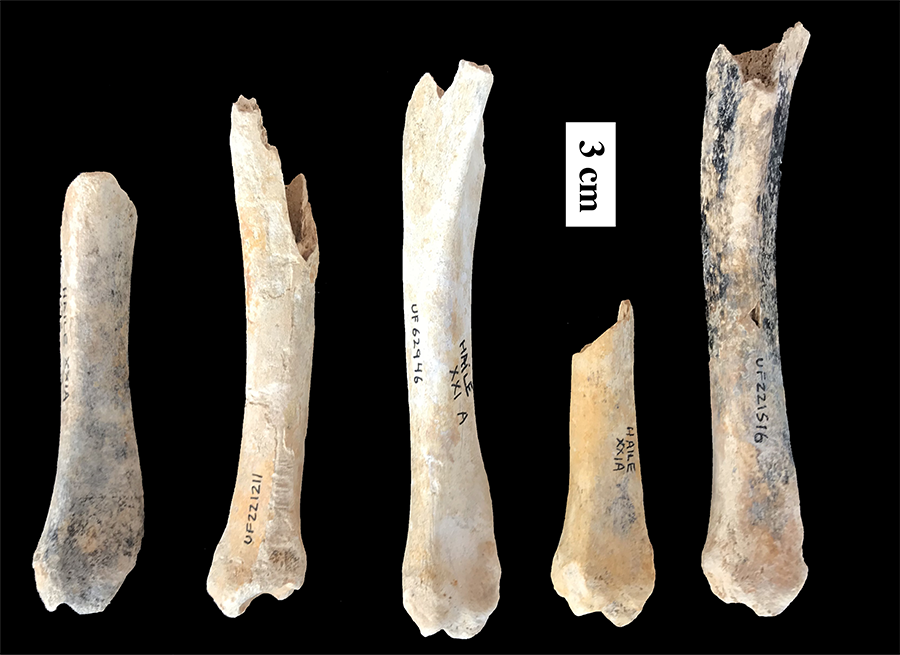


Fig S25. Cranial views of tibiae showing different degrees of destruction of the proximal epiphysis and proximal shaft.


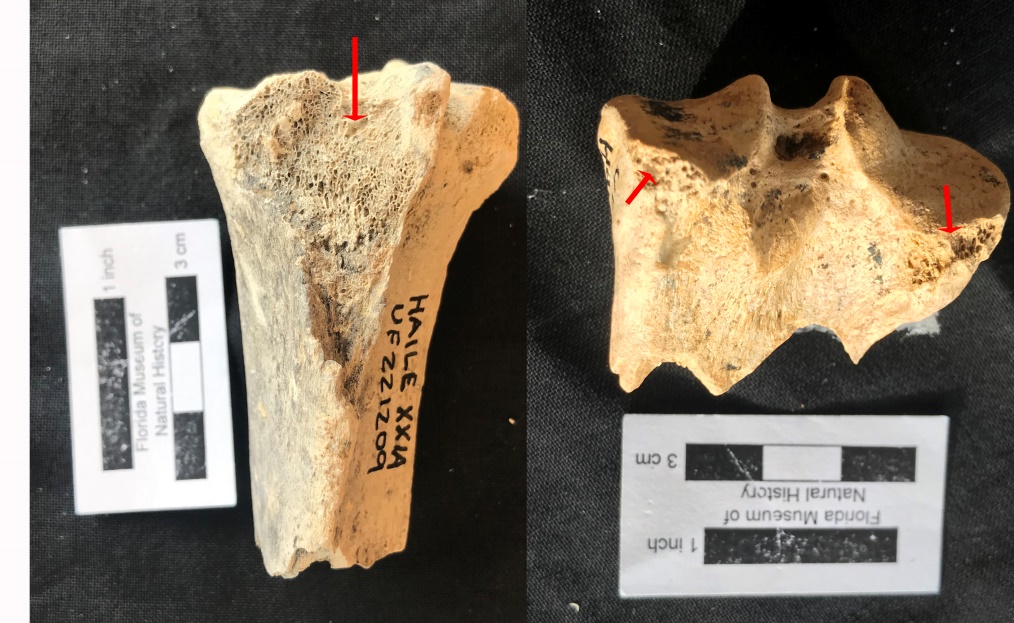


*Fig S26. Examples proximal tibiae with nutritive phase breakage and moderate furrowing of the crest (red arrow, left) or minimal furrowing of the articular rim (red arrows, right).*


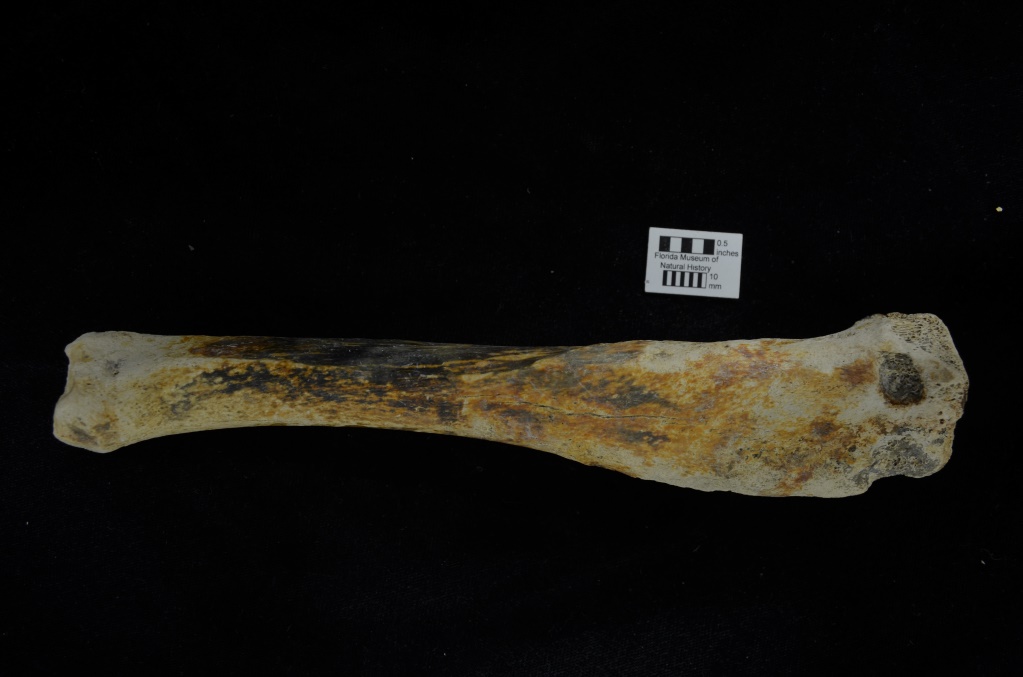


*Fig S27. Medial view of a complete tibia showing two localized modifications on the proximal end: furrowing of the crest and a large puncture on the medial aspect of the epiphysis.*


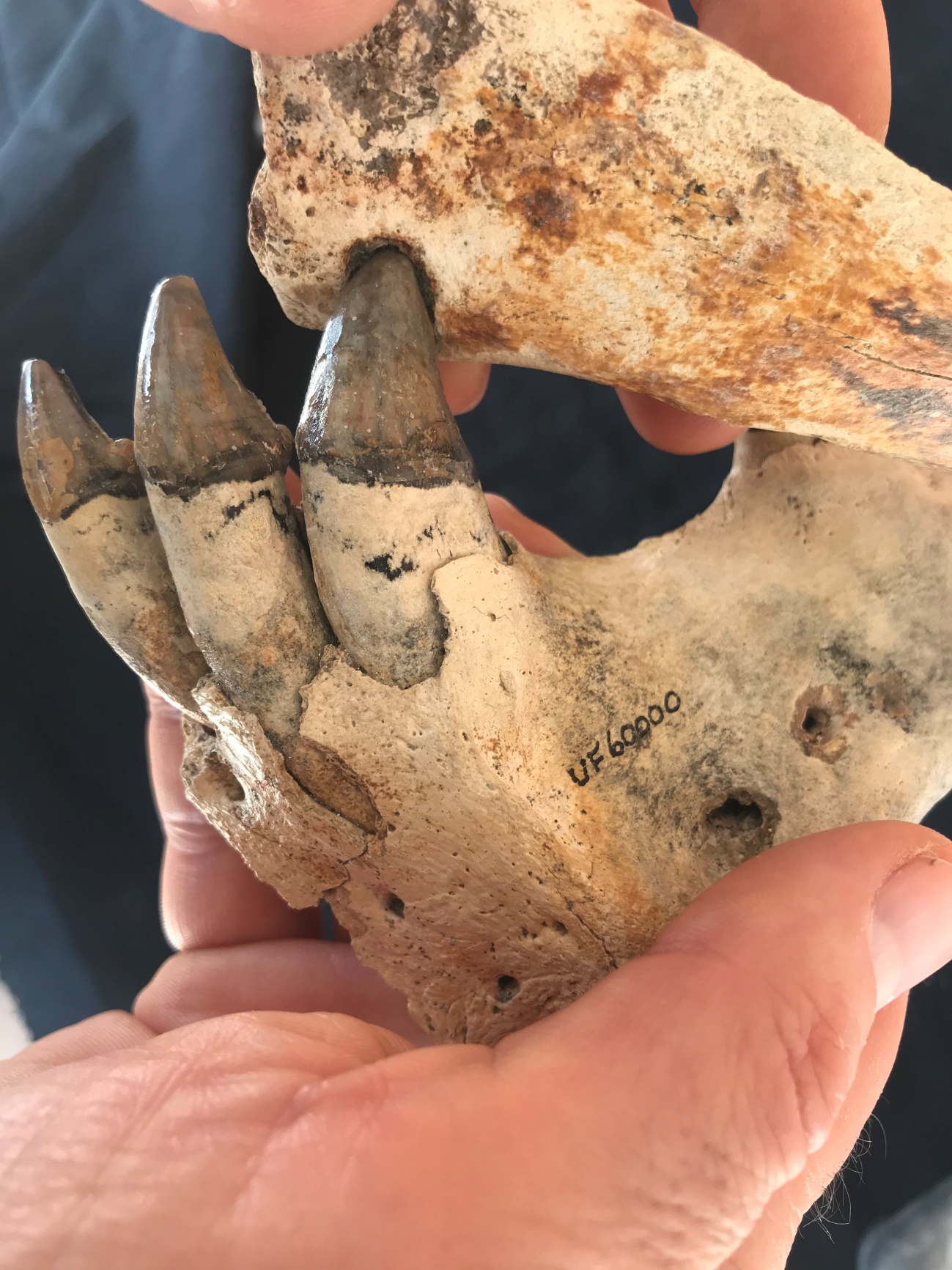


*Fig S28. Fitting of the* Xenosmilus *lower canine into a tooth pit on the proximal tibia pictured in Figure S27.*

Compact bones

With some notable exceptions, nearly all the Haile 21A compact bones are complete, and none, including very small elements like sesamoids, patellae, and phalanges, show the gastric etching that accompanies passage through the digestive tract. A handful of calcanea show tooth pits with associated furrowing of the calcaneal tuber (Figure S29 and S30). Some of these tooth pits exhibit distinctive triangular shapes. A single astragalus preserves a large tooth pit that matches the size and morphology of the *Xenosmilus* lower incisors (Figure S31).


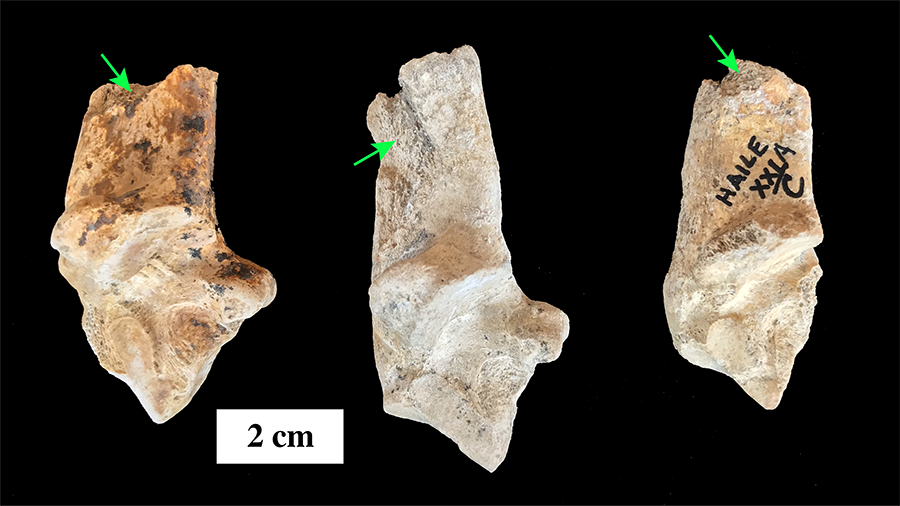


Fig S29. Medial view of calcanea showing moderate furrowing (green arrows) of the proximal end.


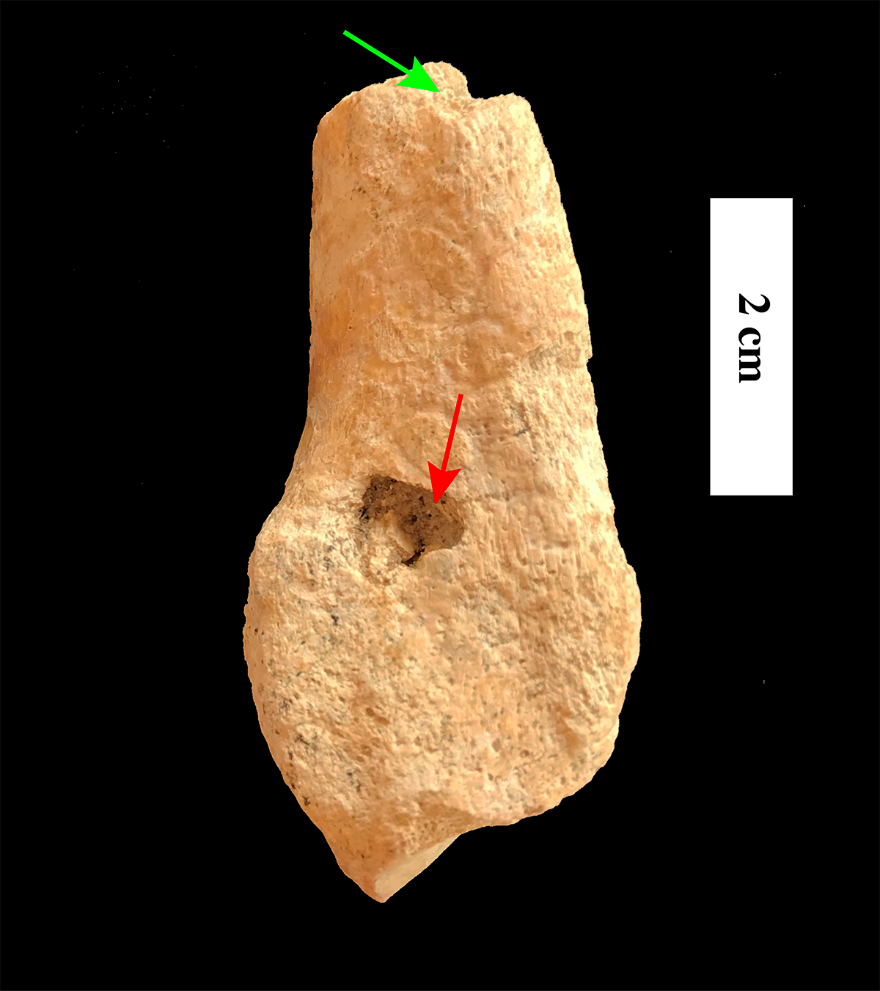


Fig S30. Lateral view of a calcaneus showing a tooth puncture (red arrow) and furrowing of the proximal end (green arrow).


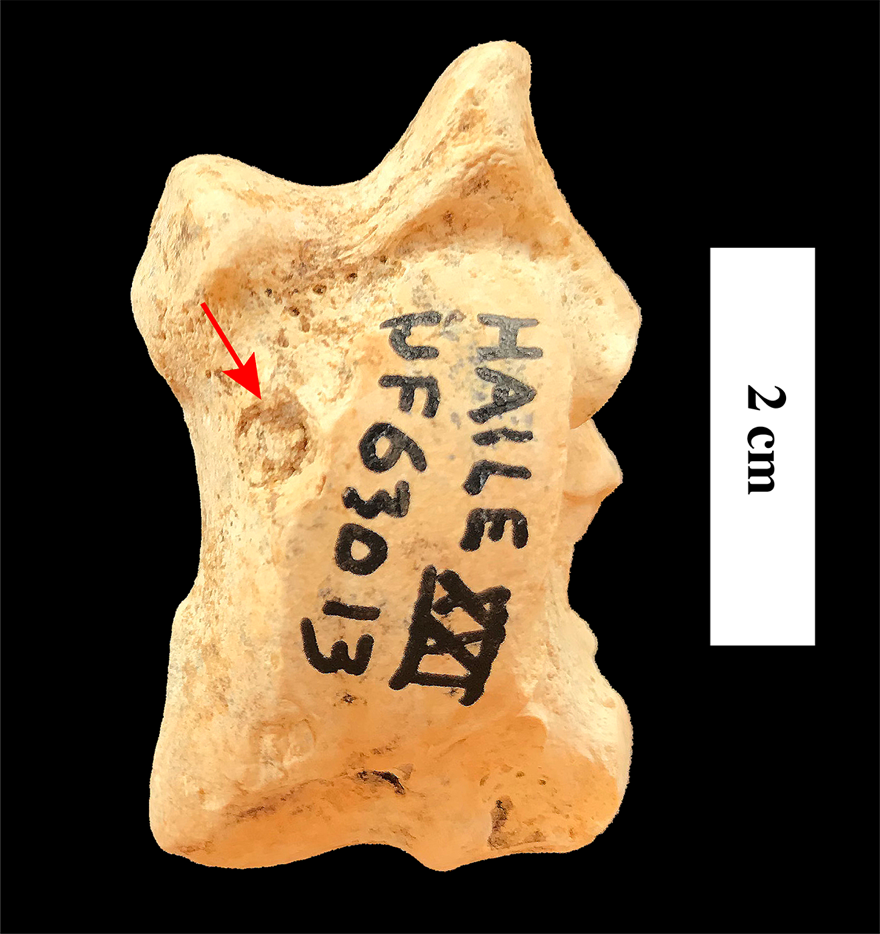


Fig S31. Cranial view of an astragalus showing a tooth pit (red arrow).

Vertebrae

The neural arches and centra of most of the vertebrae in the Haile 21A collection are intact, while the transverse and dorsal processes show frequent breakage (Figure S32 and S33). Tooth marks are rare on these elements, but they do occur on the centra (mainly the ventral aspect) and articular processes (Figure S34). One mark on the ventral aspect of the centrum of a lumbar vertebra matches very closely the size and shape of the mesial cusp of the *Xenosmilus* carnassial (Figure S35). Another mark, which appears on the caudal surface of the centrum of a cervical vertebra, reveals at least one instance of vertebral column disarticulation. A large tooth pit on the dorsal aspect of the wing of an atlas vertebra (Figure S36) is particularly interesting as it signals tooth-on-bone contact to the nape. Nutritive phase breakage, where it could be reliably identified, clusters on the neural arches, the articular processes and, less frequently, the centra. The wings of sacra are also furrowed and tooth-marked. An elongated tooth mark documented on one sacral specimen may correspond to the *Xenosmilus* carnassial (Figure S37).


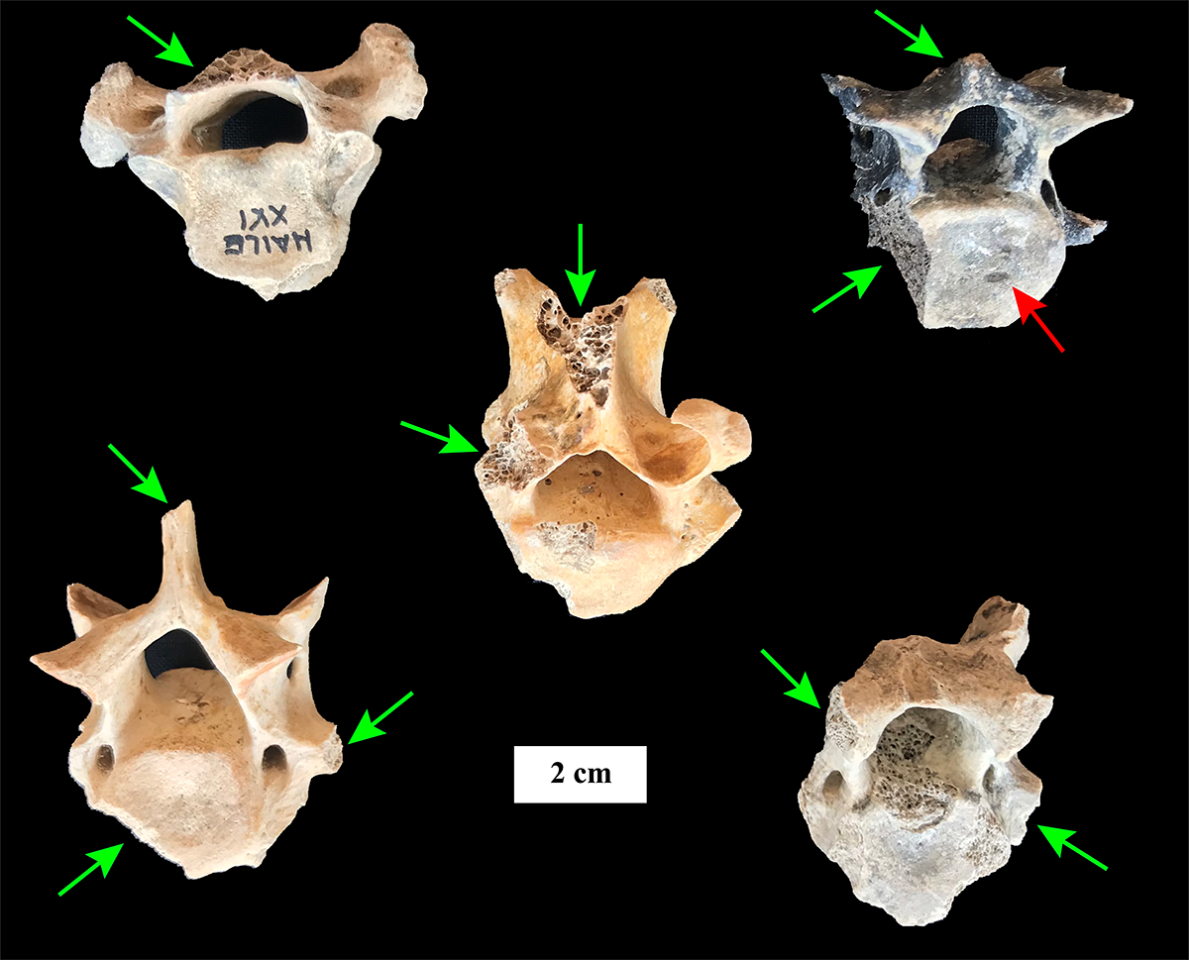


Fig S32. Examples of cervical vertebrae showing furrowing (green arrows) and a tooth mark (red arrow).


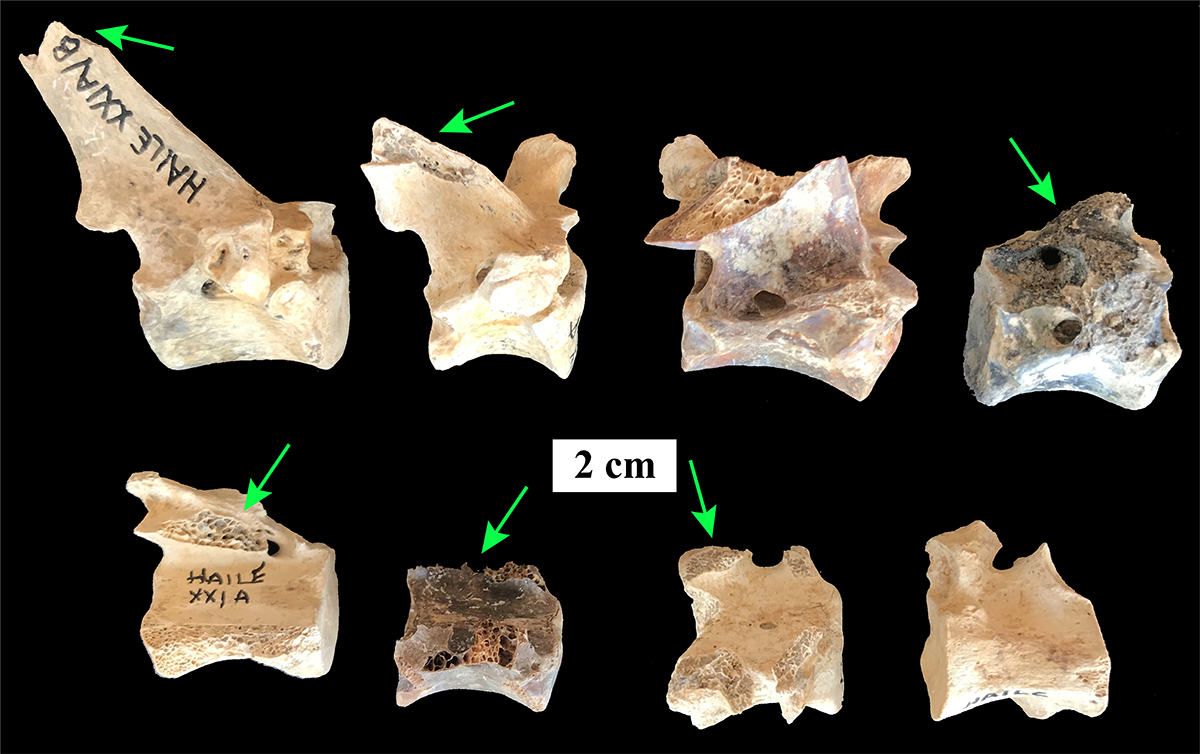


Fig S33. Examples of thoracic vertebrae showing furrowing (green arrows).


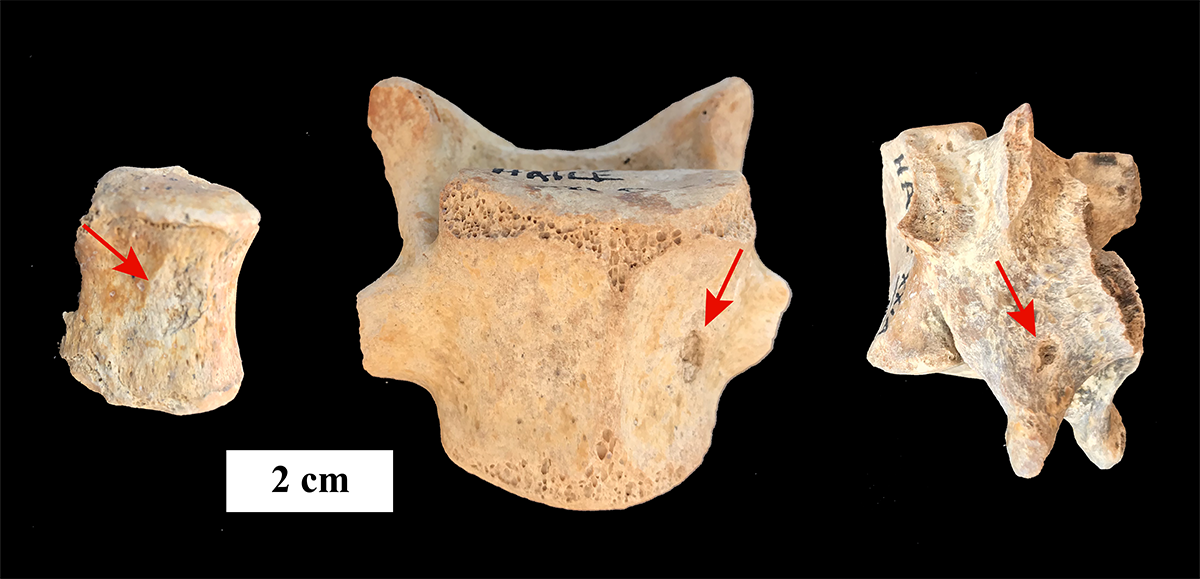


Fig S34. Vertebrae with tooth marks (red arrows) on the centra (left, center) and neural arch (right).


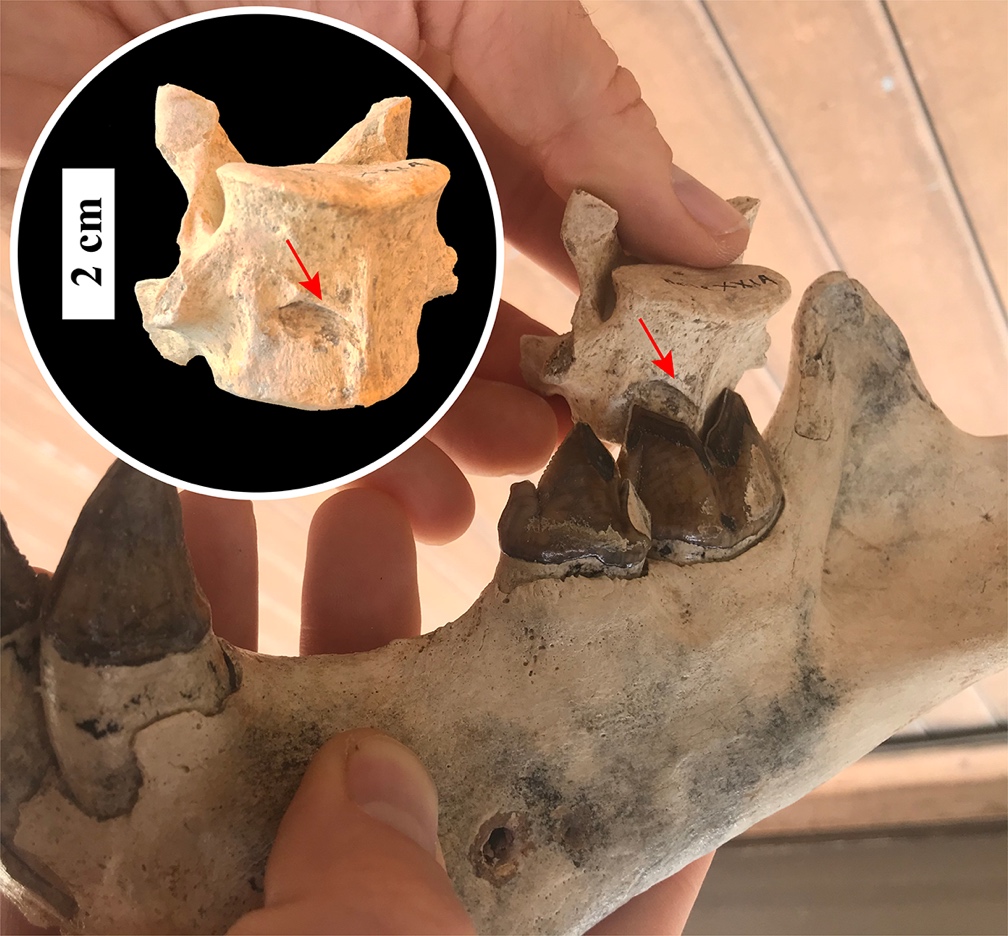


Fig S35. Lumbar vertebra with tooth pit on the ventral surface (red arrows) that matches the mesial ridge of the Xenosmilus carnassial.


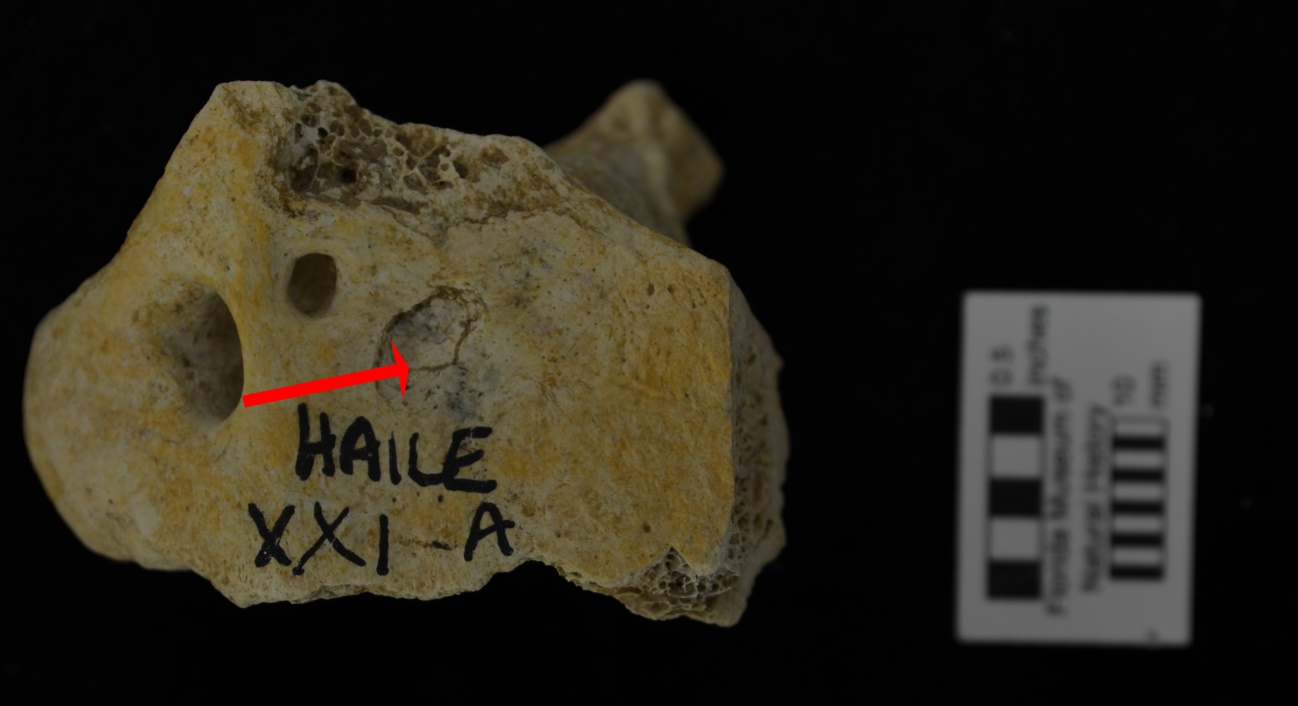


*Fig S36. Atlas showing a large tooth pit (red arrow).*


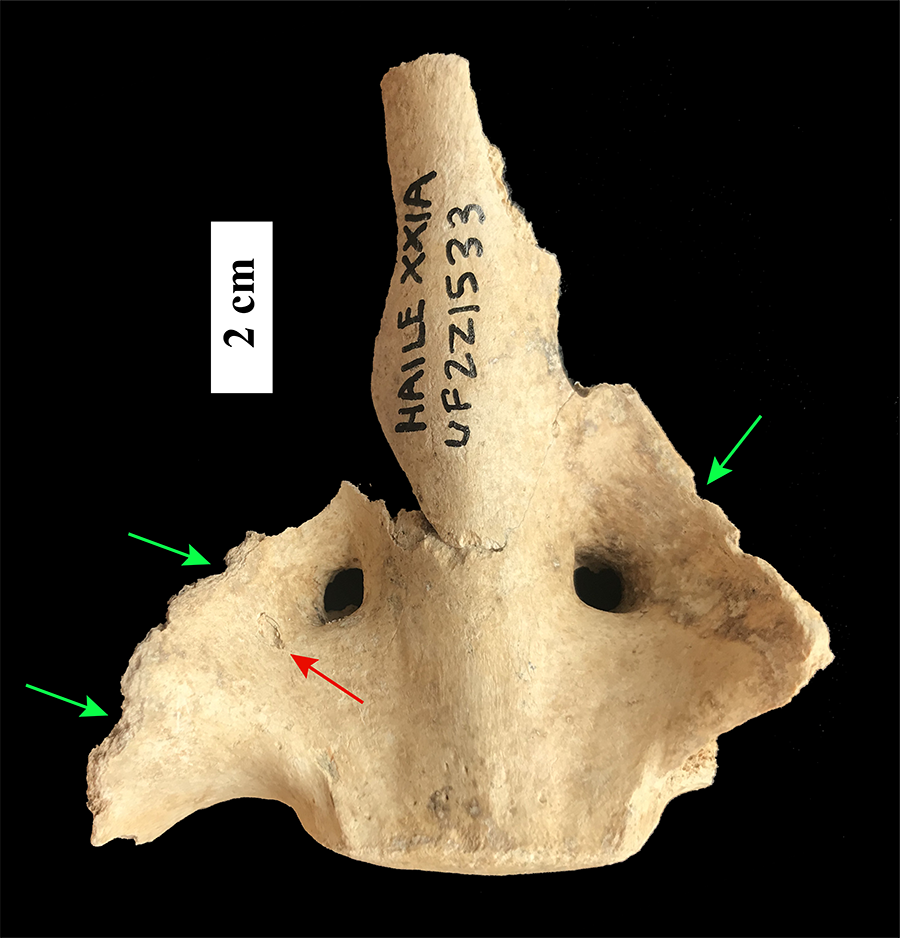


Fig S37. Ventral view of a sacrum showing tooth mark (red arrow) and furrowing (green arrows).

Ribs

Ribs in the Haile 21A faunal assemblage are abundant but typically fragmented to various degrees (Figure S38). The head and tubercle are by far the most commonly preserved features, probably due to selective collection during excavation. Tooth marks are rare on *Platygonus* ribs, although a series of marks does appear on the proximal blade of a Size Class 3 mammal (Figure S39). Very little damage occurs on the heads and tubercles despite the presence of nutritive phase breakage to many of the blades. This is unexpected if the main taphonomic actor was a durophagous carnivoran.


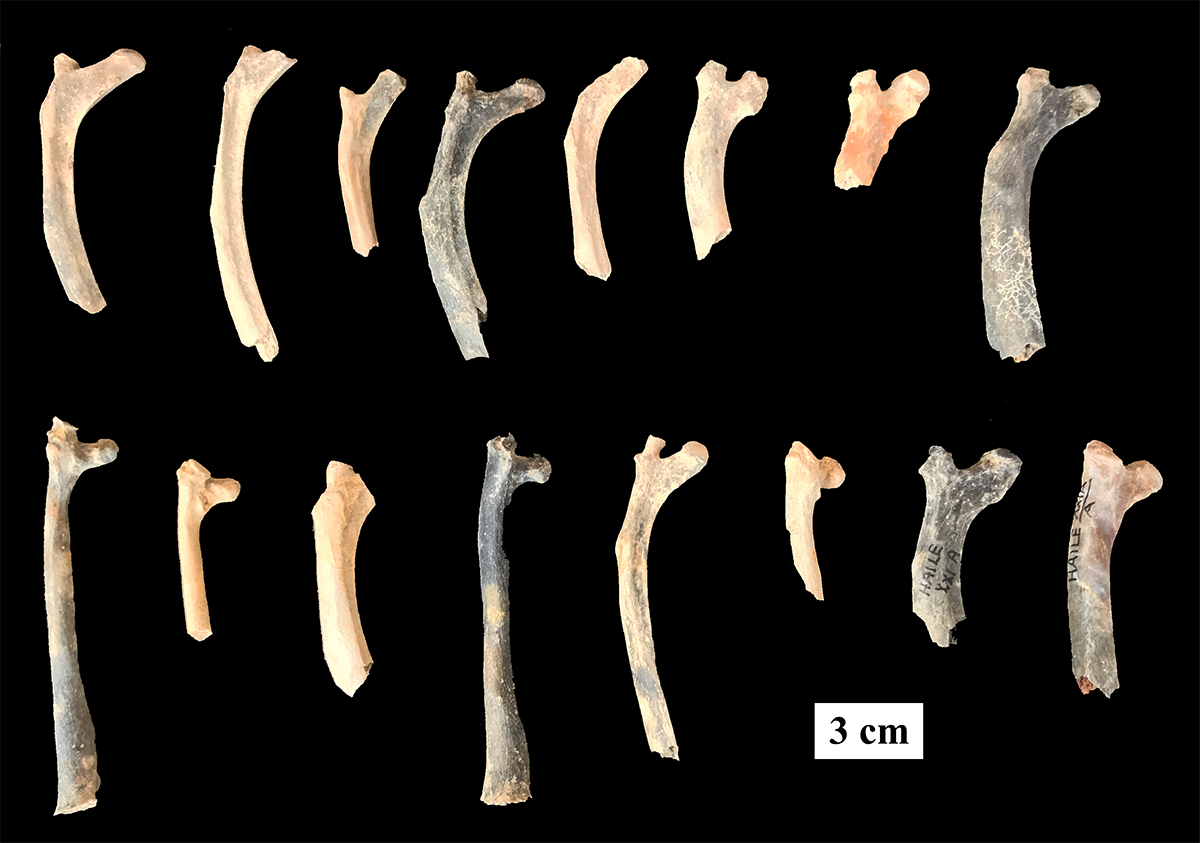


Fig S38. A sample of rib specimens showing typical patterns of preservation.


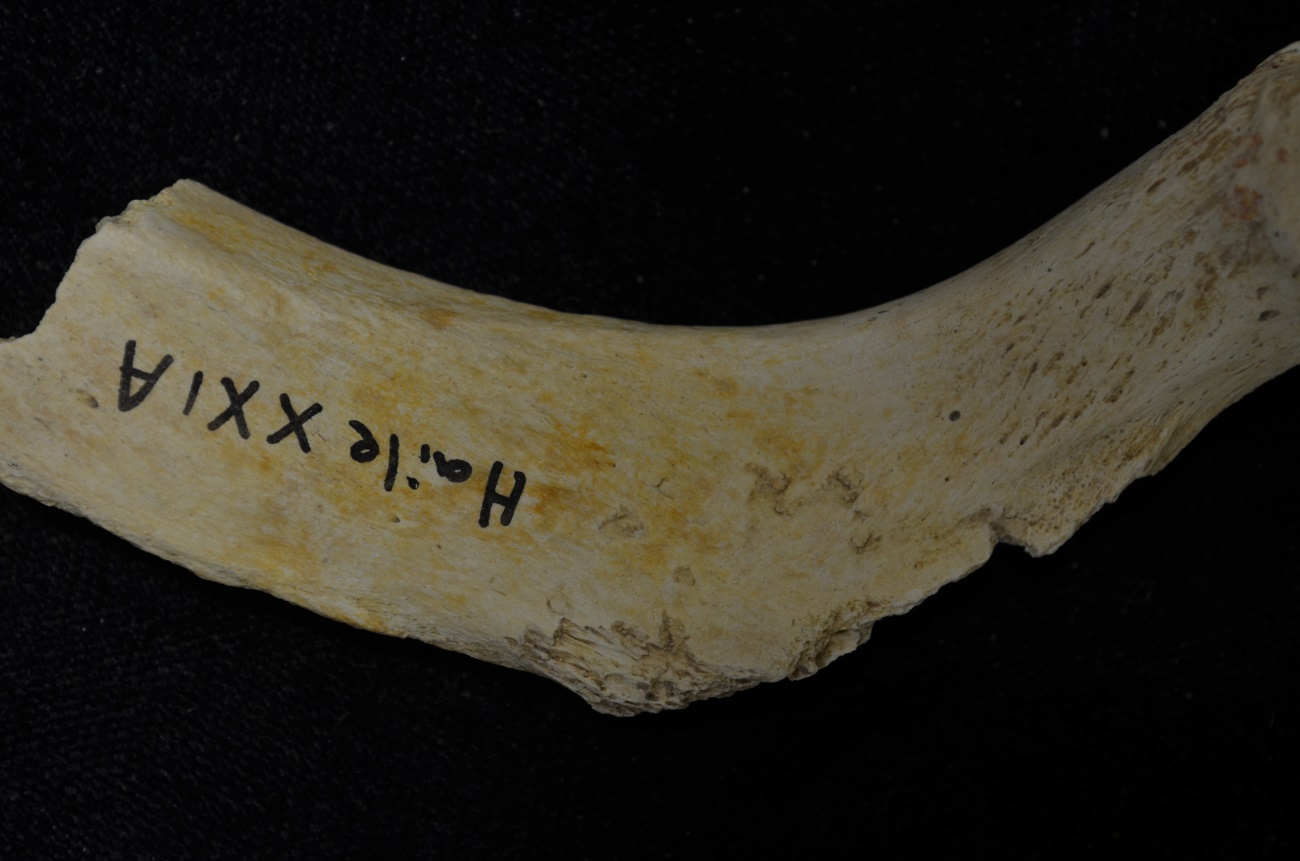


Fig S39. *Proximal rib from a medium-sized mammal with tooth marks (blue arrows) and some marginal furrowing (red arrow).*

Metapodials

Many of the Haile 21A metapodials are complete and free of tooth marks (Figure S40). Nutritive phase breakage, when it does occur, is present exclusively on the distal epiphyses (Figures S41 and Figure S42). In some cases, this breakage is associated with a few tooth marks, which indicates some consumption of the feet.


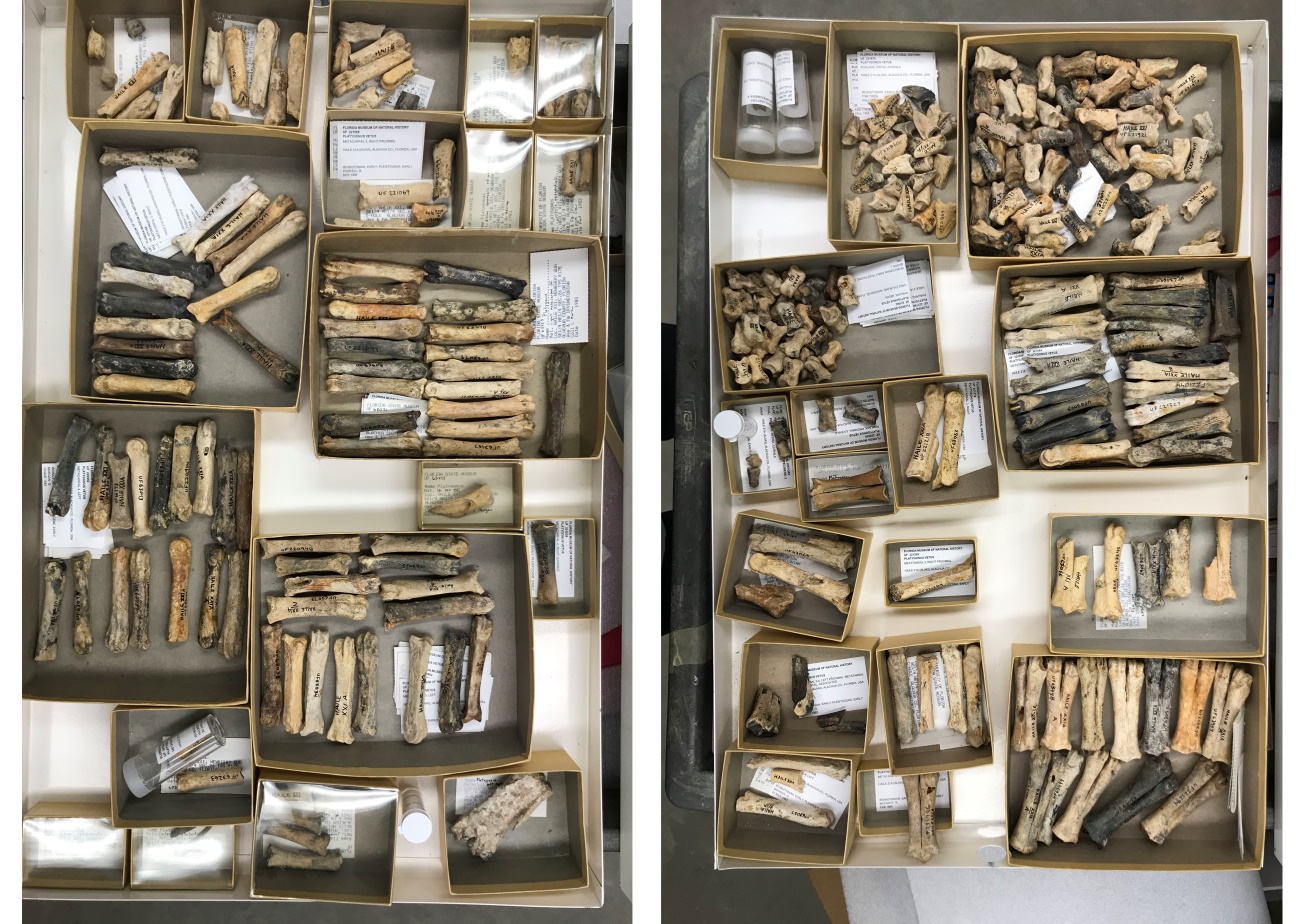


Fig S40. A sample of metacarpal (left) and metatarsal and phalanx (right) specimens showing typical patterns of preservation.


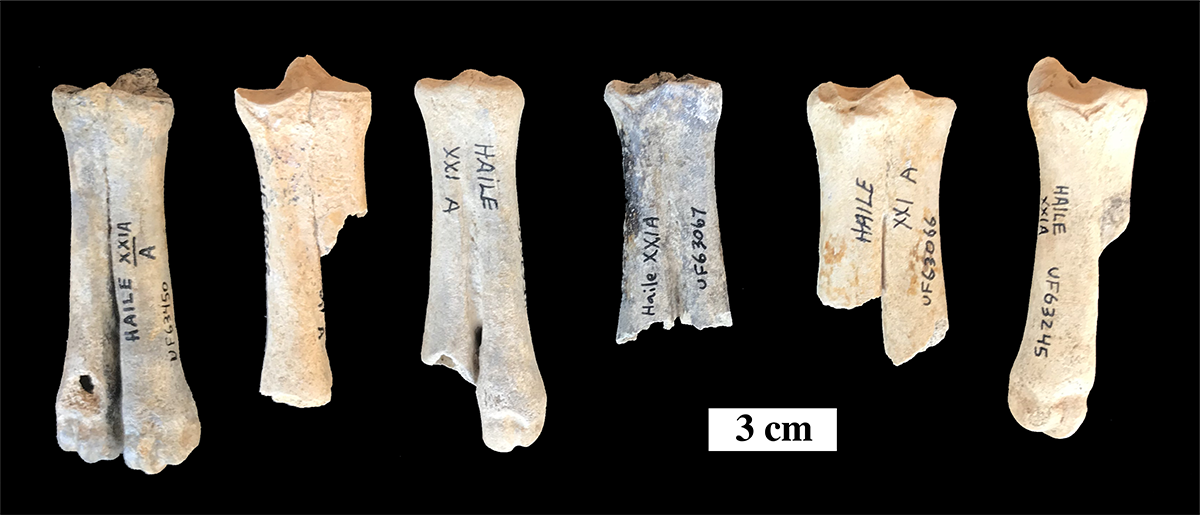


Fig S41. Sample of metatarsal specimens showing nutritive phase breakage on the distal ends.


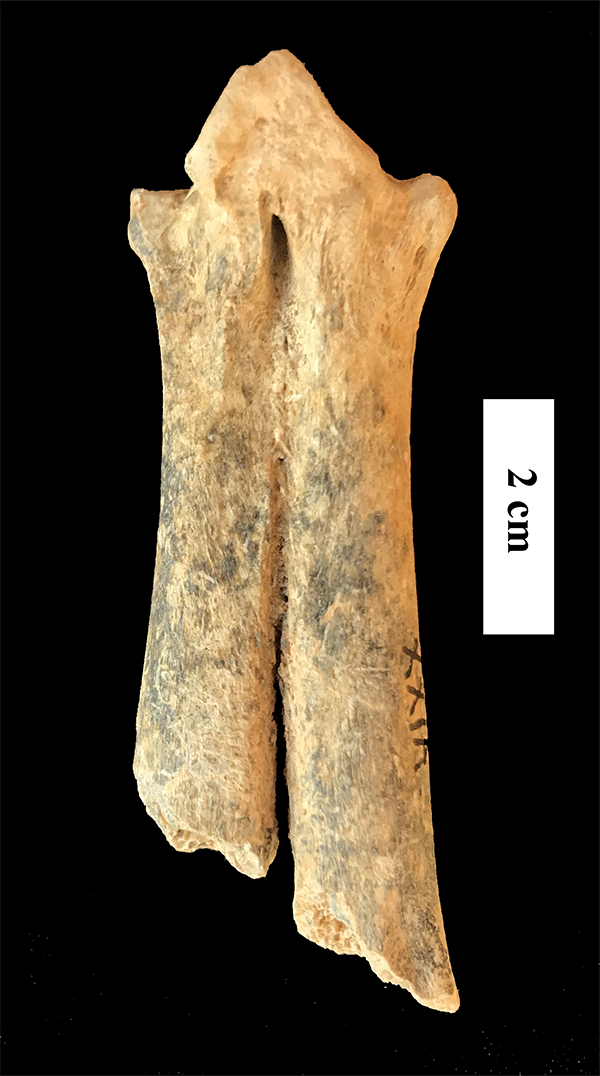


Fig S42. Caudal view of a metatarsal showing an oblique nutritive phase break that spans the distal end of both metatarsal three and metatarsal four.

*Tooth mark dimensions*

Haile 21A tooth mark sizes compared to modern tooth mark sizes

The Haile 21A assemblage preserves a total of 109 measurable tooth pits and 31 measurable tooth scores, and we divide this into three subsamples: those occurring on the diaphysis, metaphysis, and/or dense epiphyseal portions (e.g., distal humerus) of long bones, those on thin-walled portions of long bones with extensive trabeculae (e.g., the femoral trochanters, the humeral tuberosities), and those on axial elements. Most of the tooth pit distributions are positively skewed (Figure S43), so we utilize both the mean and median as a measure of central tendency. For tooth score breadth, we use the mean only. We report 95% confidence intervals for the modern comparative samples. The length and breadth of the Haile 21A tooth pits on less dense long bone epiphyses fall above the boundary identified by Andrés et al. [8] that differentiates carnivorans with body weights <40kg from those with body weights >40kg (Figure S44 and Table S9). More specifically, the Haile 21A tooth pits compare most favorably with those produced by spotted hyenas and lions and outside the range of those produced by gray wolves.


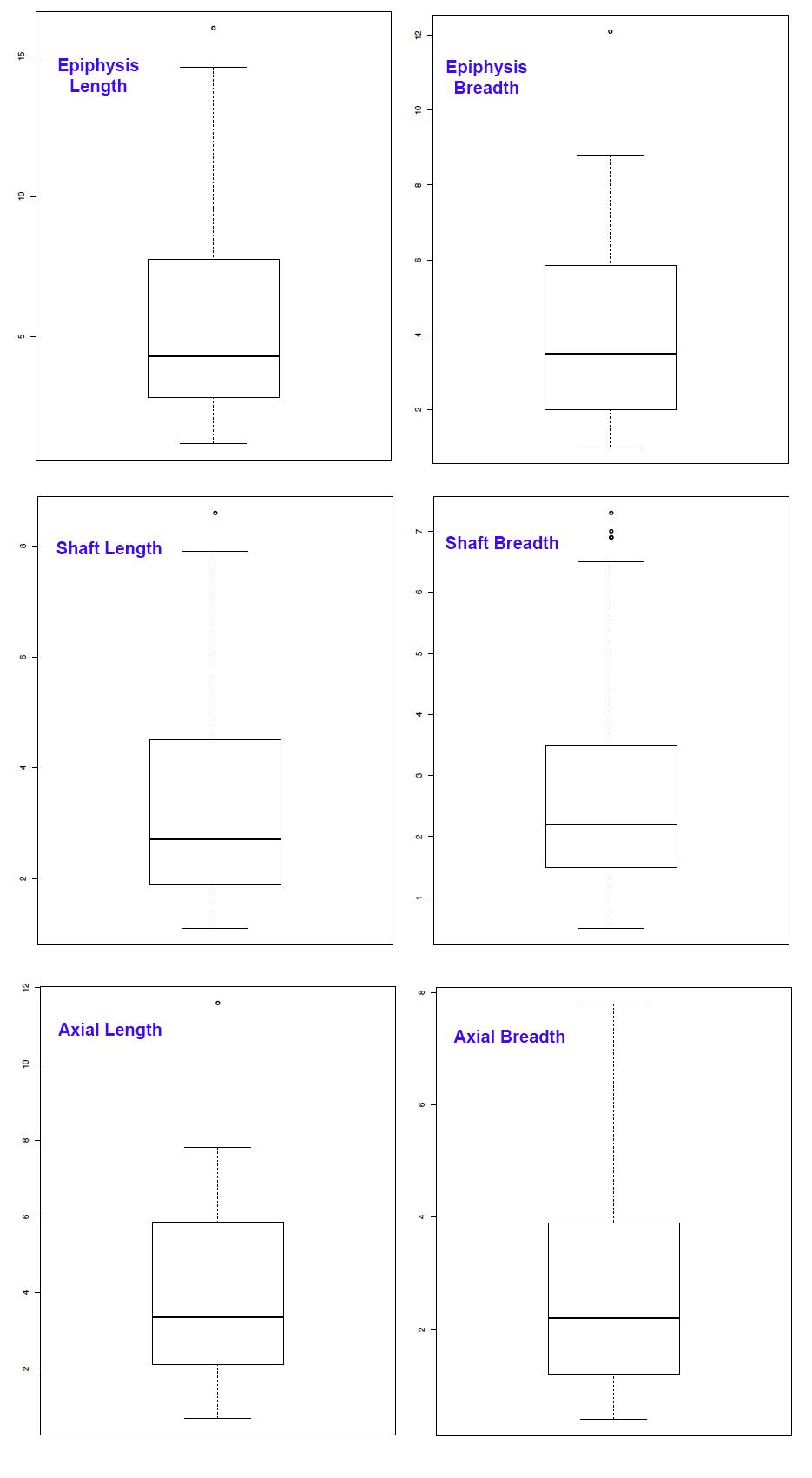


*Fig S43. Boxplots showing the sample distribution for length and breadth of tooth pits on epiphyseal, shaft, and axial portions and elements of the Haile 21A* Platygonus *collection.*


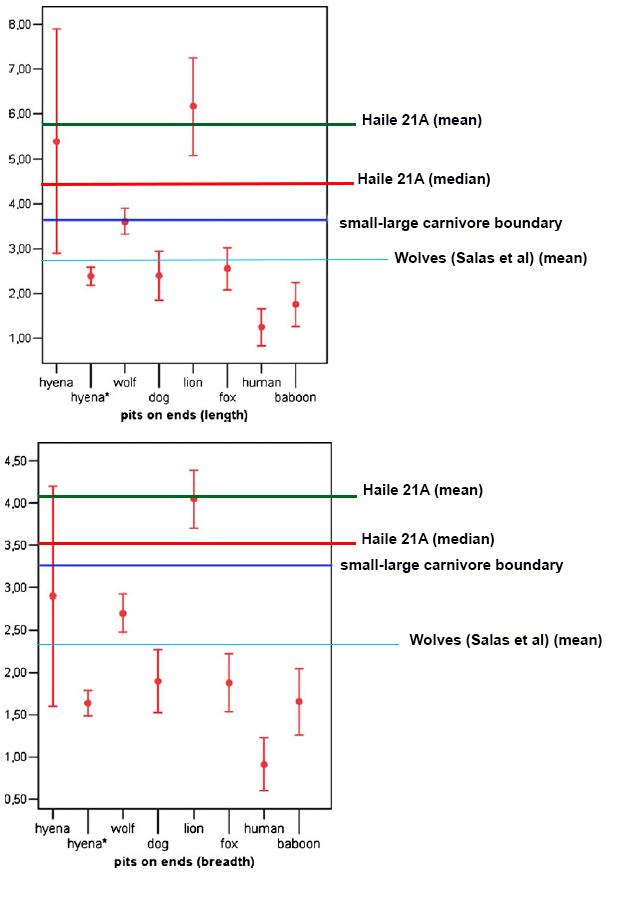


*Fig S44. Tooth pit size for length (upper) and breadth (lower) on epiphyseal portions for modern carnivorans (Andres et al. 2021) and the Haile 21A* Platygonus *collection. Mean value for wolves reported by Sala et al.* (2014) *is also included. *Bones modified by spotted hyena cubs.*

The length and breadth of the Haile 21A tooth pits on denser long bone portions again fall above the small/large carnivoran boundary of Andrés et al. [8]. For length, the Haile 21A tooth pits fall within with the 95% confidence intervals of lions and spotted hyenas, while for breadth they fall above the values reported for all the modern carnivorans (Figure S45). Tooth pits on axial remains (scapulae, innominates, vertebrae, and ribs) are only slightly larger than those on denser long bone portions and substantially smaller than those on less dense long bone epiphyses. The latter finding is unexpected given the similarity in trabecular/cancellous structure of axial elements and long bone epiphyses.


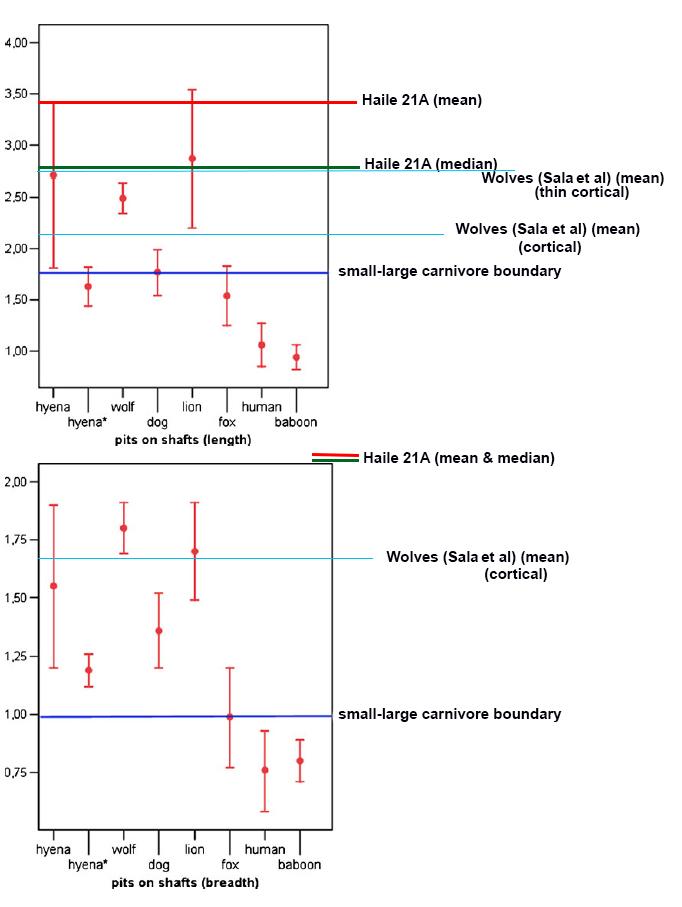


*Fig S45. Tooth pit size for length (upper) and breadth (lower) on shaft portions for modern carnivorans (Andres et al. 2012) and the Haile 21A* Platygonus *collection. Mean value for wolves reported by Sala et al.* (2014) *is also included. *Bones modified by spotted hyena cubs.*

*Table S9. Mean, median and 95% confidence intervals for length and breadth of tooth pits on epiphyseal and diaphyseal portions of long bones and axial elements.*

|  | Mean | Median | Lower 95% CI | Upper 95% CI |
| --- | --- | --- | --- | --- |
| Epi Length | 5.78 | 4.3 | 4.36 | 7.19 |
| Epi Breadth | 4.14 | 3.5 | 3.16 | 5.12 |
| Shaft Length | 3.46 | 2.72 | 2.82 | 4.09 |
| Shaft Breadth | 2.49 | 2.2 | 2.34 | 3.55 |
| Axial Length | 3.98 | 3.35 | 3.14 | 4.83 |
| Axial Breadth | 2.66 | 2.2 | 2.06 | 3.25 |

| All portions length | 4.31 | 3.3 | 3.73 | 4.86 |
| --- | --- | --- | --- | --- |
| All portions breadth | 3.20 | 2.8 | 2.78 | 3.62 |

Given the small sample of tooth scores, we combine those on the axial remains and the less dense long bone epiphyses into a single analytical unit. The mean breadths of tooth scores on denser long bone portions and cancellous portions are 1.1 mm and 2.0 mm, respectively. The Haile 21A tooth scores on cancellous bone portions are narrower than those documented for gray wolves and lions (Figure S46). Tooth score breadth on denser long bone portions is narrower than that of gray wolves and slightly broader than that of lions. The dimensions of tooth pits and tooth scores together indicate that a large carnivoran—more similar in size to a lion than a gray wolf—generated many of the tooth marks on the Haile 21A fauna.


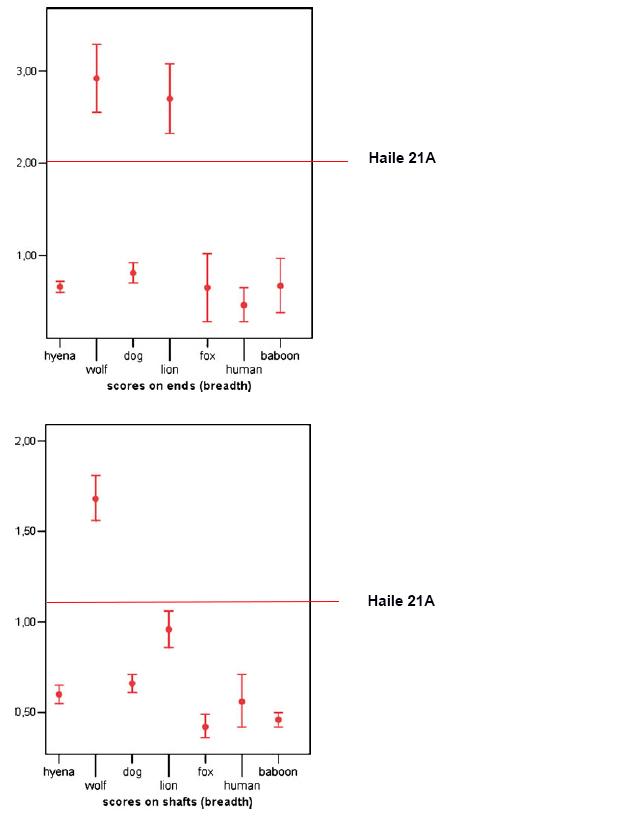


*Fig S46. Tooth score breadth on cancellous (upper) and cortical (lower) shaft portions for modern carnivorans* (Andres et al. 2012) *and the Haile 21A* Platygonus *collection.*

Haile 21A tooth mark sizes compared to fossil carnivoran tooth mark sizes

Figures S47 and S48 display, respectively, mandibular specimens of *Canis edwardii* and *Xenosmilus hodsonae* excavated from Haile 21A. When the marks created by all teeth are combined for each of the fossil carnivorans, the tooth pits of *Xenosmilus* are significantly larger than those of either *Smilodon* or *Canis* (Table S10). The 95% confidence interval for tooth pit length and width overlap for *Smilodon* and *Canis*. The Haile 21A tooth pits (denser long bone portions, less dense long bone portions, and axial elements combined) compare most favorably to those of *Xenosmilus* (Figure S49).


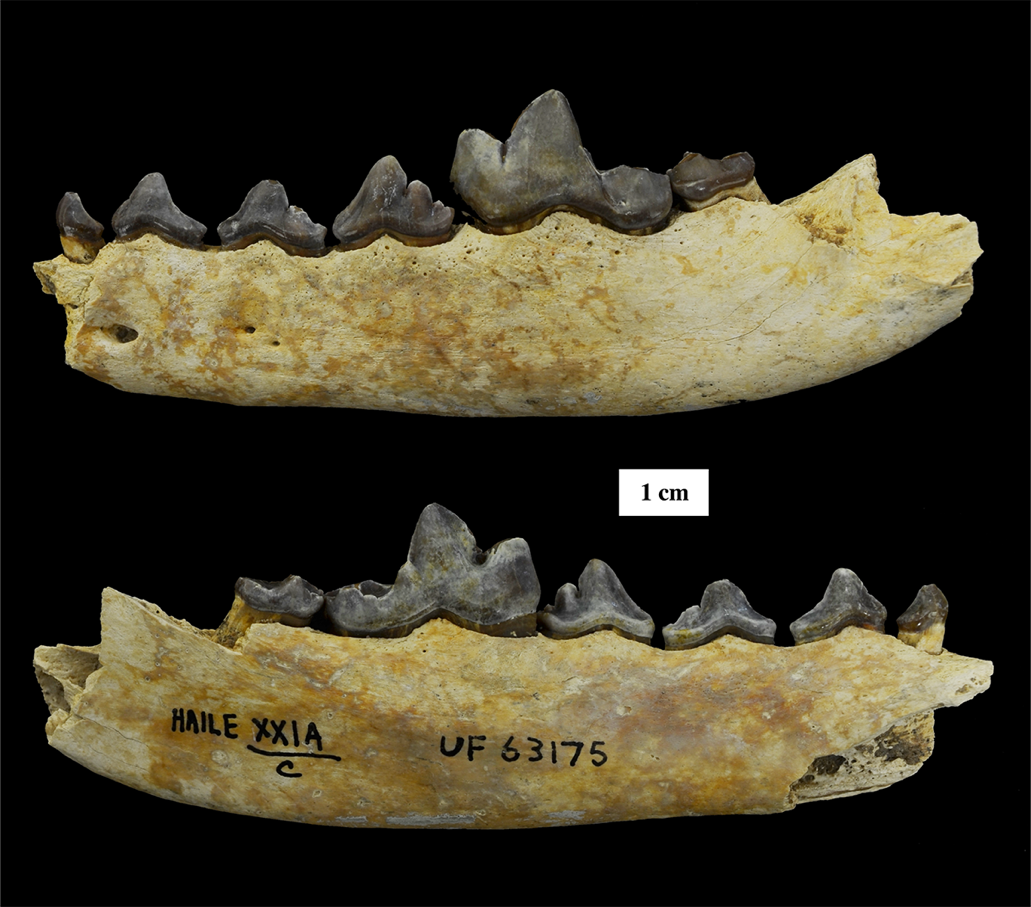


Fig S47. Buccal (top) and lingual (bottom) views of Canis edwardii mandible from Haile 21A.


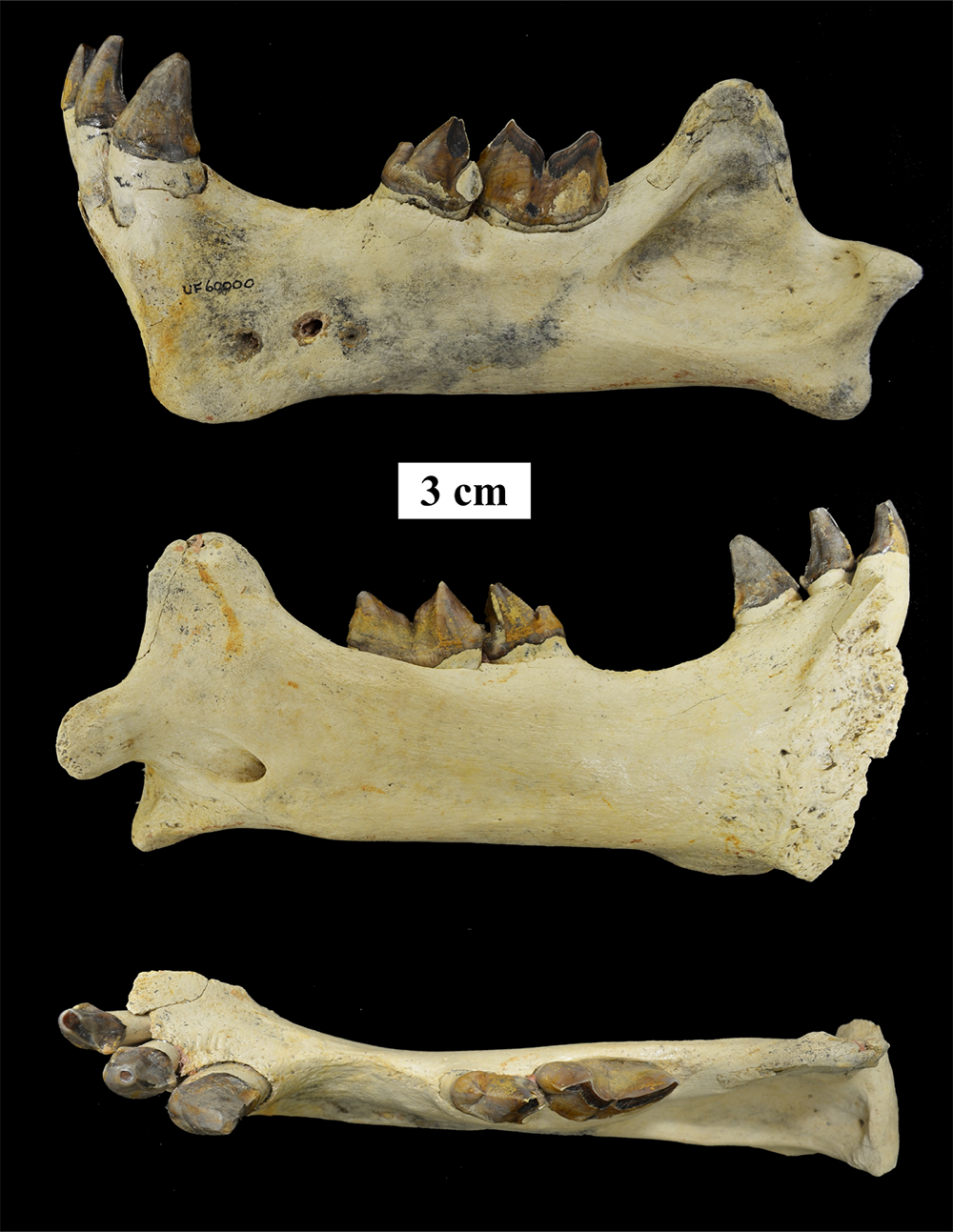


Fig S48. Buccal (top), lingual (middle), and occlusal (bottom) views of Xenosmilus hodsonae mandible from Haile 21A.


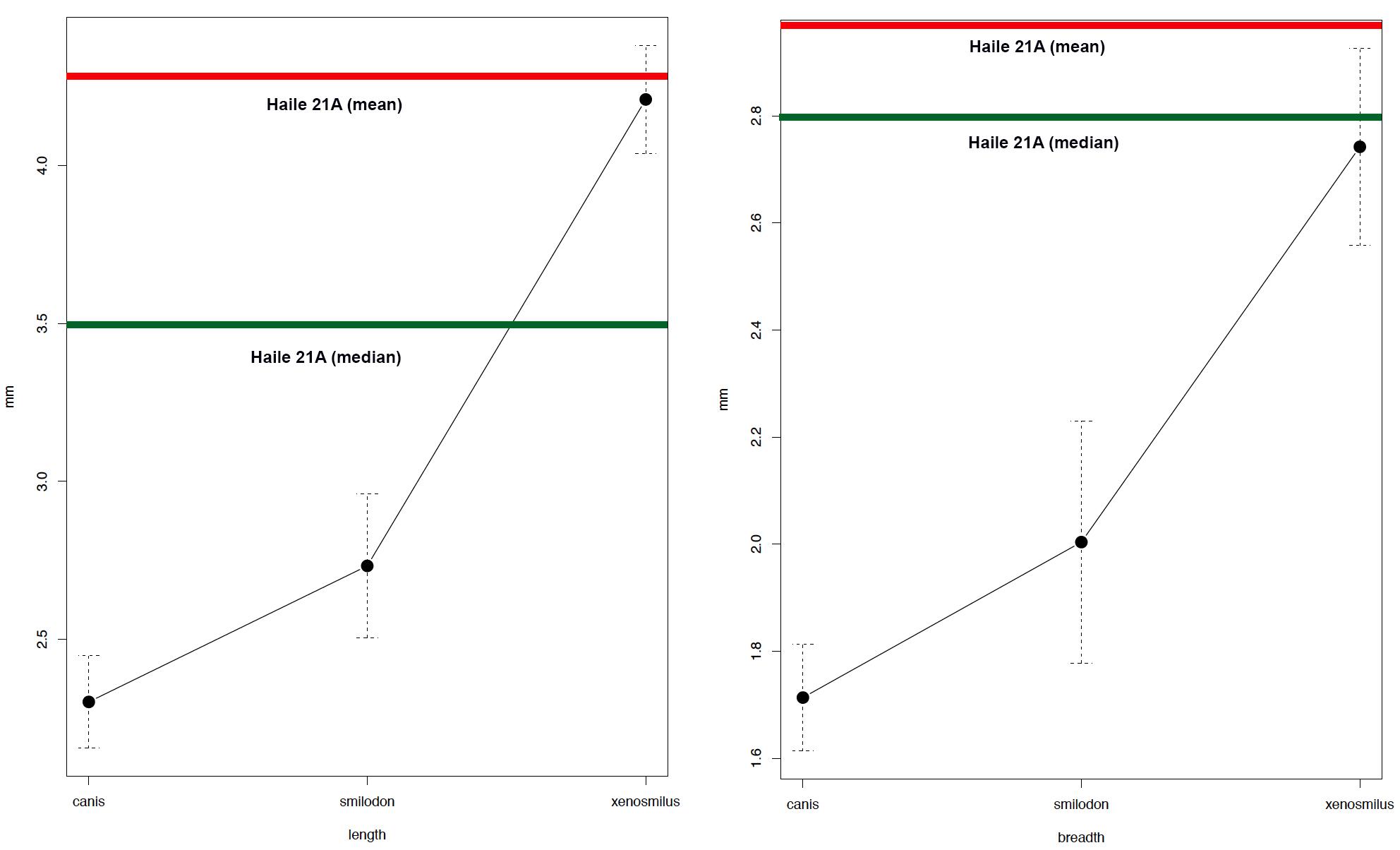


*Fig S49. Length (left) and breadth (right) of the reproduced tooth pits on clay surfaces for all the teeth used for* Canis edwardii*,* Smilodon gracilis, *and Xenosmilus hodsonae. Confidence intervals (95%) are also shown. The mean (red) and median (green) values for the Haile 21A tooth marks are also displayed.*

*Xenosmilus* shows variability in tooth pit size by tooth type, with the P_4_ and M_1_ associated with the smallest marks and the incisors with the largest marks (Figure S50). The Haile 21A tooth pits that appear on less dense long bone portions most commonly fall within the range of those produced by the *Xenosmilus* anterior dentition. The tooth pits of *Canis edwardii* are substantially smaller than those of *Xenosmilus* across the board (Figure S51). The case of *Smilodon* is interesting (Figure S52). The mean length of tooth pits on less dense long bone portions in the Haile 21A assemblage is well above the mean and 95% confidence interval of any of the *Smilodon* teeth we sampled, and the median value of the Haile 21A tooth pits lies at the upper end of the *Smilodon* range. Only the size of tooth pits on denser long bone portions of the Haile 21A sample fall within the *Smilodon* range. Given that the clay plaques are poor proxies for dense cortical bone, we do not put much stock in this similarity.


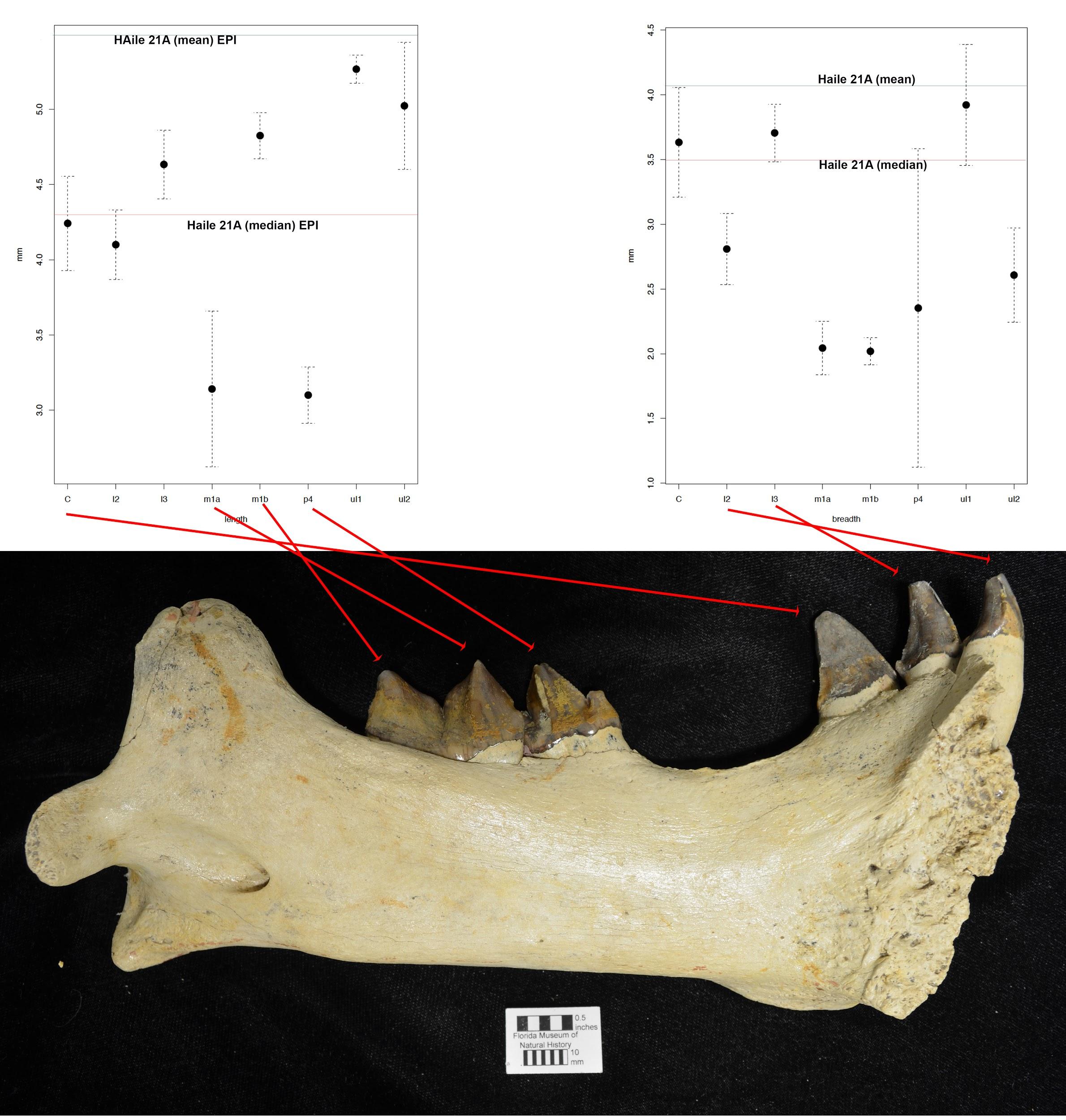


*Fig S50.* Xenosmilus *hemimandible found at Haile 21A. The dimensions (length and breadth) of the tooth marks made on the clay plaque according to each tooth type are shown, together with their confidence intervals. Arrows point to specific teeth and the dimensions of their resulting tooth marks. Only the upper first and second incisors appear in the graphs without reference to any figure.*

*Table S10. Mean and confidence intervals for the reproduced tooth marks made with the dentition of the three Haile 21A macromammal carnivorans.*

|  | Dimension | mean | Lower 95% CI | Upper 95% CI |
| --- | --- | --- | --- | --- |
| *Xenosmilus hodsonae* | length | 4.20 | 4.00 | 4.40 |
| *n=159* | breadth | 2.74 | 2.55 | 2.92 |
| *Canis edwardii* | length | 2.30 | 2.15 | 2.44 |
| *n=89* | breadth | 1.71 | 1.61 | 1.81 |
| *Smilodon gracilis* | length | 2.72 | 2.50 | 2.95 |
| *n=52* | breadth | 2.00 | 1.77 | 2.23 |


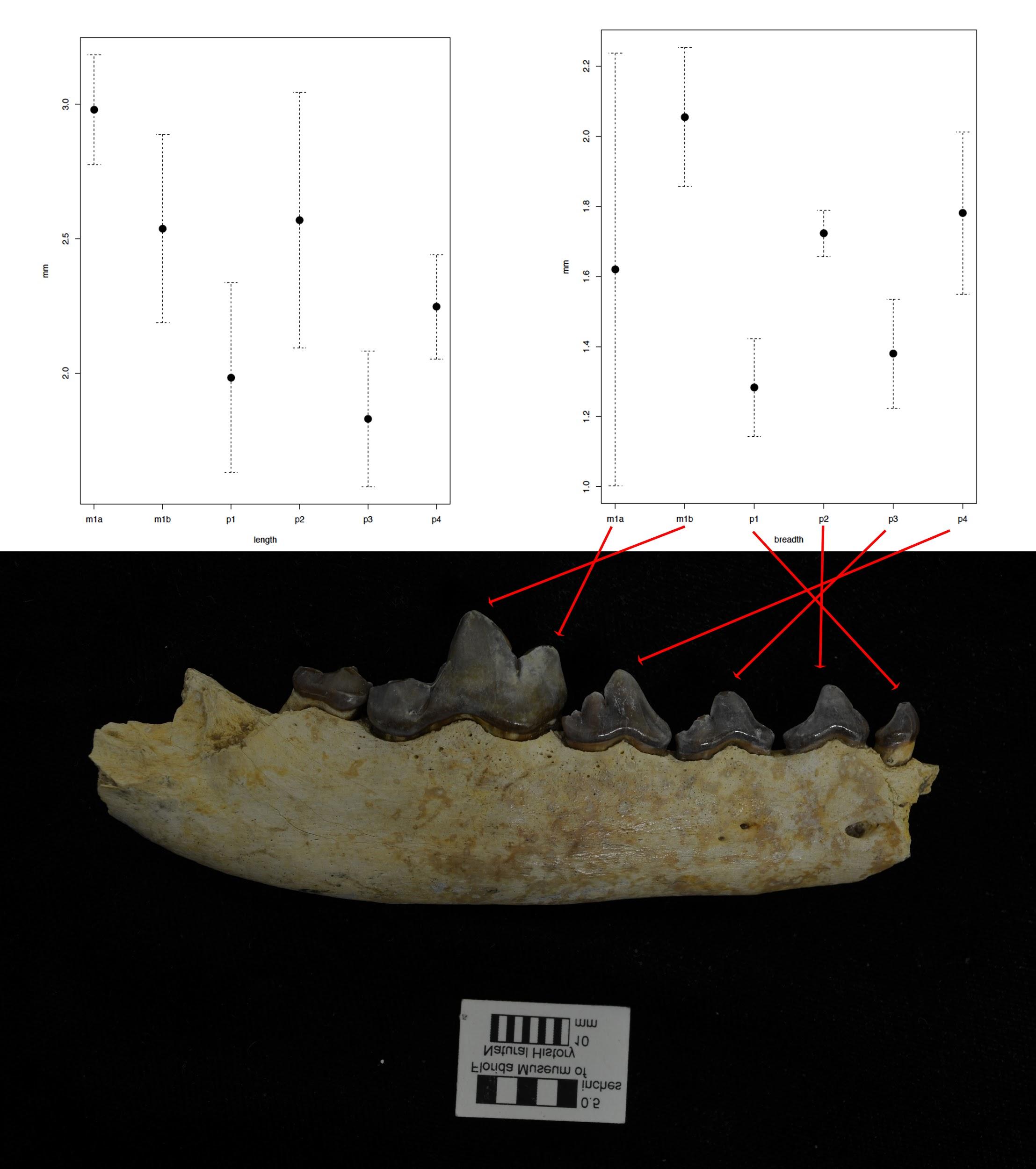


Fig S51. Canis edwardii *hemimandible found at Haile 21A. The dimensions (length and breadth) of the tooth marks made on the clay plaque according to each tooth type are shown, together with their confidence intervals. Arrows point to specific teeth and the dimensions of their resulting tooth marks.*


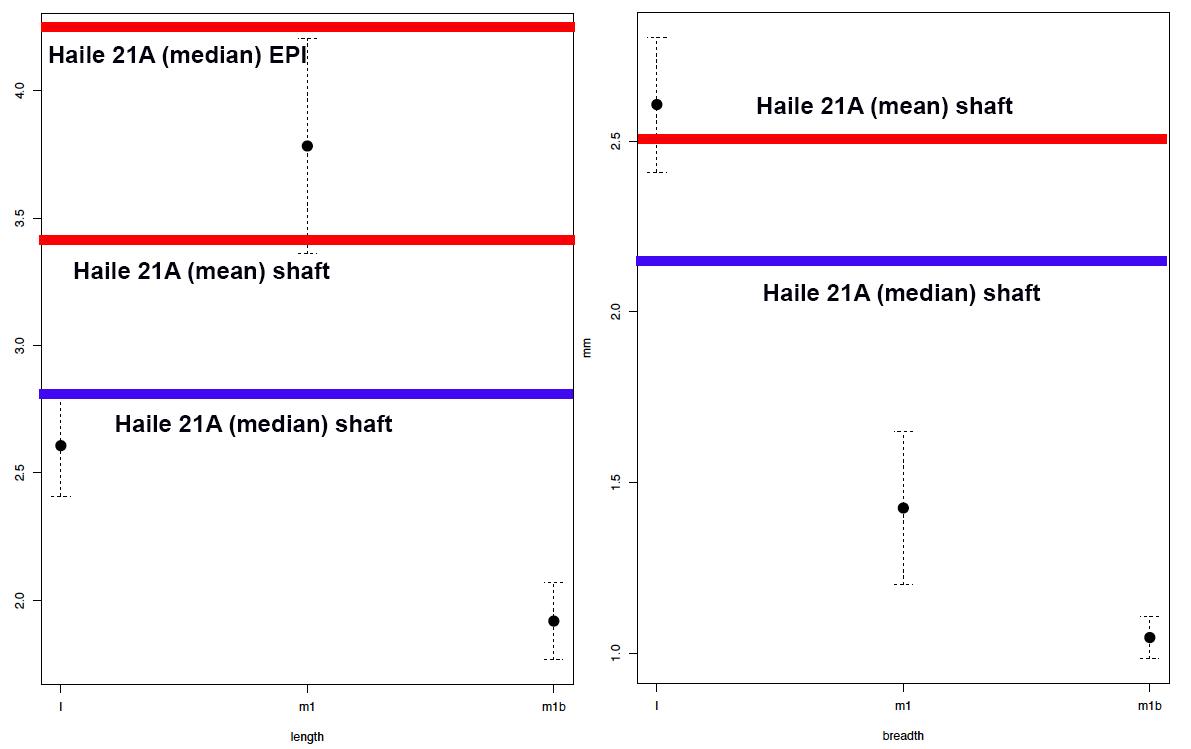


*Fig S52. Mean dimensions of tooth marks for* Smilodon gracilis *according to tooth type. Key: I, upper incisor; m1, mesial cusp of M1; m1b, distal cusp of M1.*

Overall, then, an analysis of tooth mark size eliminates *Canis edwardii*, a coyote-sized canid, as a source for most of the Haile 21A tooth marks. The tooth mark sizes produced by *Xenosmilus* are most consistent with the Haile 21A marks, although *Smilodon* remains a viable candidate.

Haile 21A tooth mark shapes compared to fossil carnivoran tooth mark shapes

Many of the tooth marks documented in the Haile 21A assemblage show distinctive morphological features like angular outlines and/or straight sides. While not abundant, their repeated occurrence indicates the actions of a dentition with specific morphological properties. The clay tooth impressions permit not only an examination of mark dimensions but mark shape. Some of the fossil carnivoran teeth do, in fact, produce very distinctive features. For *Xenosmilus*, the lower incisors and lower canine generate mostly oval-shaped and half-circle or semi-lunar-shaped tooth marks, while the upper incisors produce more irregularly shaped tooth marks (Figures S53 and S54). The marks imprinted by the cusp of P_4_ are angular and take the shape of a parallelogram. Examples of this mark type are present among the Haile 21A peccaries. Perhaps the most distinctive mark is produced by the *Xenosmilus* M_1_. Shallow penetration of this tooth into the clay results in a triangular mark, while deeper penetration produces a narrow striation or groove that extends from the triangle (Figure S55). The premolars of *Canis edwardii* generate oval and teardrop-shaped marks. The cusps of this carnivoran’s M_1_ create an oval mark with a curved extension (Figure S56). For *Smilodon*, there is a sharp contrast in mark morphology between the upper incisors and the carnassials. The former is rounded—much more so than those of *Xenosmilus*—while the latter is elongated and, in the case of the P^4^, highly curved (Figure S55). Asymmetrical marks like those produced by the *Smilodon* carnassials were not observed on the Haile 21A peccary remains.


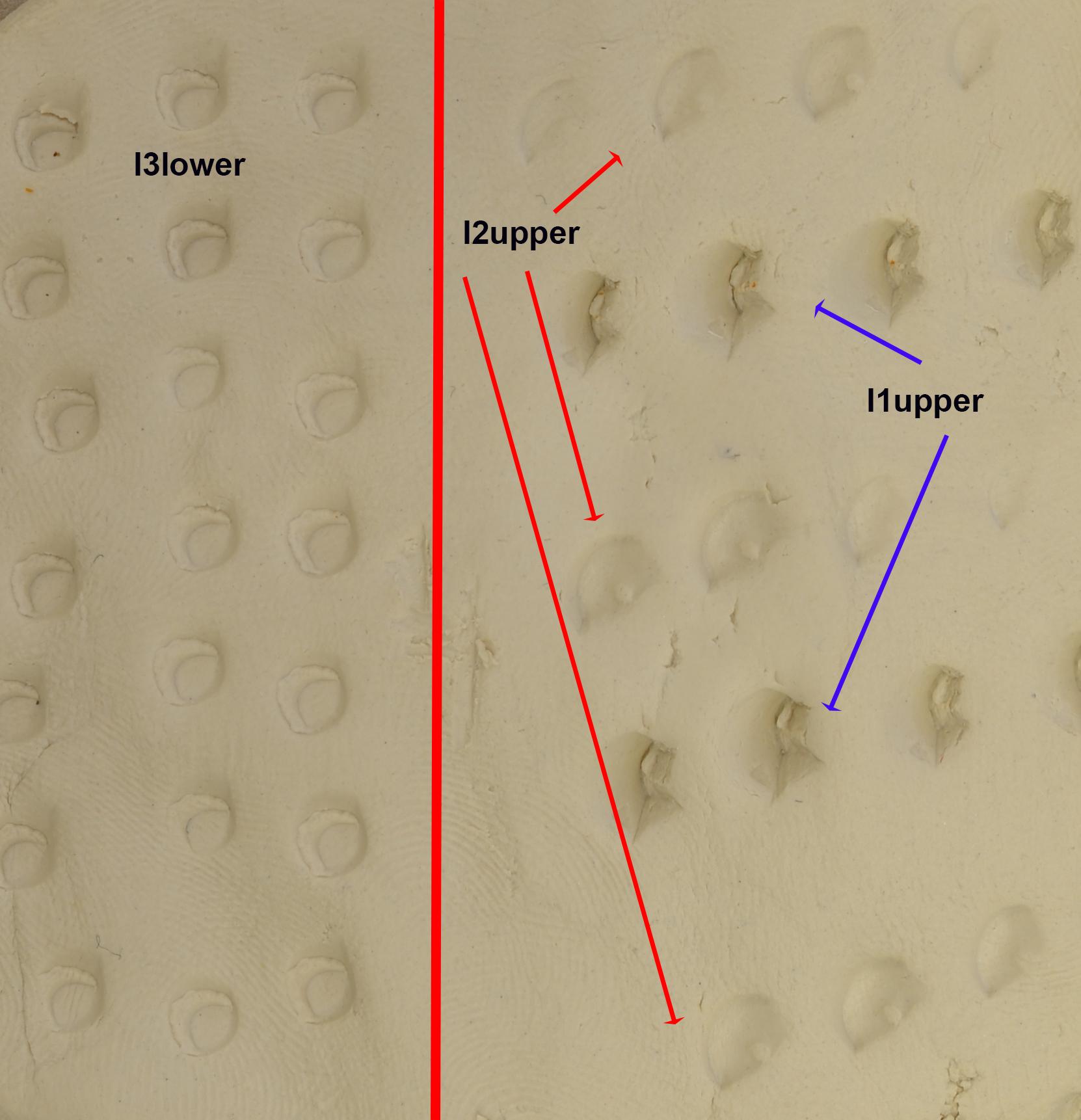


*Fig S53. Tooth marks made with lower and upper incisors of* Xenosmilus hodsonae*.*


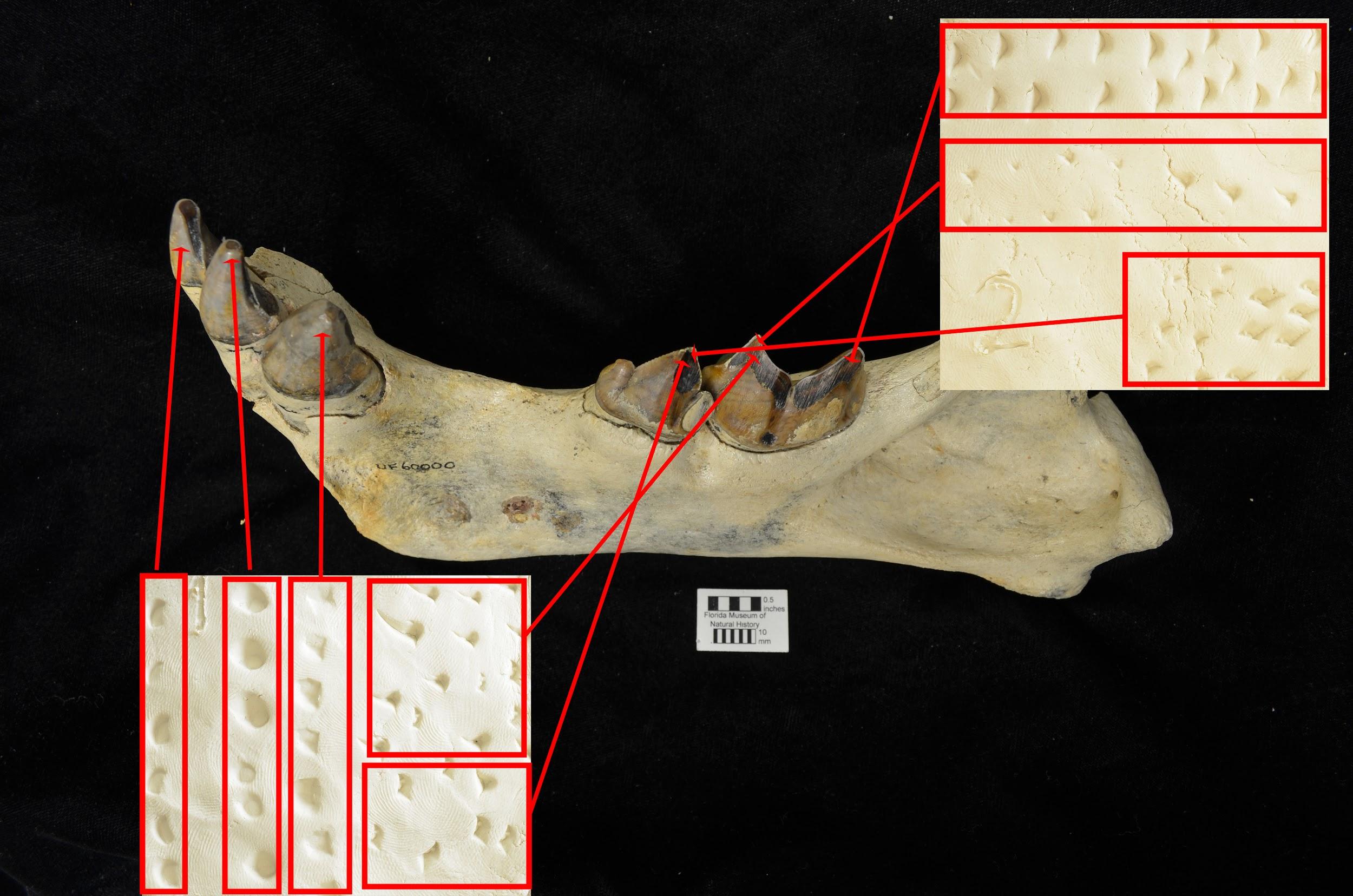


*Fig S54. Tooth mark diversity resulting from the impression of each tooth type and cusp for* Xenosmilus*. Marks were made with specimen UF 60000.*


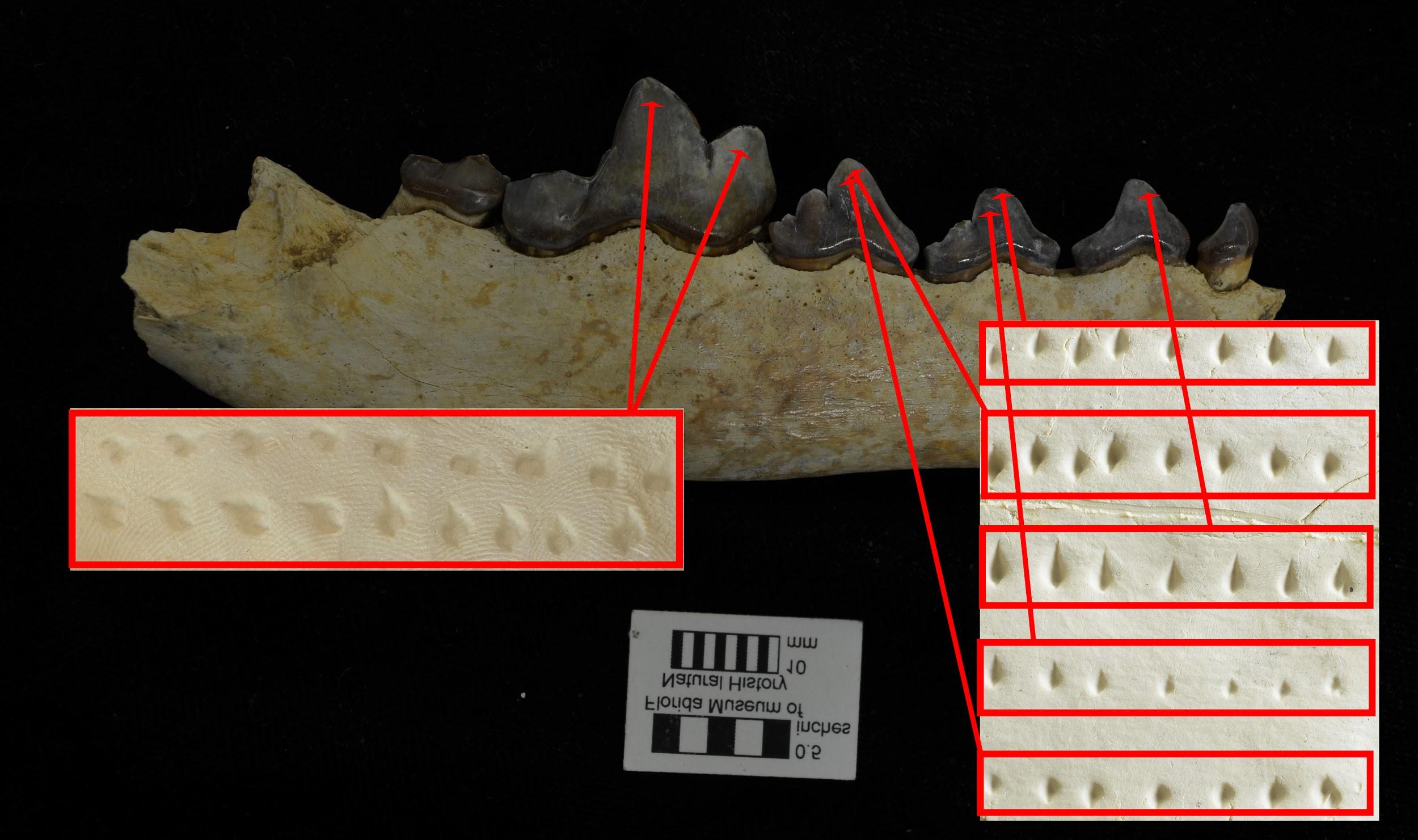


*Fig S55. Tooth mark diversity resulting from the impression of each tooth type and cusp for* Canis edwardii*. Marks were made with specimens UF 63175 and UF 221007.*


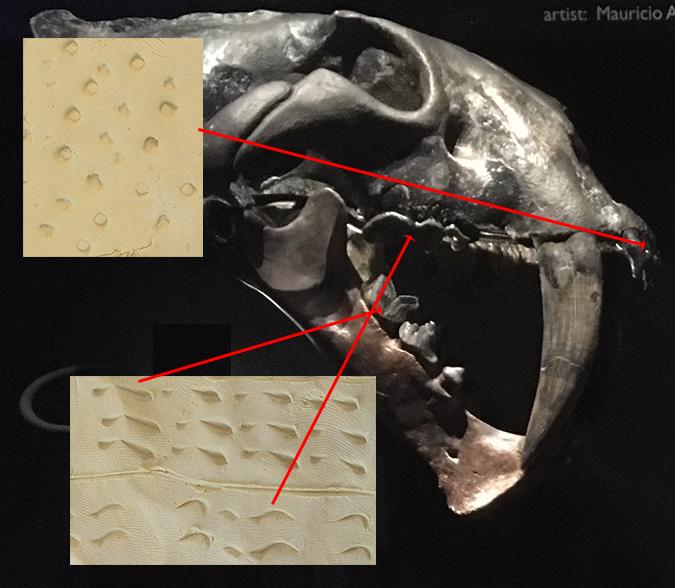


*Fig S56. Cast of* Smilodon gracilis *from FLMNH similar to the one used for making the tooth impressions in the comparative analysis. Note the divergent morphologies of the carnassial and incisor impressions.*

A handful of triangular marks appear on the Haile 21A *Platygonus* bones. Some are incipient, where only two sides of the triangle are distinctly evident due to limited compression of the cortical surface (e.g., Figure S57D), while others preserve all three sides (Figure S57C). These marks mimic closely those produced by the *Xenosmilus* M_1_ (Figure S57B), where the mesial and distal cusps, along with the intercuspid notch, simultaneously impact the cortical surface (Figure S57A). Several other triangular marks from the Haile 21A assemblage are shown in Figure S58. The distinctive morphology of the *Xenosmilus* carnassial may also be responsible for several triangular marks with emanating striae on the Haile 21A remains (Figure S59). Several semi-lunar tooth marks on the Haile 21A remains are similar to those produced by the *Xenosmilus* incisors, especially the I^2^ (Figure S60).


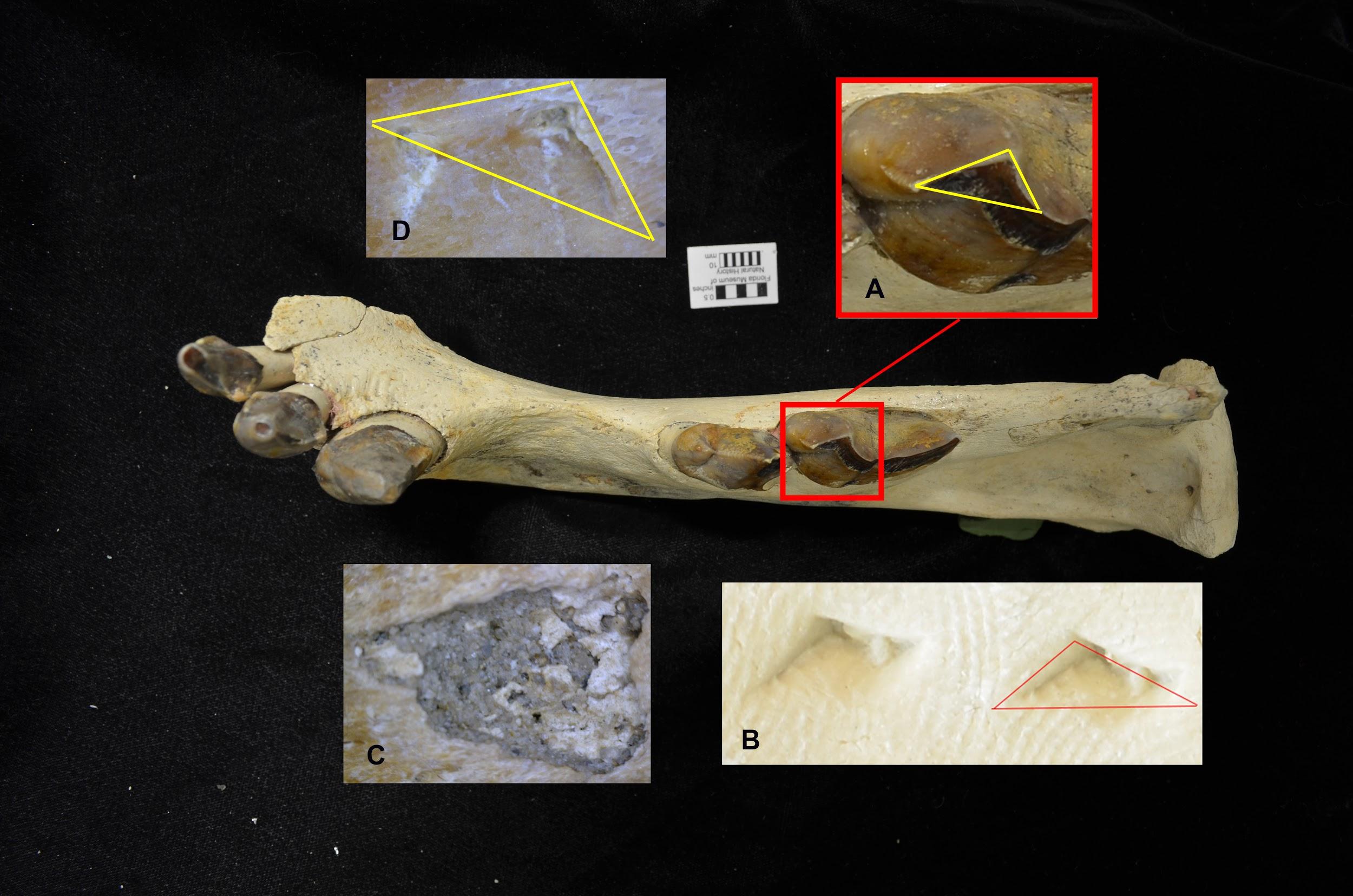


*Fig S57.* Xenosmilus *mandible. Notice the triangular shape of the crests of the mesial cusp of the carnassial (A). These generate a triangular shape on the marks reproduced on the clay plaques (B). Similar marks were documented on the* Platygonus *fauna (C, D).*


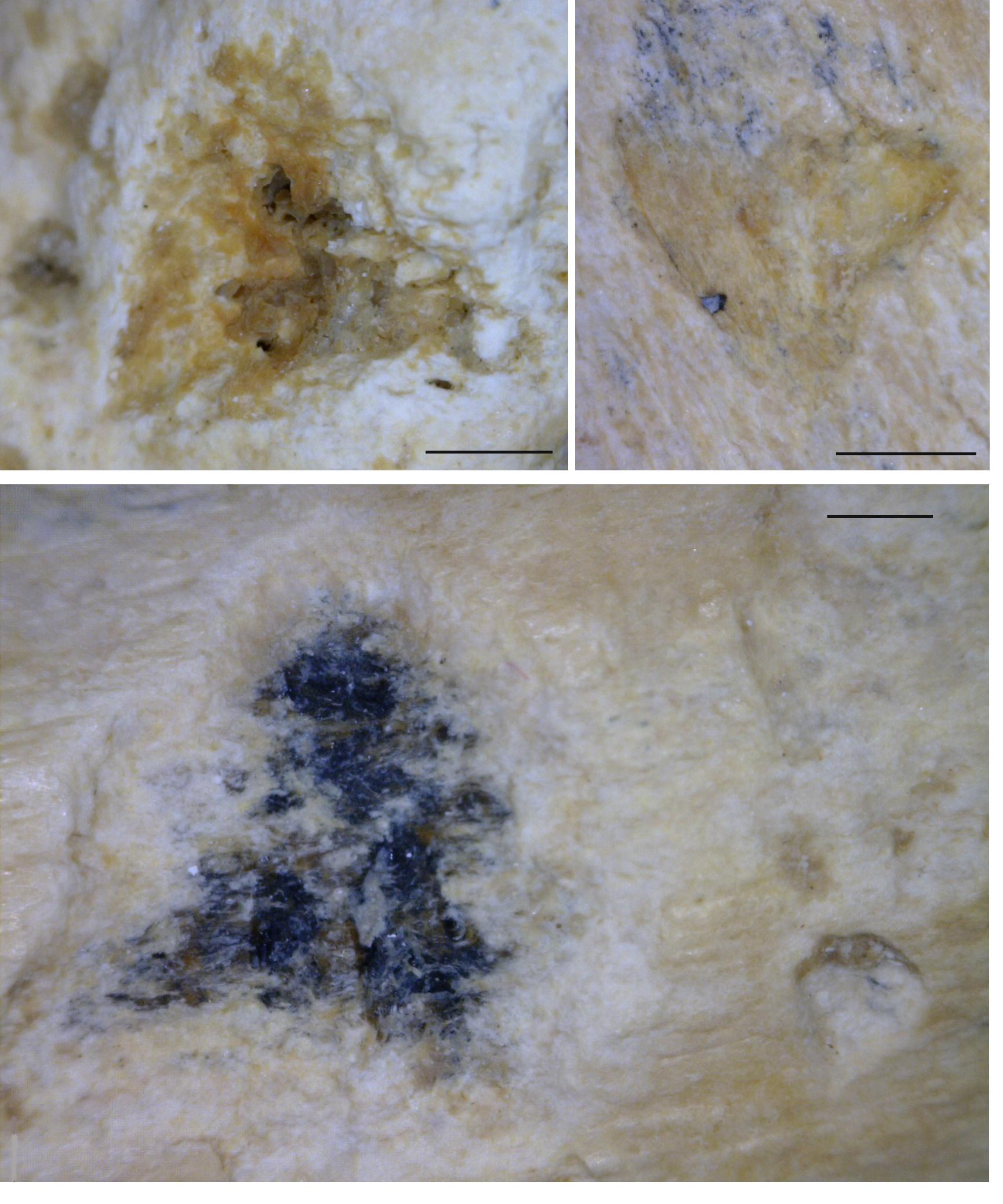


*Fig S58. Typical triangular marks found on the* Platygonus *bones from Haile 21A. Scale=1mm*


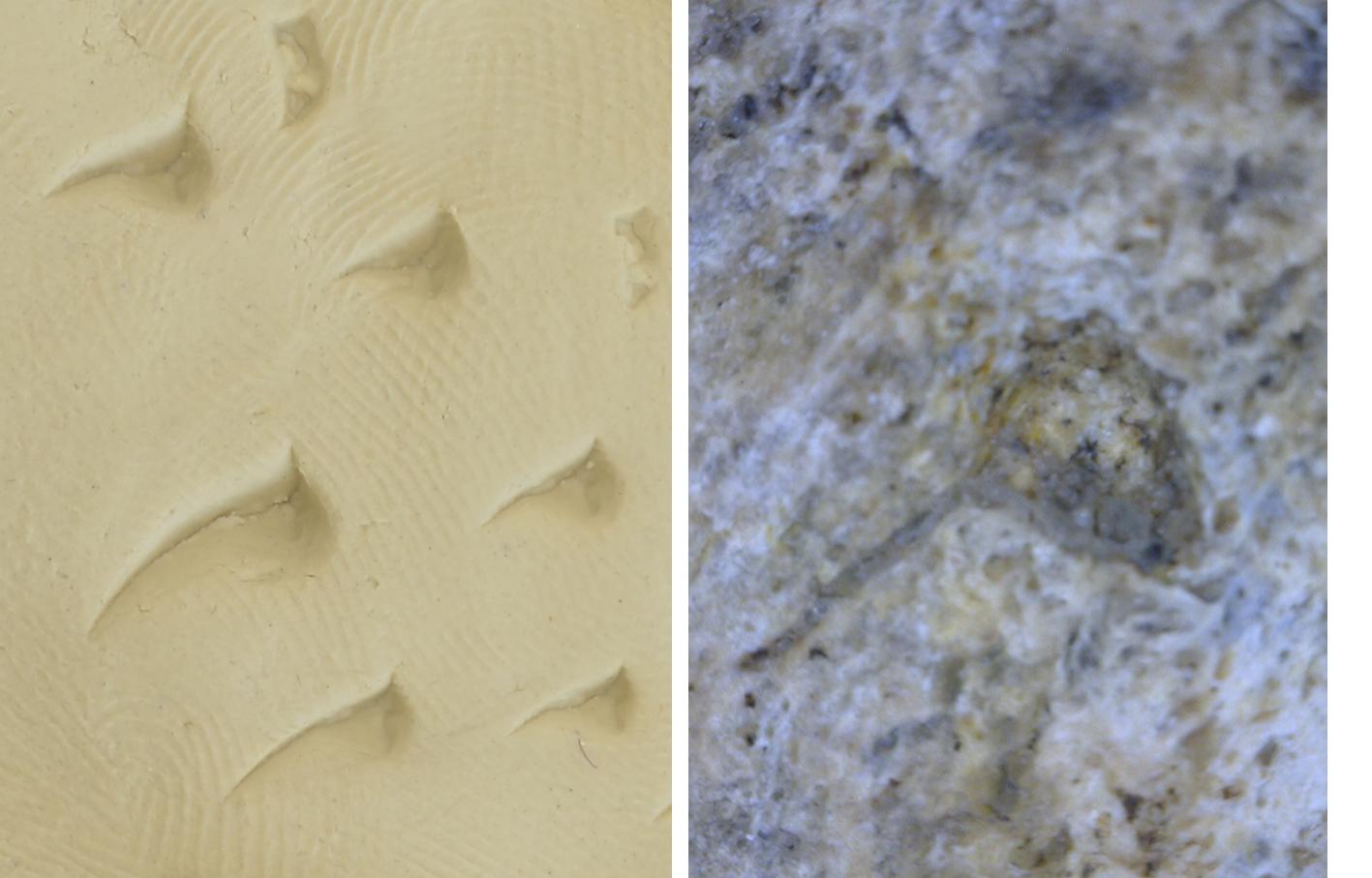


*Fig S59. Kite-shaped mark documented on some of the* Platygonus *specimens (right) and approximately reproduced on artificial tooth impressions with the* Xenosmilus *carnassial (left).*


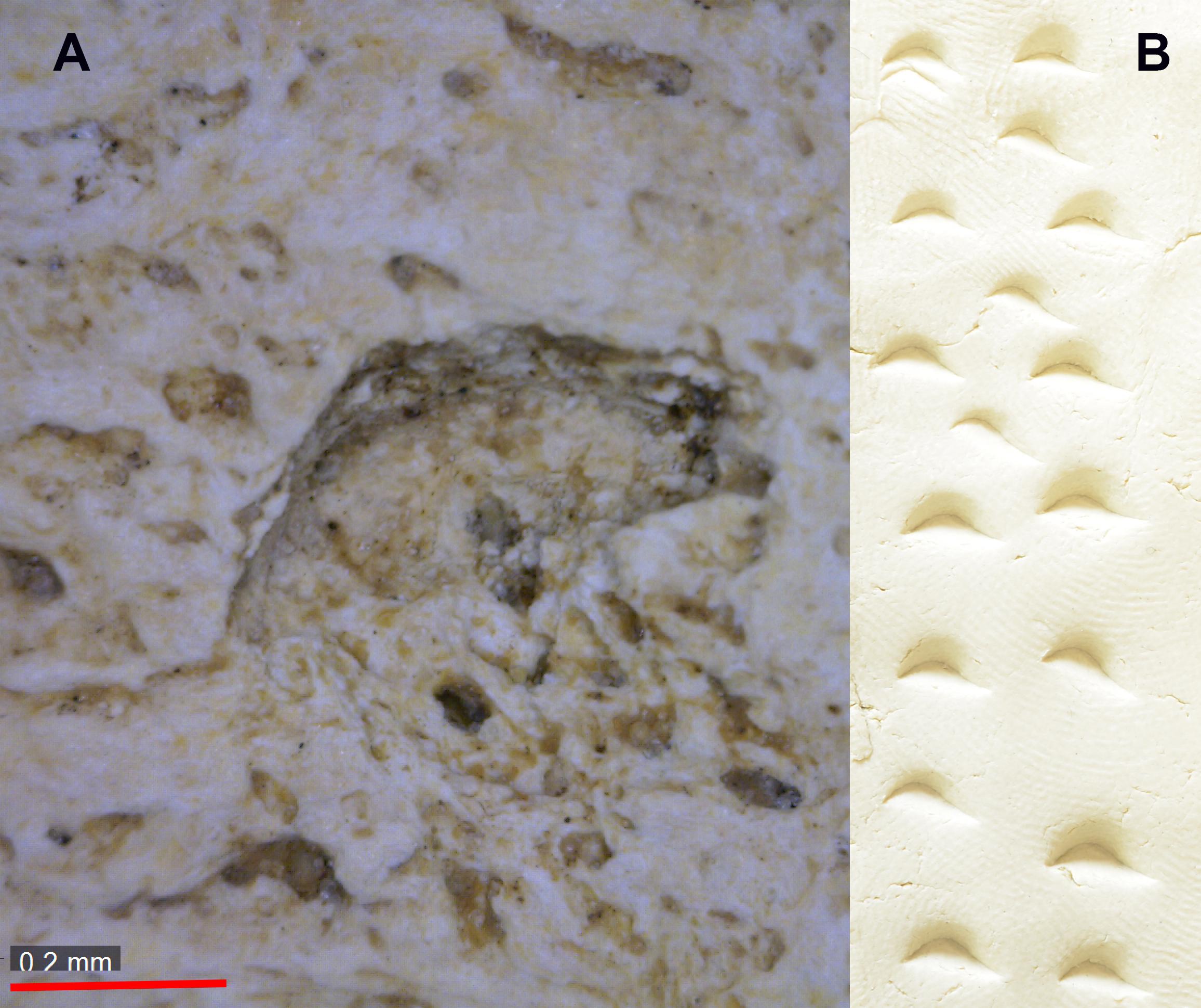


*Fig S60. Semi-lunar tooth mark documented on several* Platygonus *specimens (left) and reproduced tooth marks using the* Xenosmilus *second incisors* (right)*.*

Of the distinctive tooth mark shapes produced by the dentitions of the three fossil carnivorans, only those associated with *Xenosmilus* appear among the Haile 21A sample. That most of the Haile 21A marks are circular or oval in plan view indicates that tooth-on-bone contact occurred largely via the anterior dentition. Given the size profile of the Haile 21A tooth marks, that anterior dentition most likely belonged to *Xenosmilus*.

**The taphonomic history of the Haile 21A peccary assemblage**

*Accumulation of the peccary assemblage*

Simms [9] identifies three general bone-accumulating mechanisms in fissures, sinkholes, and caves: (1) abiotic allochthonous, where remains were introduced by an abiotic agency, (2) biotic autochthonous, where bones represent the remains of animals who lived and died within the feature itself, and (3) biotic allochthonous, where remains were transported in by a biotic agency [see also 10,11: 47,12]. Within this framework, we consider several hypotheses for the origin of the Haile 21A peccary assemblage.

Abiotic allochthonous

The first possibility under this scenario is that the peccaries, after dying and decaying on the landscape, were incorporated incidentally into the sinkhole as colluvium. There is no evidence for the scratches, striae, or rounding that typically result from the incidental contact of bone surfaces with sedimentary particles during fluvial transport or soil mass wasting. Weathering profiles indicate that most of the remains were not exposed for an extended period to UV radiation, freeze-thaw and wet-dry cycles, thermal fluctuations, or other environmental factors known to result in subaerial damage. This all suggests that the peccaries’ eventual entry into the Haile 21A sinkhole did not involve the long-distance and/or turbulent, piecemeal transport of previously exposed skeletal elements. While we consider it unlikely, repeated short distance abiotic movement of complete carcasses from within the immediate vicinity of the sinkhole cannot be ruled out.

It is also possible that the peccaries ventured too close to the Haile 21A fissure and perished after an accidental fall. The geomorphology of Haile 21A is indeed similar to middle and late Cenozoic sites from Florida [13,14] and elsewhere [15–17] that are interpreted as such “natural traps.” No actualistic framework exists to establish a “typical” taphonomic pattern for this type of site (if such a thing exists). [Pokines et al. [18] report taphonomic data on a faunal assemblage from an active sinkhole in Jordan. Evidence for the intentional disposal of carcasses by humans indicates that the site does not represent a natural trap, at least as we use the term here.] Nevertheless, the skeletal representation, taxonomic composition, and taphonomic characteristics of the Haile 21A fossil assemblage do not conform to expectations established for inferred natural traps. We note, for example, that only the peccaries are represented by all skeletal elements, which contrasts markedly with the piecemeal and highly biased representation of the other Haile 21A macrofauna (except *Xenosmilus* and, to a lesser extent, *C. edwardii*). What is more, the expectation is that fossil assemblages from natural traps sample taxa in proportion to their representation on the local landscape. As Table S11 shows, peccaries make up, on average, ~25% of the cataloged ungulates + sloths among sinkhole fossil assemblages in Florida. At 96.4%, this number for Haile 21A is a clear outlier. To be sure, monospecific traps are not unknown, but even in these circumstances a case can be made that the assemblage samples macrofauna proportional to their local abundance [e.g., ibex-dominated traps in rugged, mountainous terrain; 19]. Because we see no compelling reason to believe that peccaries either were considerably more (indeed almost uniquely) susceptible than any of the other macroherbivores to entrapment within the sinkhole [such an explanation more readily applies to carnivorans and primates who, because of curiosity, climbing aptitude, and/or attraction to decaying carcasses, are expected to be more likely to enter—albeit intentionally rather than accidentally—underground features like sinkholes [17,20,21]] or made up ~95% (or ~90% if measured by MNI) of the local macroherbivore population around Haile 21A, these patterns of skeletal part and taxonomic representation are inconsistent with a natural trap scenario.

Skeletons in natural traps also tend to exhibit little or no carnivoran damage. This is largely because carcasses are insulated from many terrestrial taphonomic processes, including carnivoran feeding. Another reason is that individual carcass deposition often occurs diachronically rather than simultaneously [22,23]. So, even when carnivorans—usually drawn by the promise of an easy meal—enter and become trapped within the chamber, they consume and potentially damage only the carcass(es) that retain(s) edible tissue. Carcasses introduced either long before a carnivoran enters, or after it succumbs to starvation, remain unmodified. Tooth mark rates among the Haile 21A peccaries are not extraordinary, but at ~8% are far higher than those documented among inferred natural trap assemblages [16,17,19,22,24,25]. Tooth marks and furrowing also occur across most skeletal elements and on multiple individuals. This indicates that most if not all the peccary carcasses were accessible and of nutritive value. Unless we assume the Haile 21A carnivorans—and the two *Xenosmilus* individuals in particular—survived entrapment for the months if not years required to produce a cumulative palimpsest of dozens of peccary carcasses, the frequency and extent of carnivoran damage is also inconsistent with a natural trap scenario. Mortality profiles are employed to identify natural traps as well, usually under the assumption that a fissure captures individuals proportional to their frequency in a living population [e.g., 24: 777]. However, many inferred natural traps do not display the expected “catastrophic” or “L-shaped” profile, and such departures can be explained by the behavioral inclinations of different age groups [19,26]. Mortality profiles are therefore of dubious utility in the discrimination of natural trap assemblages. While a natural trap hypothesis cannot be refuted definitively, we think it is improbable.

Table S11. Relative abundance of peccary remains among middle and late Cenozoic sites in Florida.

| **Locality** | **Age** | **Sinkhole (Y/N)** | **NISP ungulates + sloths** | **NISP peccaries** | **%NISP peccaries** |
| --- | --- | --- | --- | --- | --- |
| Tyner Farm | Late Miocene | Y | 953 | 1 | 0.1% |
| Haile 16A | Early Pleistocene | Y | 335 | 0 | 0.0% |
| Haile 21A | Early Pleistocene | Y | 1652 | 1592 | 96.4% |
| Inglis 1A | Early Pleistocene | Y | 3161 | 353 | 11.2% |
| Inglis 1C | Early Pleistocene | Y | 131 | 10 | 7.6% |
| Coleman 2A | Middle Pleistocene | Y | 257 | 84 | 32.7% |
| Arredondo | Late Pleistocene | Y | 63 | 5 | 7.9% |
| Cutler Hammock | Late Pleistocene | Y | 828 | 457 | 55.2% |
| Haile 8A | Late Pleistocene | Y | 180 | 22 | 12.2% |
| Reddick | Late Pleistocene | Y | 241 | 58 | 24.1% |
| Love Bone Bed | Late Miocene | N | 17514 | 451 | 2.6% |
| Moss Acres | Late Miocene | N | 331 | 1 | 0.3% |
| Leisey Shell Pit 1A | Early Pleistocene | N | 14262 | 863 | 6.1% |
| Withlacoochee River 1A | Early Pleistocene | N | 235 | 28 | 11.9% |
| La Bell Highway Pit | Middle Pleistocene | N | 1390 | 50 | 3.6% |
| Tri-Britton | Middle Pleistocene | N | 654 | 22 | 3.4% |
| Aucilla 1B | Late Pleistocene | N | 597 | 0 | 0.0% |
| Hornsby Springs | Late Pleistocene | N | 129 | 0 | 0.0% |
| Ichetucknee River | Late Pleistocene | N | 808 | 15 | 1.9% |
| Melbourne | Late Pleistocene | N | 901 | 170 | 18.9% |
| Millennium Park | Late Pleistocene | N | 392 | 7 | 1.8% |
| Peace River | Late Pleistocene | N | 822 | 11 | 1.3% |
| Rainbow River | Late Pleistocene | N | 385 | 1 | 0.3% |
| Santa Fe River 2 & 3 | Late Pleistocene | N | 592 | 6 | 1.0% |
| Seminole Field | Late Pleistocene | N | 1588 | 181 | 11.4% |
| Vero | Late Pleistocene | N | 717 | 19 | 2.6% |
| Waccasassa River | Late Pleistocene | N | 2193 | 109 | 5.0% |

Biotic autochthonous

Extant peccaries are known to shelter temporarily in caves to avoid harsh weather and extreme temperatures [27,28]. It appears that extinct peccaries like *Platygonus* also recurrently entered caves to farrow or when sick, injured, or aged [29,30]. Such behavior would explain the presence of complete, partially articulated skeletons, and the preservation in the Haile 21A assemblage of a handful of dentitions and post-crania from individuals <1 year in age is consistent with farrowing. On the other hand, the steeply oriented—in fact nearly vertical—walls of the portion of the sinkhole excavated in 1983-1984 probably would have precluded ready access by these ungulates. It is important to realize, however, that the Florida platform has experienced ca. 30 to 60 meters of isostatic uplift over the last one to 1.5 million years [31,32], and concomitant erosion likely removed the topmost portion of the Haile 21A dissolution feature. It is therefore unknown whether a more benign entrance existed in the early Pleistocene.

Biotic allochthonous

The Haile 21A macroherbivore assemblage is essentially monospecific, and only the dominant taxon (peccary) preserves carnivoran damage. These observations strongly hint at a specialized biotic bone-collecting agent. We acknowledge that nutritive phase bone damage unequivocally linking carnivorans to the consumption of most of the peccary carcasses does not necessarily imply a carnivoran role in their accumulation. However, the tooth mark documented on the atlas vertebra is relevant here, as it may reflect behavior analogous to modern lions, who grip the nape during hunts and/or while dragging kills to a feeding area [33, see also 34: 19]. It is plausible, then, that a predator (which we identify as a large felid; see below) killed the peccaries somewhere in the vicinity and transported them back near or directly into the cavity for consumption. It is interesting to note too that the Cutler Hammock site, which is the only other assemblage from Table S11 that even approaches the relative frequency of peccaries seen at Haile 21A, also preserves evidence for carnivoran feeding damage along with the remains of several carnivoran individuals (dire wolves, in this case) [35]. We might appeal to the age structure of the peccaries here, but carnivorans, like natural traps, produce a variety of mortality profiles. Lions, for instance, shift their preferences based on season, habitat type, and the demographic characteristics of the prey population [36]. This makes it difficult to distinguish one carnivoran from another much less a biotic process like predation from an abiotic one like a natural trap.

Synthesis

The original morphology of the Haile 21A fissure remains uncertain and, given the nature of the excavation (a rescue operation within an active limestone mine), we lack the geological information required to establish the rate of accumulation of the peccary carcasses and their precise spatial relationship with the carnivoran remains in general and the *Xenosmilus* skeletons in particular. The taphonomic data at hand nevertheless indicate that an abiotic allochthonous mechanism is the least likely explanation for the accumulation of the peccaries, unless it involved very short distance (i.e., a few meters) movement of carcasses previously deposited by some biotic process in the vicinity of the fissure’s opening. It is not inconceivable that these non-scansorial, and presumably gregarious, ungulates entered the cave voluntarily and either died naturally (e.g., in a winter storm) or were ambushed by a predator. What is not in doubt, however, is that carnivorans consumed many of the peccaries, and a tooth-marked atlas vertebra reveals that at least one of these individuals may have been a victim of transport if not predation. We therefore find a biotic allochthonous scenario to be the most convincing. Carnivorans, including felids, do transport carcasses over short distances to refuges like caves, rockshelters, or patches of dense vegetation in order to provision young, feed in the shade, and/or avoid would-be competitors [37–39]. While there is no clear evidence that Haile 21A was a residential or maternity den, the cavity, or its immediate surroundings, may have functioned as a feeding refuge, perhaps within a local landscape particularly favorable to serial hunting [cf. 40: 430,41: 219]. Of course, this does not preclude the operation of other biotic or even abiotic events in the accumulation of the Haile 21A peccary assemblage (e.g., non-predation deaths, farrowing), but we think such processes, if operant, were only minor contributors.

This of course leaves open the question of the origin of the remainder of the faunal assemblage, especially the two *Xenosmilus* individuals. That carnivoran damage does not occur on any of the non-peccary remains argues against their arrival via some biotic allochthonous mechanism (e.g., predator transport and consumption). With two exceptions—*Xenosmilus* and *C. edwardii*—the non-peccary taxa are also represented by only a handful of isolated specimens. The arrival of most of these animals into the Haile 21A cavity can therefore be readily attributed to the random incorporation of skeletal elements via colluvial processes. All but two of the 131 *C. edwardii* remains derive from adult individuals, so their appearance in the assemblage is likely not due to this taxon’s use of Haile 21A as a den. Both *Xenosmilus* skeletons represent adults: one prime-aged and the other older, but not geriatric. The lack of juvenile *Xenosmilus* suggests that Haile 21A likely did not function as a den for this taxon either. There is no evidence among the carnivoran remains for perimortem trauma that might accompany a violent fall into the sinkhole. Nor were the *Xenosmilus* individuals—or any other carnivoran that may have fed upon the peccary carcasses—likely to have been victims of voluntary entry and prolonged entrapment given the extent of feeding damage across the peccary skeletons. It is important to point out, too, that carnivorans are common components of modern and fossil faunal assemblages in caves, fissures, and other subterranean features that demonstrably did not serve as natural traps [37,42–44]. While the walls of the excavated portion of the Haile 21A sinkhole were indeed quite steep and, thus, a potentially significant impediment to escape, the original morphology of the entrance, as we point out above, is unknown. We also note that contemporary sinkholes throughout Florida often contain fallen trees and/or collapsed rockslides that would facilitate ingress and egress to a carnivoran, especially a felid (Hulbert, personal observations).

*The modification of the peccary assemblage*

We consider the most likely nutritive phase modifier of the Haile 21A peccary carcasses to be a large carnivoran, namely a felid or a canid. Because felids feed almost exclusively on fresh, complete carcasses and focus on flesh removal rather than within-bone nutrient extraction, there are significant differences between these two taxa in terms of carcass processing behavior [3,5,7,45–54]. Both the overall tooth mark frequency and the frequency of tooth-marked long bone specimens (humerus, radio-ulna, femur, tibia) in the Haile 21A assemblage (7.9% and 16.5%, respectively) fall below those recorded on carcasses consumed by modern lions and gray wolves at their kill sites. Gidna et al. [7: Table 3], for instance, find that wild lions feeding on small-to-medium ungulates (warthog, zebra, wildebeest) leave tooth marks on ~75% of the humeri, radio-ulnae, femora, and tibiae, while Yravedra et al. [50: Tables 1 and 2] report that wild wolves tooth-mark all anatomical regions of horse carcasses at rates between 11-80%. The Haile 21A tooth mark frequencies do however compare favorably to those recorded in a modern open-air bone assemblage (Olduvai Carnivore Site; OCS) from the eastern edge of the Serengeti thought to result from lion predation and feeding. The damage rate at OCS, which is composed largely of blue wildebeest (*Connochaetes taurinus*), is 4.5% across all skeletal elements and 20.3% for humeri, radio-ulnae, femora, and tibiae [34: Table S1I]. Damage frequencies higher than those of the Haile 21A peccary assemblage are seen among residential/maternity den accumulations of leopards [~75% of long bones preserve at least one tooth mark; 47: Table 2] and pumas (*Puma concolor*) [~47% of all bones exhibit carnivoran damage; 55: 458]. While these discrepancies may reflect real differences in feeding behavior between these modern carnivorans and the extinct species responsible for consuming the Haile 21A peccaries, it is also likely that site function and divergent taphonomic histories play a role here. For one, the formation of the modern leopard and puma den assemblages was not observed from beginning to end, so it is possible that feeding from other carnivorans inflated the resulting damage frequencies. Bone damage at residential or maternity dens can nevertheless be more extensive than that at kill or feeding sites [55: 458,56: 268]. If, as we argue above, Haile 21A functioned as a feeding refuge, we might thus expect the intensity of damage to be lower than what is observed in modern dens. Cortical surface preservation is also a potential factor. The Haile 21A fauna is in generally excellent condition but subaerial weathering, microbial activity, and/or manganese/carbonate formation do affect some of the specimens, any one of which may obscure, or at least render less confidently identifiable, an unknown number of genuine tooth marks. Important, too, is that damage readily attributable to carnivoran furrowing in actualistic studies is much less so in fossil contexts. This is due to the difficulty of identifying in fossil assemblages nutritive phase breakage on those bones or bone portions where furrowing is most likely to occur (i.e., those with extensive trabeculae). Unlike many actualistic studies, the Haile 21A tooth mark frequencies do not include specimens with furrowing damage (importantly, the OCS damage frequencies also omit furrowing). If anything, then, the frequencies from Haile 21A underestimate the true extent of carnivoran damage. Finally, while feeding damage is spread across many of the 69 peccaries documented at the site, it is possible that some of those animals were never subjected to carnivoran feeding, which would also contribute to depressed tooth mark frequencies relative to those seen among actualistic studies where all carcasses were consumed.

More telling than the frequency of tooth-marked specimens, we think, is the number of tooth marks *per specimen*. The creation of >10 tooth marks on a single specimen is much more common among canids than it is among big cats. Of the long bones from a sample of Size Class 2 animals killed and consumed by wild leopards, only 2.3% preserve >10 tooth marks [47: Table 2]. In contrast, the wild wolves studied by Yravedra et al. [50] created >10 tooth marks on 21.8% of the long bones from eight horse carcasses. Even a diminutive canid like the red fox (*Vulpes vulpes*) creates >10 tooth marks on 21.4% of the long bones of Size Class 2 carcasses [51: Table 1]. Not a single specimen from Haile 21A preserves >10 tooth marks, and only seven, all of them femora, preserve between six and ten. Gray wolves also comminute long bones or, more commonly, produce cylinders as they destroy long bone epiphyses. Both behaviors are rare among big cats and smaller canids. While nutritive phase breakage is evident on the Haile 21A long bones, none of it resulted in shaft fragmentation, and only a handful of long bone cylinders occur. Those long bones that are missing epiphyses also lack the crenulated edges that result from sustained gnawing. The absence of gastric etching on compact bones is also inconsistent with the feeding behavior of durophagous carnivorans, who regularly break and consume these bones either intentionally to extract within-bone nutrients or incidentally as they feed on the lower limbs [43,54,57]. This all makes it unlikely that the Haile 21A bone modifier was a wolf-sized canid like *Canis armbrusteri*. The other canid that is represented in the fossil assemblage, *Canis edwardii*, is approximately the size of a modern coyote (*Canis latrans*). We know surprisingly little about the carcass processing behavior of coyotes, so it is unclear how its similarly sized extinct counterpart might have damaged the skeleton of a Size Class 2 animal like *Platygonus*. It seems reasonable to assume that coyotes and, by extension, *C. edwardii*, generate(d) more damage than foxes and less damage than wolves [cf. 58]—a characterization that matches in some ways the Haile 21 faunal assemblage. The size distribution of the Haile 21A tooth marks, however, indicates that *C. edwardii* was not a significant factor in the modification of the peccary carcasses. This leaves the two large felids, *Xenosmilus* and *Smilodon*, as the most likely candidates. Unfortunately, we lack the spatial and geological data required to fully evaluate the contemporaneity of either taxon with the peccary remains [23,e.g., 59], but the shape and size of the fossil tooth marks are more consistent with *Xenosmilus*. While we do not dismiss the possibility of multiple taphonomic actors (e.g., some gnawing of bones by small canids), the weight of the evidence—including patterns of breakage and the frequency, anatomical patterning, dimensions, and shape of tooth marks—points to *Xenosmilus* as the principal source of nutritive phase damage on the Haile 21A peccary carcasses. Because felids tend to access and (if necessary) transport complete, freshly killed carcasses [33,60,61], and the fact that even leopards are capable of consuming the vertebrae of prey the size of *Platygonus* [49], this identification also reinforces our impression from the skeletal part data that complete peccary skeletons were originally deposited in or around the sinkhole.

**References**

[1] L.D. Martin, J.P. Babiarz, V.L. Naples, The osteology of a cookie-cutter cat, *Xenosmilus hodsonae*, in: V.L. Naples, L.D. Martin, J.P. Babiarz (Eds.), The Other Saber-Tooths: Scimitar-Tooth Cats of the Western Hemisphere, Johns Hopkins University Press, Baltimore, 2011: pp. 43–97.

[2] T.R. Pickering, C.W. Marean, M. Domı́nguez-Rodrigo, Importance of limb bone shaft fragments in zooarchaeology: a response to “On in situ attrition and vertebrate body part profiles” (2002), by M.C. Stiner, Journal of Archaeological Science. 30 (2003) 1469–1482. https://doi.org/10.1016/S0305-4403(03)00042-6.

[3] J.A. Parkinson, T. Plummer, A. Hartstone-Rose, Characterizing felid tooth marking and gross bone damage patterns using GIS image analysis: an experimental feeding study with large felids, Journal of Human Evolution. 80 (2015) 114–134. https://doi.org/10.1016/j.jhevol.2014.10.011.

[4] M.E. Prendergast, M. Domínguez-Rodrigo, Taphonomic analyses of a hyena den and a natural-death assemblage near Lake Eyasi (Tanzania), Journal of Taphonomy. 6 (2008) 301–335.

[5] J.A. Parkinson, T.W. Plummer, R. Bose, A GIS-based approach to documenting large canid damage to bones, Palaeogeography, Palaeoclimatology, Palaeoecology. 409 (2014) 57–71. https://doi.org/10.1016/j.palaeo.2014.04.019.

[6] A. Gidna, J. Yravedra, M. Domínguez-Rodrigo, A cautionary note on the use of captive carnivores to model wild predator behavior: a comparison of bone modification patterns on long bones by captive and wild lions, Journal of Archaeological Science. 40 (2013) 1903–1910. https://doi.org/10.1016/j.jas.2012.11.023.

[7] A.O. Gidna, B. Kisui, A.Z.P. Mabulla, C. Musiba, M. Domínguez-Rodrigo, An ecological neo-taphonomic study of carcass consumption by lions in Tarangire National Park (Tanzania) and its relevance for human evolutionary biology, Quaternary International. 322–323 (2014) 167–180. https://doi.org/10.1016/j.quaint.2013.08.059.

[8] M. Andrés, A.O. Gidna, J. Yravedra, M. Domínguez-Rodrigo, A study of dimensional differences of tooth marks (pits and scores) on bones modified by small and large carnivores, Archaeol Anthropol Sci. 4 (2012) 209–219. https://doi.org/10.1007/s12520-012-0093-4.

[9] M.J. Simms, Emplacement and preservation of vertebrates in caves and fissures, Zoological Journal of the Linnean Society. 112 (1994) 261–283.

[10] E.L. Lundelius, Cave site contributions to vertebrate history, Alcheringa. 30 (2006) 195–210.

[11] E.S. Vrba, The Kromdraai australopithecine site revisited in 1980: recent investigations and results, Annals of the Transvaal Museum. 33 (1981) 17–60.

[12] B.W. Schubert, J.I. Mead, Paleontology of caves, in: W.B. White, D.C. Culver (Eds.), Encyclopedia of Caves, Elsevier, Amsterdam, 2012: pp. 590–598.

[13] A.E. Pratt, Taphonomy of the large vertebrate fauna from the Thomas Farm Locality (Miocene, Hemingfordian), Gilchrist County, Florida, Bulletin of the Florida Museum of Natural History. 35 (1990) 35–130.

[14] D.R. Ruez, Jr., Mammalian taphonomy of the Early Irvingtonian (Late Pliocene) Inglis 1C fauna (Citrus County, Florida), Southeastern Geology. 41 (2002) 159–168.

[15] L.D. Martin, B.M. Gilbert, Excavations at Natural Trap Cave, Transactions of the Nebraska Academy of Sciences. 6 (1978) 107–116.

[16] J.S. Oliver, Analogues and site context: bone damages from Shield Trap Cave {24CB91), Carbon County, Montana, U.S.A., in: R. Bonnichsen, M.H. Sorg (Eds.), Bone Modification, Center for the Study of the First Americans, Orono, Maine, 1989: pp. 73–98.

[17] M.S. Domingo, M.T. Alberdi, B. Azanza, P.G. Silva, J. Morales, Origin of an assemblage massively dominated by carnivorans from the Miocene of Spain, PLoS ONE. 8 (2013) e63046. https://doi.org/10.1371/journal.pone.0063046.

[18] J.T. Pokines, A. Nowell, M.S. Bisson, C.E. Cordova, C.J.H. Ames, The functioning of a natural faunal trap in a semi-arid environment: preliminary investigations of WZM-1, a limestone sinkhole site near Wadi Zarqa Ma’in, Hashemite Kingdom of Jordan, Journal of Taphonomy. 9 (2011) 89–115.

[19] V. Sauqué, R. García-González, R. Rabal-Garcés, J. Galán, C. Núñez-Lahuerta, M. Gisbert, G. Cuenca-Bescós, Los Batanes: a trap for the Pyrenean wild goat during the Late Pleistocene (Spain), Quaternary International. 481 (2018) 75–90. https://doi.org/10.1016/j.quaint.2017.09.011.

[20] T.R. Pickering, R.J. Clarke, J.L. Heaton, The context of Stw 573, an early hominid skull and skeleton from Sterkfontein Member 2: taphonomy and paleoenvironment, Journal of Human Evolution. 46 (2004) 277–295. https://doi.org/10.1016/j.jhevol.2003.12.001.

[21] C. Nel, J. Bradfield, M. Lombard, A. Val, Taphonomic study of a modern baboon sleeping site at Misgrot, South Africa: implications for large-bodied primate taphonomy in karstic deposits, J Paleo Arch. 4 (2021) 4. https://doi.org/10.1007/s41982-021-00080-x.

[22] A. Hubbe, A. Auler, A large Cervidae Holocene accumulation in Eastern Brazil: an example of extreme taphonomical control in a cave environment, IJS. 41 (2012) 297–305. https://doi.org/10.5038/1827-806X.41.2.15.

[23] D.M. Martín-Perea, L.A. Courtenay, M.S. Domingo, J. Morales, Application of artificially intelligent systems for the identification of discrete fossiliferous levels, PeerJ. 8 (2020) 1–25. https://doi.org/10.7717/peerj.8767.

[24] O. Marder, R. Yeshurun, R. Lupu, G. Bar-Oz, M. Belmaker, N. Porat, H. Ron, A. Frumkin, Mammal remains at Rantis Cave, Israel, and Middle-Late Pleistocene human subsistence and ecology in the Southern Levant, J. Quaternary Sci. 26 (2011) 769–780. https://doi.org/10.1002/jqs.1501.

[25] J. Castaños, P. Castaños, A. Suárez-Bilbao, M.-J. Iriarte-Chiapusso, A. Arrizabalaga, X. Murelaga, A large mammal assemblage during MIS 5c: Artazu VII (Arrasate, northern Iberian Peninsula), Historical Biology. 31 (2019) 731–747. https://doi.org/10.1080/08912963.2017.1389923.

[26] X. Wang, L.D. Martin, Late Pleistocene paleoecology and large mammal taphonomy, Natural Trap Cave, Wyoming, National Geographic Research and Exploration. 9 (1993) 422–435.

[27] W.J. Bigler, Seasonal movements and activity patterns of the collared peccary, Journal of Mammalogy. 55 (1974) 851–855. https://doi.org/10.2307/1379419.

[28] B.J. Neal, A contribution on the life history of the collared peccary in Arizona, American Midland Naturalist. 61 (1959) 177–190. https://doi.org/10.2307/2422349.

[29] A.L. Woodruff, B.W. Schubert, Seasonal denning behavior and population dynamics of the late Pleistocene peccary *Platygonus compressus* (Artiodactyla: Tayassuidae) from Bat Cave, Missouri, PeerJ. 7 (2019) 1–18. https://doi.org/10.7717/peerj.7161.

[30] K.M. Wilson, M.G. Hill, Synthesis and assessment of the flat-headed peccary record in North America, Quaternary Science Reviews. 248 (2020) 106601. https://doi.org/10.1016/j.quascirev.2020.106601.

[31] H.B. Woo, M.P. Panning, P.N. Adams, A. Dutton, Karst-driven flexural isostasy in North-Central Florida, Geochem. Geophys. Geosyst. 18 (2017) 3327–3339. https://doi.org/10.1002/2017GC006934.

[32] P.N. Adams, N.D. Opdyke, J.M. Jaeger, Isostatic uplift driven by karstification and sea-level oscillation: modeling landscape evolution in north Florida, Geology. 38 (2010) 531–534. https://doi.org/10.1130/G30592.1.

[33] G.B. Schaller, The Serengeti Lion: A Study of Predator-Prey Relations, University of Chicago Press, Chicago, 1972.

[34] M.C. Arriaza, M. Domínguez-Rodrigo, J. Yravedra, E. Baquedano, Lions as bone accumulators? Paleontological and ecological implications of a modern bone assemblage from Olduvai Gorge, PLoS ONE. 11 (2016) e0153797. https://doi.org/10.1371/journal.pone.0153797.

[35] S.D. Emslie, G.S. Morgan, Taphonomy of a Late Pleistocene carnivore den, Dade County, Florida, in: D.W. Steadman, J.I. Mead (Eds.), Late Quaternary Environments and Deep History: A Tribute to Paul S. Martin, The Mammoth Site of Hot Springs Scientific Papers, Hot Springs, SD, 1995: pp. 65–83.

[36] M.C. Arriaza, M. Domínguez-Rodrigo, C. Martínez-Maza, A. Mabulla, E. Baquedano, Differential predation by age and sex classes in blue wildebeest in Serengeti: study of a modern carnivore den in Olduvai Gorge (Tanzania), PLoS ONE. 10 (2015) e0125944. https://doi.org/10.1371/journal.pone.0125944.

[37] C.K. Brain, The Hunters or the Hunted: An Introduction to African Cave Taphonomy, University of Chicago Press, Chicago, 1981.

[38] M. Domínguez-Rodrigo, Dinámica trófica, estrategias de consumo y alteraciones óseas en la sabana africana: resumen de un proyecto de investigación etoarqueológico (1991-1993), Trabajos de Prehistoria. 51 (1994) 15–37.

[39] M. Mondini, A.S. Muñoz, Pumas as taphonomic agents: a comparative analysis of actualistic studies in the Neotropics, Quaternary International. 180 (2008) 52–62. https://doi.org/10.1016/j.quaint.2007.08.013.

[40] A.K. Behrensmeyer, Taphonomy and hunting, in: M.H. Nitecki, D.V. Nitecki (Eds.), The Evolution of Human Hunting, Plenum Press, New York, 1987: pp. 423–450.

[41] G. Haynes, Mass deaths and serial predation: comparative taphonomic studies of modern large mammal death sites, Journal of Archaeological Science. 15 (1988) 219–235. https://doi.org/10.1016/0305-4403(88)90064-7.

[42] D.J. de Ruiter, L.R. Berger, Leopards as taphonomic agents in dolomitic caves—implications for bone accumulations in the hominid-bearing deposits of South Africa, Journal of Archaeological Science. 27 (2000) 665–684. https://doi.org/10.1006/jasc.1999.0470.

[43] T.R. Pickering, Reconsideration of criteria for differentiating faunal assemblages accumulated by hyenas and hominids, International Journal of Osteoarchaeology. 12 (2002) 127–141.

[44] F.M. Martín, Cueva del Milodón. The hunting grounds of the Patagonian panther, Quaternary International. 466 (2018) 212–222. https://doi.org/10.1016/j.quaint.2016.05.005.

[45] G. Haynes, A guide for differentiating mammalian carnivore taxa responsible for gnaw damage to herbivore limb bones, Paleobiology. 9 (1983) 164–172.

[46] J.-C. Castel, Lʼinfluence des canidés sur la formation des ensembles archéologiques: caractérisation des destructions dues au loup, Revue de Paléobiologie. 23 (2004) 675–693.

[47] M. Domínguez-Rodrigo, C.P. Egeland, T.R. Pickering, Equifinality in carnivore tooth marks and the extended concept of archaeological palimpsests: implications for models of passive scavenging by early hominid, in: Breathing Life into Fossils: Taphonomic Studies in Honor of C.K. (Bob) Brain, Stone Age Institute Press, Gosport, Indiana, 2007: pp. 255–267.

[48] M. Domínguez-Rodrigo, A.O. Gidna, J. Yravedra, C. Musiba, A comparative neo-taphonomic study of felids, hyaenids and canids: an analogical framework based on long bone modification patterns, Journal of Taphonomy. 10 (2012) 151–170.

[49] M. Domínguez-Rodrigo, T.R. Pickering, A multivariate approach for discriminating bone accumulations created by spotted hyenas and leopards: harnessing actualistic data from East and southern Africa, Journal of Taphonomy. 8 (2010) 155–179.

[50] J. Yravedra, L. Lagos, F. Bárcena, A taphonomic study of wild wolf (*Canis lupus*) modification of horse bones in northwestern Spain, Journal of Taphonomy. 9 (2011) 37–65.

[51] J. Yravedra, M. Andrés, P. Fosse, J.-P. Besson, Taphonomic analysis of small ungulates modified by fox (*Vulpes vulpes*) in southwestern Europe, Journal of Taphonomy. 21 (2014) 37–67.

[52] A. Nascou, E. Morin, Arctic wolf and spotted hyena gnawing damage on an experimental faunal assemblage, Journal of Taphonomy. 12 (2014) 1–36.

[53] N. Sala, J.L. Arsuaga, G. Haynes, Taphonomic comparison of bone modifications caused by wild and captive wolves (*Canis lupus*), Quaternary International. 330 (2014) 126–135. https://doi.org/10.1016/j.quaint.2013.08.017.

[54] P. Fosse, N. Selva, W. Smietana, H. Okarma, A. Wajrak, J.-B. Fourvel, S. Madelaine, I.C. Montserrat Esteban-Nadal, J. Yravedra, J.-P. Brugal, A. Prucca, G. Haynes, Bone modification by modern wolf (*Canis lupus*): a taphonomic study from their natural feeding places, Journal of Taphonomy. 10 (2012) 197–217.

[55] F.M. Martín, L.A. Borrero, A puma lair in southern Patagonia: implications for the archaeological record, Current Anthropology. 38 (1997) 453–461. https://doi.org/10.1086/204634.

[56] G. Haynes, Utilization and skeletal disturbances of North American prey carcasses, ARCTIC. 35 (1982) 266–281. https://doi.org/10.14430/arctic2325.

[57] C.W. Marean, L.M. Spencer, R.J. Blumenschine, S.D. Capaldo, Captive hyaena bone choice and destruction, the Schlepp effect and Olduvai archaeofaunas, Journal of Archaeological Science. 19 (1992) 101–121. https://doi.org/10.1016/0305-4403(92)90009-R.

[58] C.C. Burke, Neotaphonomic analysis of the feeding behaviors and modification marks produced by North American carnivores, Journal of Taphonomy. 11 (2013) 1–20.

[59] A. Aranburu, J.L. Arsuaga, N. Sala, The stratigraphy of the Sima de los Huesos (Atapuerca, Spain) and implications for the origin of the fossil hominin accumulation, Quaternary International. 433 (2017) 5–21. https://doi.org/10.1016/j.quaint.2015.02.044.

[60] T.N. Bailey, The African Leopard: Ecology and Behavior of a Solitary Felid, Columbia University Press, New York, 1993.

[61] T.M. Caro, Cheetahs of the Serengeti Plains: Group Living in an Asocial Species, University of Chicago Press, Chicago, 1994.
